# Supplementary material for: Could forage peanut in low proportion replace N fertilizer in livestock systems?
Source: PLoS One. 2021 Mar 3;16(3):e0247931. doi: 10.1371/journal.pone.0247931 (PMC7928512; doi:10.1371/journal.pone.0247931)

PLOS ONE

Impact of N fertilization or the presence of forage peanut on the herbage structure of palisadegrass under grazing

--Manuscript Draft--

**Manuscript Number:**	PONE-D-20-17268
**Article Type:**	Research Article
**Full Title:**	Impact of N fertilization or the presence of forage peanut on the herbage structure of palisadegrass under grazing
**Short Title:**	Impact of N fertilization or the presence of forage peanut on the herbage structure of palisadegrass
**Corresponding Author:**	Vanessa Zirondi Longhini, M.D.<br>Universidade Estadual Paulista Julio de Mesquita Filho - Campus de Jaboticabal<br>Jaboticabal, BRAZIL
**Keywords:**	legume; grasses; nitrogen fertilization; nitrogen fixation; palisadegrass-forage peanut mixtures; pasture management; urea fertilizer
**Abstract:**	Palisadegrass [Urochloa brizantha (Hochst. ex A. Rich.) R. D. Webster cv. ‘Marandu’] is widely used in Brazil and typically managed with little or no N fertilizer, leading to pasture decline. The relationship between beef price and fertilizer cost does not favor fertilizer use in pastures. Legume inclusion is an alternative to add N. This study evaluated herbage responses to N inputs and vegetation types during the establishment, under rotational stocking. Treatments included palisadegrass-forage peanut (Arachis pintoi Krapov. & W.C. Greg.) mixture (mixed), unfertilized palisadegrass (control), and palisadegrass fertilized with 150 kg N ha-1 yr-1 (fertilized). Treatments were applied for two grazing trial with five grazing cycles (GC) evaluations. Response variables included herbage mass, herbage accumulation, morphological components, total aboveground N (TAGN), and contribution of biological N2 fixation (BNF). Pre-grazing herbage mass was greater for fertilized palisadegrass [5830 kg dry matter (DM) ha-1] than for the palisadegrass-forage peanut mixture (3880 kg DM ha-1), while the unfertilized palisadegrass (4400 kg DM ha-1) did not differ from the mixed pasture. Nitrogen fertilizer increased leaf mass (2470 kg DM ha-1) compared with the control and mixed treatments (1700 and 1310 kg DM ha-1, respectively). The %Ndfa for forage peanut ranged from 79 to 85% and BNF ranged from 0.7 to 5.5 kg N ha-1 cycle-1. Overall, benefits from peanut were minimal because legume percentage was <10%. Beginning a grazing cycle before 95% canopy light interception or imposing a shorter post-grazing stubble height may aid in reducing shading to the decumbent-growing forage peanut when planted with palisadegrass.
**Order of Authors:**	Vanessa Z. Longhini, M.D.<br>Abmael S. Cardoso<br>Andressa S. Berça<br>Robert M. Boddey<br>Ricardo A. Reis<br>José C.B. Dubeux<br>Ana C. Ruggieri
**Additional Information:**	
**Question**	**Response**
**Financial Disclosure**<br><br>Enter a financial disclosure statement that describes the sources of funding for the work included in this submission. Review	The authors were supported by São Paulo Research Foundation – FAPESP (VZL: grant # 2016/11086-1; ASC: grant# 2017/11274-5, and RAR: thematic grant # 2015/16631-5, http://www.fapesp.br/) and by Cientista de Nosso Estado for author RMB from the Rio de Janeiro State Research Foundation – FAPERJ (http://www.faperj.br/). This work was funded by National Council for Scientific and

Powered by Editorial Manager® and ProduXion Manager® from Aries Systems Corporation

the submission guidelines for detailed requirements. View published research articles from *PLOS ONE* for specific examples.<br><br>This statement is required for submission and **will appear in the published article** if the submission is accepted. Please make sure it is accurate.<br><br>**Unfunded studies**<br>Enter: *The author(s) received no specific funding for this work.*<br><br>**Funded studies**<br>Enter a statement with the following details:<br>• Initials of the authors who received each award<br>• Grant numbers awarded to each author<br>• The full name of each funder<br>• URL of each funder website<br>• Did the sponsors or funders play any role in the study design, data collection and analysis, decision to publish, or preparation of the manuscript?<br>• **NO** - Include this sentence at the end of your statement: *The funders had no role in study design, data collection and analysis, decision to publish, or preparation of the manuscript.*<br>• **YES** - Specify the role(s) played.<br><br>* typeset	Technological Development – CNPq (RMB: grant # 404169/2013-9, http://www.cnpq.br/). The funders had no role in study design, data collection and analysis, decision to publish, or preparation of the manuscript.
**Competing Interests**<br><br>Use the instructions below to enter a competing interest statement for this submission. On behalf of all authors, disclose any competing interests that could be perceived to bias this work—acknowledging all financial support and any other relevant financial or non-financial competing interests.<br><br>This statement **will appear in the published article** if the submission is accepted. Please make sure it is accurate. View published research articles from *PLOS ONE* for specific examples.	The authors have declared that no competing interests exist.

Powered by Editorial Manager® and ProduXion Manager® from Aries Systems Corporation

**NO authors have competing interests**<br><br>Enter: *The authors have declared that no competing interests exist.*<br><br>**Authors with competing interests**<br><br>Enter competing interest details beginning with this statement:<br><br>*I have read the journal's policy and the authors of this manuscript have the following competing interests: [insert competing interests here]*<br><br>* typeset	
**Ethics Statement**<br><br>Enter an ethics statement for this submission. This statement is required if the study involved:<br><br>• Human participants<br>• Human specimens or tissue<br>• Vertebrate animals or cephalopods<br>• Vertebrate embryos or tissues<br>• Field research<br><br>Write "N/A" if the submission does not require an ethics statement.<br><br>General guidance is provided below. Consult the submission guidelines for detailed instructions. **Make sure that all information entered here is included in the Methods section of the manuscript.**	The procedures involving animals were approved by the Institutional Animal Care and Use Committee at São Paulo State University, SP, Brazil (No. 10356/14-CEUA).

Powered by Editorial Manager® and ProduXion Manager® from Aries Systems Corporation

**Format for specific study types**<br><br>**Human Subject Research (involving human participants and/or tissue)**<br>• Give the name of the institutional review board or ethics committee that approved the study<br>• Include the approval number and/or a statement indicating approval of this research<br>• Indicate the form of consent obtained (written/oral) or the reason that consent was not obtained (e.g. the data were analyzed anonymously)<br><br>**Animal Research (involving vertebrate animals, embryos or tissues)**<br>• Provide the name of the Institutional Animal Care and Use Committee (IACUC) or other relevant ethics board that reviewed the study protocol, and indicate whether they approved this research or granted a formal waiver of ethical approval<br>• Include an approval number if one was obtained<br>• If the study involved *non-human primates*, add *additional details* about animal welfare and steps taken to ameliorate suffering<br>• If anesthesia, euthanasia, or any kind of animal sacrifice is part of the study, include briefly which substances and/or methods were applied<br><br>**Field Research**<br><br>Include the following details if this study involves the collection of plant, animal, or other materials from a natural setting:<br>• Field permit number<br>• Name of the institution or relevant body that granted permission	
**Data Availability**<br><br>Authors are required to make all data underlying the findings described fully available, without restriction, and from the time of publication. PLOS allows rare exceptions to address legal and ethical concerns. See the PLOS Data Policy and FAQ for detailed information.	Yes - all data are fully available without restriction

A Data Availability Statement describing where the data can be found is required at submission. Your answers to this question constitute the Data Availability Statement and **will be published in the article**, if accepted.<br><br>**Important:** Stating 'data available on request from the author' is not sufficient. If your data are only available upon request, select 'No' for the first question and explain your exceptional situation in the text box.<br><br>Do the authors confirm that all data underlying the findings described in their manuscript are fully available without restriction?	
**Describe where the data may be found in full sentences. If you are copying our sample text, replace any instances of XXX with the appropriate details.**<br><br>• If the data are **held or will be held in a public repository**, include URLs, accession numbers or DOIs. If this information will only be available after acceptance, indicate this by ticking the box below. For example: *All XXX files are available from the XXX database (accession number(s) XXX, XXX.).*<br>• If the data are all contained **within the manuscript and/or Supporting Information files**, enter the following: *All relevant data are within the manuscript and its Supporting Information files.*<br>• If neither of these applies but you are able to provide **details of access elsewhere**, with or without limitations, please do so. For example:<br><br>*Data cannot be shared publicly because of [XXX]. Data are available from the XXX Institutional Data Access / Ethics Committee (contact via XXX) for researchers who meet the criteria for access to confidential data.*<br><br>*The data underlying the results presented in the study are available from (include the name of the third party*	Data will be available after acceptance.

and contact information or URL).<br>• This text is appropriate if the data are owned by a third party and authors do not have permission to share the data.<br><br>* typeset	
Additional data availability information:	

Manuscript Click here to download Manuscript Manuscript_Vanessa.docx

Impact of N fertilization or the presence of forage peanut on the herbage structure of palisadegrass under grazing

Vanessa Z. Longhini[1*], Abmael S. Cardoso[1], Andressa S. Berça[1], Robert M. Boddey[2], Ricardo A. Reis[1], José C.B. Dubeux[3], Jr., Ana C. Ruggieri[1]

[1] Department of Animal Science, São Paulo State University, via de acesso Professor Paulo Donato Castellane, Jaboticabal, SP, 14884-900, Brazil

[2] Embrapa Agrobiologia, Antiga Rodovia Rio-São Paulo, km 47. Seropédica, RJ, 23891-000, Brazil

[3] University of Florida, North Florida Research and Education Center, Marianna, FL, 32446, United States

* Corresponding author: vanessa.longhini@unesp.br (VZL)

Abstract

Palisadegrass [*Urochloa brizantha* (Hochst. ex A. Rich.) R. D. Webster cv. ‘Marandu’] is widely used in Brazil and typically managed with little or no N fertilizer, leading to pasture decline. The relationship between beef price and fertilizer cost does not favor fertilizer use in pastures. Legume inclusion is an alternative to add N. This study evaluated herbage responses to N inputs and vegetation types during the establishment, under rotational stocking. Treatments included palisadegrass-forage peanut (*Arachis pintoi* Krapov. & W.C. Greg.) mixture (mixed), unfertilized palisadegrass (control), and palisadegrass fertilized with 150 kg N ha^{-1} yr^{-1} (fertilized). Treatments were applied for two grazing trial with five grazing cycles

(GC) evaluations. Response variables included herbage mass, herbage accumulation, morphological components, total aboveground N (TAGN), and contribution of biological N_2 fixation (BNF). Pre-grazing herbage mass was greater for fertilized palisadegrass [5830 kg dry matter (DM) ha^{-1}] than for the palisadegrass-forage peanut mixture (3880 kg DM ha^{-1}), while the unfertilized palisadegrass (4400 kg DM ha^{-1}) did not differ from the mixed pasture. Nitrogen fertilizer increased leaf mass (2470 kg DM ha^{-1}) compared with the control and mixed treatments (1700 and 1310 kg DM ha^{-1}, respectively). The %Ndfa for forage peanut ranged from 79 to 85% and BNF ranged from 0.7 to 5.5 kg N ha^{-1} $cycle^{-1}$. Overall, benefits from peanut were minimal because legume percentage was <10%. Beginning a grazing cycle before 95% canopy light interception or imposing a shorter post-grazing stubble height may aid in reducing shading to the decumbent-growing forage peanut when planted with palisadegrass.

Introduction

In Brazil, grasses of the genus *Urochloa* cover an estimated 80 million ha. ‘Marandu’ palisadegrass [*Urochloa brizantha* (Hochst. ex A. Rich.) R. D. Webster cv. syn. *Brachiaria brizantha* (Hochst. ex A. Rich.) Stapf.], the most used palisadegrass cultivar, is estimated to cover 56% of these area [1]. Low-input systems prevail in Brazilian livestock operations, so limestone, gypsum, and chemical fertilizer applications are rare. The combination of low soil fertility, lack of chemical fertilization, and overgrazing results in pasture degradation or pasture decline [2].

Nitrogen is a key element to improve pasture productivity [3] and many warm-season C_4 grasses respond positively to N fertilization increasing herbage accumulation and improving nutritive value. However, this response varies with the environment, species, and

management practices. Herbage accumulation of palisadegrass increased with increasing N fertilizer application from 90 to 270 kg ha^{-1}, but there was a diminished increase in herbage accumulation per unit of N applied, as the amount of N fertilizer increased [4]. In this case, tripling the amount of N applied increased herbage accumulation only 1.18 times and increased potential for negative environmental impacts. Nitrogen fertilizer is manufactured using fossil fuel, which gives it a significant carbon footprint [5]. Furthermore, N fertilizer may be lost as nitric oxide (NO also called nitrogen oxide), nitrogen dioxide (NO_2), nitrous oxide (N_2O), di-nitrogen (N_2), ammonia (NH_3), or via nitrate (NO_3^-) leaching [6, 7]. These processes may contribute to climate change (e.g., N_2O emission) or increase nitrate concentration in surface water bodies and groundwater.

Integrating legumes into grazing systems is an alternative to reduce the negative environmental impacts caused by N fertilization [8]. Legumes add N to the system via symbiotic biological dinitrogen fixation (BNF) between legume and rhizobium bacteria in root nodules [9]. In addition to reduced negative environmental impacts, legumes can also reduce production costs compared with N fertilizer [8, 10].

The use of grass-legume mixed pastures in tropical regions is less commonplace than in temperate regions. In North America, mixing grasses and legumes is a more common practice [11], but it is still currently being investigated [12, 13]. In Brazil, few studies have evaluated grass-legume mixtures under grazing. Challenges to legume use have been attributed to poor establishment and persistence compared with grasses [10, 14, 15]. Forage peanut (*Arachis pintoi* Krapov. & W.C. Greg. cv. Amarillo) is a legume that has a good potential to grow in mixtures in Brazil because it persists well when mixed with fast-growing grasses such as *Urochloa* [16, 17]. A stoloniferous and prostrate growth habit makes the forage peanut ideal for grazing because its meristems are protected from grazing animals [18]. In tropical

conditions, Tamele [15] reported that palisadegrass managed at a 20-cm canopy height improved forage peanut establishment in mixed systems. However, further research is necessary to determine if N from forage peanut growing in mixture with a grass is able to replace synthetic N fertilizer, especially during the establishment period.

We hypothesized that during the establishment of forage peanut with non-N fertilized grass the herbage structures such as herbage accumulation, green herbage mass, leaf mass, and leaf:stem ratio would be improved through BNF compared with unfertilized grass in a monoculture. We also hypothesized that mixed swards would have similar herbage accumulation compared to N fertilized swards. Therefore, this study evaluated the performance of palisadegrass under three sward management strategies (unfertilized, fertilized with 150 kg N ha^{-1} yr^{-1}, or mixed with forage peanut), aiming to identify how different N inputs may affect pasture responses during the establishment period. A low presence of forage peanut in the present study occurred during the establishment period. However, we decided to continue the experiment to test if a low amount of forage peanut mixture with palisadegrass would result in any contribution to the mixed system.

Materials and methods

Experimental site

This study was conducted at Sao Paulo State University, Jaboticabal, Sao Paulo, Brazil (21°14’05” S, 48°17’09” W; 615 m above sea level) during two grazing trials (November 2016 to March 2017 and December 2017 to April 2018). The soil at the experimental site is classified as an Oxisol [19]. Soil samples (0- to 20-cm depth) collected in 2014, 2017, and 2018 indicated the following means: pH ($CaCl_2$) 5.3; organic matter 32.4 g kg^{-1}; cation

exchange capacity 74.8 mmolc dm^{-3}; P (ion-exchange resin extraction method) 10.9 mg dm^{-3}; Mehlich^{-1} extractable Ca 28.3 mmolc dm^{-3}; Mehlich^{-1} extractable Mg 9.7 mmolc dm^{-3}; Mehlich^{-1} extractable K 4.2 mmolc dm^{-3}; base saturation 561 g kg^{-1}, respectively [20]. Soil texture was 340 g kg^{-1} sand, 140 g kg^{-1} silt and 520 g kg^{-1} clay [22]. Rainfall and mean temperature data from the experimental site were extracted from a dataset belonging to the Agrometeorology section of the Department of Exact Sciences (Fig 1).

Fig 1. Monthly rainfall and ~~monthly mean~~ air temperatures (maximum, mean, and minimum) from November 2016 to May 2018 in Jaboticabal, Brazil. Arrows indicate the nitrogen application (150 kg N ha^{-1} yr^{-1}, split in three equal applications) in the fertilized treatment.

Treatments and experimental design

There were three treatments (sward management strategies, SM) with four replications, arranged in a completely randomized design with plot size of 20 × 60 m. Treatments were 1) control - ‘Marandu’ palisadegrass in a monoculture, unfertilized (without N fertilizer or legume); 2) fertilized - ‘Marandu’ palisadegrass fertilized with 150 kg N ha^{-1} year^{-1} (urea), split into three equal applications; and 3) mixed - palisadegrass mixed with forage peanut cv. ‘Amarillo’ and no N fertilizer, during the establishment period. These treatments were evaluated for two grazing trials with five grazing cycle (GC) evaluations: 19-30 Nov. 2016 and 4-15 Dec. 2017 (GC1), 10-21 Dec. 2016 and 3-14 Jan. 2018 (GC2), 9-20 Jan. 2017 and 5-16 Feb. 2018 (GC3), 6-17 Feb. 2017 and 12-23 Mar. 2018 (GC4), and 13-24 Mar. 2017 and 23 Apr.-4 May 2018 (GC5). Nitrogen was applied to the fertilized treatment in November, January, and March each grazing trial (50 kg N ha^{-1} per application).

Forage peanut presents a slow establishment when mixed with aggressive-growing grasses, however, over time this legume is able to compete and enhance its contribution in mixed system [21]. The low presence of forage peanut during the establishment period occurred in the present study. Nevertheless, we decided to continue the experiment to test if a small amount of forage peanut mixture with grass resulted in any contribution to the mixed system.

Plot establishment and management

In March 2014, existent 'Tifton 85' bermudagrass (*Cynodon* spp.) was sprayed with 2880 g a.i. ha^{-1} of glyphosate [isopropylamine salt of N- (phosphonomethyl) glycine] in order to eradicate the existing vegetation. In October 2014, glyphosate plus 402 g a.i. ha^{-1} of 2,4-D amine (2,4-dichlorophenoxyacetic acid) were applied, and subsequently, a crusher and chopper (Tritton, model 1800) was used to remove dead residue. Grass monoculture was seeded subsequently with a row spacing of 0.45 m and a seeding rate of 10 kg ha^{-1} of pure live seed using a no-till seed drill (Semeato, model SHM 11-13). In the mixed treatment, forage peanut was simultaneously sown between rows of palisadegrass at a seeding rate of 8 kg ha^{-1} of pure live seeds using a seed drill (Jumil, model 2640 PD Exacta). After the seeding, all paddocks received 60 kg P, 20 kg S, 50 g Mo, 1 kg Cu and 1 kg B ha^{-1}, following recommendations of Werner [23]. At the beginning of the grazing trial in both years, single superphosphate (40 kg P ha^{-1}) and potassium chloride (50 kg K ha^{-1}) were applied. The grass-legume mixed pastures were not fertilized with N.

Before seeding, the three treatments were randomized and allocated in 12 experimental units (1200 m^2 each). Each experimental unit was split into three paddocks of 400 m^2. In September of each year, heifers were used to standardize the plots, and the paddocks were

defoliated using mob stocking [24]. Grazing for all treatments initiated when one of the treatments reached 95% light interception (LI) [25] determined using the canopy analyzer LAI-2200 (LI-COR®, USA). The average of light interception was 95, 96, and 90% for control, fertilized, and mixed treatments, respectively. Usually, the first grazing cycle occurred approximately 40 d after start of the rainy season (November). Five grazing cycles occurred in each grazing trial with a rest period between them varying from 21 to 42 d because growth rates varied seasonally resulting in different amounts of time required to reach 95% LI. Grazing periods lasted 3 d, being 1 d for each paddock. Grazing ended on a given paddock when a stubble height of 15 cm was reached. A minimum of five crossbred heifers (311 ± 27 and 234 ± 19 kg, 2016/2017 and 2017/2018, respectively) was used for grazing. Additional heifers were added as needed to achieve the target stubble height in 1 d of grazing on a given paddock. The same animal group was used for the three paddocks within an experimental unit. A reserve palisadegrass pasture was used to keep the animals during the rest periods.

Response variables

Herbage structures

All samples were collected on the second paddock on the second grazing day. Herbage accumulation rate [kg of dry mass (DM) ha^{-1} d^{-1}] was estimated as the difference between the pre-grazing herbage mass of the current grazing cycle and the post-grazing herbage mass of the previous grazing cycle (leaf + stem + dead mass was considered in this calculation), divided by the number of rest days between grazing cycles. Before putting the grazing animals on paddocks, the canopy height was estimated using a sward stick at 16 random points. The average canopy height was calculated and two samples representing the average

herbage mass were taken using 0.25-m^2 quadrats. The pre-herbage mass was harvested at 2 cm from the ground level. A subsample taken from the pre-herbage mass (fertilized and control treatment) was separated into leaf blade (leaf), stem plus sheath (stem), and dead material. When present, the inflorescence was included in the stem fraction. For the palisadegrass-forage peanut treatment, the entire sample was hand separated to measure the proportion of legume and grass (botanical composition), and their respective morphologic components (leaf, stem, and dead mass). For calculation of pre-grazing herbage mass (kg DM ha^{-1}), all aboveground biomass (leaf + stem + dead mass) was considered; for the mixed treatment, total grass plus legume was considered. The green herbage mass (kg DM ha^{-1}) included the green leaf and stem without the dead mass. These fractions and the remaining non-separated herbage were oven-dried at 55°C for 72 h to calculate leaf:stem ratio (L:S). Canopy bulk density was calculated by dividing pre-grazing herbage mass by canopy height (kg DM ha^{-1} cm^{-1}).

Total aboveground nitrogen, nitrogen derived from the atmosphere, and biological N_2 fixation by forage peanut

At the beginning of each grazing cycle, samples of total aboveground biomass of forage peanut and non-N_2-fixing reference plants (weeds) including billygoat-weed (*Ageratum conyzoides* L.), beggar-ticks (*Bidens pilosa* L.), coat-buttons (*Tridax procumbens* L.), gomphrena-weed (*Gomphrena celosioides* Mart.), cockroach berry (*Solanum capsicoides* All.), and sanguinarea [*Alternanthera ficoidea* (L.) P. Beauv.] were collected from the same area. Different reference plants were collected due to the low number of weeds in the paddock. Samples were ground using a Wiley mill (Marconi, Ma680) to pass through a 1-mm stainless steel screen. After samples were roller milled for 16 h [26], total N

concentration, abundance of ^{15}N and ^{13}C (‰) were analyzed using a mass spectrometer (model Delta V Advantage, Thermo Scientific) coupled to an elemental analyzer (model Flash 2000, Thermo Scientific) in the "John Day Stable Isotope Laboratory" at Embrapa Agrobiologia. The total aboveground N (TAGN, kg N ha^{-1}) was calculated multiplying the N concentration by the herbage accumulation of forage peanut ($TAGN_{FP}$, kg N ha^{-1}) and palisadegrass ($TAGN_{PG}$, kg N ha^{-1}). The percentage of N derived from the atmosphere (%Ndfa) for forage peanut was calculated using the equation described by Shearer and Kohl [27]:

$$\%Ndfa = \left(\frac{\delta^{15}N_{\mathrm{refence}} - \delta^{15}N_{legume}}{\delta^{15}N_{\mathrm{refence}} - \mathrm{B}}\right) \times 100$$

where $\delta^{15}N_{reference}$ is the $\delta^{15}N$ value detected in a reference plant (non-N fixing) growing in the same soil at the same grazing cycle in the palisadegrass-forage peanut paddock, $\delta^{15}N_{legume}$ is the $\delta^{15}N$ abundance of the forage peanut, and *B* is the $\delta^{15}N$ value of the legume obtained from dinitrogen fixation. We used the *B* value of −1.35‰ reported by Unkovich [28] for *Arachis hypogea* L. in Brazil. The $\delta^{15}N$ from reference plants ranged from 4.4 to 5.9‰ ± 0.6‰, with values changing with sampling date. The total contribution of N from BNF was calculated by multiplying $TAGN_{FP}$ by the %Ndfa [28].

Statistical analysis

All data were analyzed using the MIXED procedure of the SAS statistical package (SAS Inst. Inc., Cary, NC, USA). For the analysis of variance of palisadegrass, SM, GC and their interaction were fixed effects. The paddock (SM Year) were random effects. The GC were used as repeated measures. The Akaike information criterion (AIC) was used to choose the best covariance structure. Satterthwaite approximation was used to determine the

denominator degrees of freedom for the test of fixed effects. The LSMEANS were compared using the PDIFF procedure by Fisher's protected least significant difference (LSD) test at 5% probability. For the analysis of variance of forage peanut, the GC was the fixed effect. The paddock (GC Year) were the random effects. The Satterthwaite approximation was used to determine the denominator degrees of freedom for the test of fixed effects. The LSMEANS were compared using the PDIFF procedure by Fisher's protected least significant difference (LSD) test at 5% probability.

Results and discussion

Herbage structures

Nitrogen fertilizer enhanced pre-grazing herbage mass by 25 and 33% (P = 0.03) and green herbage mass by 32 and 41% (P = 0.01), compared with unfertilized palisadegrass and palisadegrass-forage peanut mixture, respectively (Table 1). Nitrogen is generally the most limiting nutrient for plant growth, enhances tillering [29] and is a major component of the chlorophyll molecule [30]. In similar conditions to those of this study, Delevatti [4] reported an increasing linear effect of N fertilization (0, 90, 180, and 270 kg N ha^{-1} year^{-1}) on palisadegrass herbage mass. In the Brazilian Cerrado, N fertilization (100 kg N ha^{-1}) increased palisadegrass herbage accumulation by 22% compared with unfertilized palisadegrass [31]. There was no difference for pre-grazing herbage mass and green herbage mass between the unfertilized palisadegrass and palisadegrass-forage peanut mixture, indicating that inclusion of the legume in the system did not negatively affect palisadegrass growth. We hypothesized that forage peanut could fix atmospheric N and supply N to

palisadegrass. However, the N input by the forage peanut in our study was not sufficient to promote growth of palisadegrass in the mixed system similar to the N-fertilized system.

Table 1. Pre-grazing herbage mass (PHM), green herbage mass (GHM), and herbage accumulation rate (HAR) of palisadegrass in monoculture, palisadegrass fertilized with 150 kg N ha^{-1} (split in three equal applications), or during the establishment of palisadegrass-forage peanut mixtures, for two grazing trials conducted during five grazing cycles.

	**Sward management strategies (SM)**			**SE**	**Grazing cycle (GC)**					**SE**	***P*-value**		
	**Control**	**Fertilized**	**Mixed**[‡]		**1**	**2**	**3**	**4**	**5**		**SM**	**GC**	**SM×GC**
PHM, kg DM ha^{-1}	4400ab[†]	5830a	3880b	490	4380	4460	4650	5060	4960	400	0.03	0.66	0.52
GHM, kg DM ha^{-1}	3030b	4440a	2600b	400	3370	3190	3450	3770	3020	330	0.01	0.19	0.83
HAR, kg DM ha^{-1} d^{-1}	67	83	51	14	NA	81a	56ab	78a	52b	13	0.32	0.04	0.10
Leaf:stem ratio	1.7	1.5	1.7	0.2	2.2a	1.4b	1.5b	1.5b	1.5b	0.1	0.78	0.01	0.15

[†] Means not followed by the same letter are different according to the PDIFF procedure by LSD test ($P \leq 0.05$).

[‡] Values of palisadegrass with forage peanut.

NA, not applicable (The GC1 was not evaluated, because the stubble mass after the standardization grazing in September was not performed).

SE, Standard error.

There was no difference between SM for herbage accumulation rate, however, GC influenced the response (Table 1). Ribeiro [32] reported a greater herbage accumulation rate when a bermudagrass-forage peanut mixture was fertilized with N compared with an unfertilized mixture; however, the herbage accumulation rates were similar when 100 or 200 kg N ha^{-1} was applied in the mixed systems. The authors suggested the dry season affected the legume recovery in the rainy season. In our study, the forage peanut proportion decreased in the last grazing cycle, by the drought conditions at the beginning of the transition of rainy season to dry season. Although we did not evaluate responses during the dry season, we noticed that the low rainfall during the dry season affected the presence of forage peanut. When the rainy season began, the forage peanut initiated growth, however, its recovery was slow. This may partially explain the lesser response of mixed systems in our study. In the same region, Delevatti [4] found a linear effect of N fertilizer rate on herbage accumulation rate of palisadegrass. However, in our study, the herbage accumulation rate of the N-fertilized treatment did not differ from mixed or unfertilized systems (Table 1). Sanchez [33] did not find difference in the herbage accumulation rate using bermudagrass in a monoculture or mixed with forage peanut (28 vs. 19 kg ha^{-1} d^{-1}), however, the authors observed difference between months due to limited rainfall and temperature. The weather conditions in different GC affected herbage accumulation. The values decreased from 81 to 52 kg DM ha^{-1} d^{-1} from GC2 to GC5, respectively. Average rainfall during the rest period between GC was 95, 60, 116, and 42 mm (for GC1 to GC2, GC2 to GC3, GC3 to GC4, and GC4 to GC5, respectively). Probably this pattern contributed to increasing the herbage accumulation rate in the GC2 and GC4, whereas no differences were observed in GC3 and GC5 due to less favorable temperature and rainfall (Fig 1).

There was no difference between SM for L:S ratio of green herbage mass, however, GC influenced the response (*P* < 0.01; Table 1). Greater L:S ratio was observed only in the GC1. The experimental period started two months after the grazing standardization, when the treatments reached 95% light interception. Probably the two months of rest period resulted in greater leaf mass. When the grazing cycle started, the rainfall increased, and the rest period reduced (~32 days), resulting in low leaf mass. There was a reduction of the L:S ratio after the GC1. It was expected that N-fertilized grasses would show greater L:S ratio by the increase in the leaf number and/or leaf elongation [34]. However, the N fertilization increased the leaf proportion as well as the stem proportion. Alencar [14] did not find a difference for L:S ratio for the palisadegrass in a mixture or a monoculture. Similar results were found in this study.

Morphological components of palisadegrass

Canopy height, leaf mass, and stem mass of palisadegrass at pre-grazing event were affected by SM and GC ($P \leq 0.05$; Table 2). Canopy height was greater in the N fertilized treatment (38 cm) than in the control (26 cm) and in the mixed sward (27 cm). Canopy height at 95% light interception was not constant for the SM studied. Therefore, canopy height can be a better strategy to compare monoculture and mixed pastures in future studies, at lower cost. Pereira [25] reported light interception between 90 and 95% with canopy height ranging from 26 to 32 cm for palisadegrass-forage peanut mixtures as the best grazing management to provide greater herbage accumulation rate, especially leaves. Brougham [35] indicated that 90 to 95% light interception leads to greatest herbage accumulation rates, especially in monoculture or mixed pastures. Canopy height control in mixed systems is important because there are two different plant species competing for light in the same area. Tamele [15] in a

harvesting study (weekly harvests) reported proportions of forage peanut ranging from 11 to 17% when the canopy height of palisadegrass was 30 cm; the optimum canopy height to increase the proportion of forage peanut was 20 cm. In the current study, the increase of canopy height might have decreased the proportion of legume, leaf mass, and subsequently $TAGN_{FP}$ and BNF. In pastures sampled seven years after planting, Gomes [36] found canopy height of palisadegrass in mixed system ranging between 25 (for 90% LI) and 30 cm (for 95% LI) without inhibiting the growth of the forage peanut in rotational stocking. Canopy height decreased in the GC5. This response may have occurred by the drought conditions at the beginning of the transition season (rainy to dry season), resulting in low green herbage mass (leaf and stem) and great dead mass in the GC5. This variation in canopy height suggests that there was no correlation between canopy height and light interception with the change from the rainy to the dry season. Canopy height is affected by management strategy, forage species, and season; therefore, the same LI may result in different canopy heights, mainly due to changes in plant architecture resulted from change in those factors. In our study, we used 95% LI target to start the grazing period, and the canopy height changed among GC (ranged from 26 to 33 cm). Pereira [25] reported similar results among evaluation period of palisadegrass-forage peanut (26 to 33 cm).

Table 2. Canopy height (CH), leaf herbage mass (LHM), stem herbage mass (SHM), and canopy bulk density (CD) of palisadegrass in monoculture, palisadegrass fertilized with 150 kg N ha^{-1} (split in three equal applications), or during the establishment of palisadegrass-forage peanut mixtures, for two grazing trials conducted during five grazing cycles.

	**Sward management strategies (SM)**			**SE**	**Grazing cycles (GC)**					**SE**	***P*- value**		
	**Control**	**Fertilized**	**Mixed**[‡]		**1**	**2**	**3**	**4**	**5**		**SM**	**GC**	**SM×GC**
CH, cm	26b[†]	38a	27b	3	33a	30a	32a	31a	26b	2	0.02	0.01	0.08
LHM, kg DM ha^{-1}	1700b	2470a	1310b	130	2230a	1670bc	1910ab	1950ab	1390c	130	0.01	0.01	0.62
SHM, kg DM ha^{-1}	1350ab	1960a	1020b	270	1090b	1320ab	1510ab	1770a	1540ab	210	0.05	0.03	0.79
CD, kg DM ha^{-1} cm^{-1}	170	160	138	10	136c	154abc	143bc	167ab	180a	11	0.07	0.02	0.16

[†] Means not followed by the same letter are different according to the PDIFF procedure adjusted by LSD test ($P \leq 0.05$).

[‡] Values for only palisadegrass (without forage peanut).

SE, Standard error.

Leaf mass of palisadegrass for the N-fertilized treatment was greater than the control and mixed (2470 vs. 1700 and 1310 kg DM ha^{-1}, respectively). The N is linked with plant growth and tissue turnover. An explanation is N fertilization may increase the leaf number and/or leaf elongation [34]. Pereira [37] reported increases of 25% in leaf elongation rate in palisadegrass fertilized with 150 kg N ha^{-1} when compared to unfertilized pastures during the summer. Fagundes [38] found a linear increase in leaf elongation rate for palisadegrass as N rates increased (75, 150, 225 and 300 kg N ha^{-1} yr^{-1}). Silva [1] reported that N fertilization from 50 to 250 kg N ha^{-1} yr^{-1} resulted in an increase in leaf proportion of Mulato II brachiariagrass hybrid (*U. ruziziensis* × *U. decumbens* × *U. brizantha*) from 22 to 25%. Leaf mass was greatest in GC1 and lowest in GC5 (2230 vs. 1390 kg DM ha^{-1}, respectively). The experimental period started two months after the grazing standardization. Probably this rest period resulted in greater leaf mass. When the grazing cycles started, the rainfall increased, and the rest period reduced (~32 days), resulting in low leaf mass (Table 2). The shortest rest period was between GC1 and GC2 (21 and 30 days), probably it was responsible for the low leaf mass in the GC2, whereas the GC5 increased the rest period (35 and 42 days) due to limited rainfall, resulting in great dead mass and low leaf mass.

Palisadegrass stem mass for the N-fertilized treatment was greater than the mixed treatment (1960 vs. 1020 kg DM ha^{-1}, respectively), which did not differ from the control treatment (1350 kg DM ha^{-1}). Nitrogen fertilization might have enhanced regeneration of the tiller population by increasing tiller appearance rate and tiller death; those factors can vary with season due to the weather conditions, such as temperature and rainfall [39]. In addition, the N fertilized treatment always reached first the 95% light interception than the other treatments, and this may have resulted in a competition for light between tillers, the plant will elongate the stem, especially in search of luminosity for the younger leaves [34]. The lowest stem mass was in GC1 and the greatest in GC4 (1090 vs. 1770 kg DM ha^{-1}). Average rainfall

during the rest period between GC3 and GC4 was 116 mm. During the rainy season, the tiller may have increased the population or elongation or become heavier to sustain more weight [34]. The stem is an important fraction for the tropical grasses because it is responsible for plant growth and leaf support to enhance light interception [38].

Canopy bulk density varied with GC (*P* = 0.02; Table 2) for palisadegrass, means ranged from 136 to 180 kg DM ha^{-1} cm^{-1}. The lowest means were observed in GC1, and the greatest was in GC5. These differences are reflections of the herbage mass and the canopy height promoted by stem elongation of the palisadegrass during the GC, which are influenced by the weather conditions (Fig 1). As rainfall decreased, the stem proportion increased and consequently canopy bulk density increased. Many elements may influence the canopy bulk density including plant species (C_4 and C_3), pasture management (defoliation intensity), and weather conditions [39].

There was a SM × GC interaction on the dead herbage mass of palisadegrass ($P < 0.01$; Fig 2). Within of GC, there was difference between SM in the CG2 and GC5 (Fig 2A). The N fertilization reduced the dead herbage mass in the GC2, however, in the GC5 the N fertilized increased the herbage dead mass. The mixed and control treatments were not different from each other in both GC. Within of SM, there was difference between GC for the control, fertilized, and mixed treatments (Fig 2B). Lower dead herbage mass was observed in the GC1, GC3, and GC4 for the fertilized, control, and mixed treatment, respectively. Whereas, greater dead herbage mass was observed in the GC2 and GC4 for the control treatment, and in the GC5 for the fertilized and mixed treatments. Dead herbage mass of palisadegrass ranged from 990 to 1590 kg DM ha^{-1}, 640 to 2670 kg DM ha^{-1}, and 1010 to 1700 kg DM ha^{-1} for control, fertilized, and mixed treatment, respectively. These responses may have occurred because the N fertilizer was applied in November, January, and March. The N fertilization combined with favorable weather conditions (temperature and rainfall) likely promoted

greater green mass and reduced the dead herbage mass in the GC2, while the N fertilization in March and drought conditions at the beginning of the transition season may have increased the dead herbage mass in the GC5. Nitrogen fertilization likely affected maturity stage in the last GC period, and may have hastened flowering stem elongation and leaf death compared to other treatments. Nitrogen fertilization might have enhanced regeneration of the tiller population by increasing tiller appearance rate and tiller death; those factors can vary with season due to the weather conditions, such as temperature and rainfall [39]. Pereira [37] reported increases of 22% in leaf appearance rate in palisadegrass fertilized with 150 kg N ha^{-1} when compared with unfertilized pastures during the summer. This acceleration in leaf appearance rate may be responsible for increasing the dead material later in the season in our study. In a tropical environment, Gomes [36] reported that dead herbage mass for palisadegrass-forage peanut mixtures did not change between grazing frequencies (initiated at 100, 95, and 90% LI or grazed every 42 d) and seasons, with an average of 2332 kg ha^{-1}. Our study showed lower dead mass for the mixed system, and there was a difference in the variable among CG, and it was affected mainly by the weather conditions and soil nutrients. Similar pattern occurred to control treatment.

Fig 2. Sward management strategies (SM) × grazing cycle (GC) interaction effect on dead herbage mass (mean ± SE bars) of palisadegrass during the establishment period. [†]Non-significant. Data are means across 2 yr, and 4 replicates (n = 8). Control (palisadegrass without nitrogen fertilizer or legume), Fertilized (palisadegrass fertilized with 150 kg N ha^{-1}, split in three equal applications), or Mixed (palisadegrass-forage peanut mixtures no N fertilizer). Different lowercase letter within GC (A) and SM (B) are different according to the PDIFF procedure by LSD test ($P \leq 0.05$). Sward management strategies: $P = 0.95$, Grazing cycle: $P < 0.01$, Sward management strategies × Grazing cycle: $P < 0.01$.

Morphological components of forage peanut

There were differences between GC for leaf and stem mass, but no differences were found for the dead mass of forage peanut. The dead herbage mass was consistently low, ranging from 4 to 22 kg DM ha^{-1} (P = 0.26; Table 3). In the dry season, leaf loss typically occurs for forage peanut [36], whereas during the rainy season there are greater growth rates due to greater leaf appearance rate and elongation [25], with less death occurring. In this study, the leaf mass of forage peanut ranged from 21 to 197 kg DM ha^{-1} (P = 0.03; Table 3), with the lowest value found in GC5. In these GC in southeastern Brazil the transition period between the rainy and dry season begins.

Table 3. Canopy height (CH), leaf herbage mass (LHM), stem herbage mass (SHM), dead herbage mass (DHM), and canopy bulk density (CD) during the establishment of forage peanut in a mixed system. The response variables are means across four replicates, and 2 yr (n = 8).

	**Grazing Cycle (GC)**					**SE**	***P*-value**
	**1**	**2**	**3**	**4**	**5**		
CH, cm	9	8	9	8	6	1	0.48
LHM, kg DM ha^{-1}	152a[†]	197a	153a	111a	21b	70	0.03
SHM, kg DM ha^{-1}	107a	181a	129a	129a	20b	65	0.05
DHM, kg DM ha^{-1}	4	12	22	9	10	6	0.26
CD, kg DM ha^{-1} cm^{-1}	27	44	29	27	11	10	0.39

[†] Means not followed by the same letter are different according to the PDIFF procedure by LSD test ($P \leq 0.05$).

SE, Standard error.

Stem mass of forage peanut ranged from 20 to 181 kg DM ha^{-1} ($P \leq 0.05$; Table 3). Gomes [36] reported that stem mass for forage peanut did not change with grazing frequency treatments or seasons. Our study showed a reduction in stem mass in GC5, probably due to

rainfall reduction and the beginning of the dry season. Our values are lower (113 vs. 766 kg ha^{-1}, respectively) than those reported by Gomes [36]. However, their pasture was established 7 yr before the experimental period. In this study, the forage peanut was not fully established yet because the experimental period began 2 yr after seeding. Pre-grazing herbage mass of forage peanut ranged from 249 to 390 kg DM ha^{-1}, decreasing in the GC5 to 51 kg DM ha^{-1}. Pereira [41] found 493 kg DM ha^{-1} of forage peanut mixed with palisadegrass in the rainy season, and the mixed treatment resulted in a liveweight gain per ha of 28.7% greater than in the palisadegrass N fertilized with 120 kg N ha^{-1}.

There was no difference between GC for canopy height, and canopy bulk density of forage peanut ($P > 0.05$; Table 3). Canopy height ranged from 6 to 9 cm, and the canopy bulk density ranged from 17 to 44 kg DM ha^{-1} cm^{-1}. Canopy bulk density of *Arachis glabrata* Benth. cultivars were influenced by cultivar, season, and grazing management [42]. High canopy bulk density may reduce the number of tongue sweeps, bite volume, and bite weight [43]. It can limit the selection of leaf and, consequently, reduces animal performance [44].

Botanical composition

The botanical composition differed between GC ($P < 0.01$; Fig 3). Weed presence was minimal, and the data are not reported. The greatest proportion of palisadegrass (98%) was found in GC5, and the lowest proportion (91%) was found in GC2. The greatest proportion of forage peanut (9%) was found in GC2, and the lowest proportion (2%) was found in GC5. The botanical composition in GC1, GC3, and GC4 averaged 93 and 7% of palisadegrass and forage peanut, respectively. The presence of forage peanut in the mixed system declined as the rainy season ended. Warm-season C_4 grasses are typically more competitive than legumes, shifting the balance in mixed swards towards the grass [14]. This competition can reduce the persistence of forage peanut in mixed systems. Grass establishment, growth, and

time to recover after grazing tend to be faster than for legumes [10]. In the Brazilian tropics, Tamele [15] reported a reduction in the proportion of forage peanut with increased canopy height (10, 20, 30, and 40 cm) of palisadegrass in a harvesting study (weekly harvests). The authors concluded that the canopy height of 20 cm provided the best proportion of legume (19 – 38%) in a mixed sward, without compromising the grass growth. The increase in grass canopy height can stimulate vertical growth of forage peanut, reducing the number of stolons and decreasing legume proportion in the mixed system. These changes in growth habit of forage peanut may limit its ability to compete with palisadegrass in mixed stands. In addition, the lower proportion of the legume in the botanical composition may be caused by the slow establishment [14]. Forage peanut growth may have been limited by shading from palisadegrass [15]. Santos [12] reported 57% rhizoma peanut in mixtures with bahiagrass 4 yr after seeding. It is possible that forage peanut available for use in Brazil is less vigorous during the establishment phase than rhizoma peanut. Studies in Florida USA show good results in the use of rhizoma peanut cultivars in mixed systems [12, 13]. *Arachis pintoi* has great tolerance to grazing, mainly because the growing points are protected from grazing animals, unlike most of the tropical legume species. Thus, it is possible to maintain residual leaf area even when the plant is subjected to continuous stocking and intense defoliation [45]. Although the proportion of forage peanut in the botanical composition was lower than the expected to mixed systems, in our study, the legume proportion in the dung ranged from 11.2 to 15.3% (data not shown). Considering the forage peanut digestibility ranged from 68.0 to 68.7 [36], probably the legume proportion in the diet of heifers may have been greater than in the botanical composition.

Fig 3. Effect of grazing cycle (GC) on botanical composition during the establishment of palisadegrass-forage peanut mixtures (*P* < 0.01). Data are means across 2 yr, and 4 replicates (n = 8). Different lowercase letters are different between GC, according to the PDIFF procedure by LSD test ($P \leq 0.05$).

Total aboveground nitrogen, nitrogen derived from the atmosphere, and biological N_2 fixation by forage peanut

The total aboveground N of forage peanut ($TAGN_{FP}$) was affected by GC ($P \leq 0.05$; Table 4), ranging from 1 to 7 kg N ha^{-1} $cycle^{-1}$. Greater N in herbage mass was present in GC1 and did not differ from GC2 and GC3. The low herbage accumulation of forage peanut resulted in low $TAGN_{FP}$. Leaf mass (Table 3) and presence of forage peanut in the botanical composition (Fig 3) were greater in GC1 to GC4. Total herbage accumulation of forage peanut for five GC was 613 kg DM ha^{-1}, resulting in a $TAGN_{FP}$ of 17 kg N ha^{-1} (GC1 to GC5). The $TAGN_{FP}$ decreased when the forage peanut in the herbage accumulation decreased (GC4 and GC5). Jaramillo [13] reported values of 0.7 kg N ha^{-1} $harvest^{-1}$ for forage peanut mixed with bahiagrass. Santos [12] reported TAGN for rhizoma peanut-bahiagrass mixtures of 50 kg N ha^{-1}. In our study, the forage peanut demonstrated low potential to compete with the palisadegrass, due to slow legume establishment. The nutrient competition between grass and legume, the lack of N fertilization in the first years of establishment of the mixed system, and the slow establishment of forage peanut may have resulted in low herbage mass of forage peanut. During the establishment period of grass-legume mixed pastures, N fertilization can be used considering that legumes have limited capability to fix atmospheric N_2 during initial developmental stages [46]. Two weeks after germination, Alencar [14] fertilized a palisadegrass-forage peanut mixture with 30 kg N ha^{-1}, but the amount of N fixation was small (~ 4.0 kg ha^{-1}) due to the slow growth of the forage peanut.

Table 4. Total aboveground N of palisadegrass ($TAGN_{PG}$) and forage peanut ($TAGN_{FP}$), nitrogen derived from the atmosphere (%Ndfa), and biological N_2 fixation (BNF) during the establishment of forage peanut in a mixed system. The response variables are means across four replicates, and 2 yr (n = 8).

	Grazing cycle						
	**1**	**2**	**3**	**4**	**5**	**SE‡**	***P*-value**
Palisadegrass							
$TAGN_{PG}$, kg N ha^{-1} $cycle^{-1}$	31a†	19b	18b	13b	19ab	4	0.05
Forage peanut							
$TAGN_{FP}$, kg N ha^{-1} $cycle^{-1}$	7a	3ab	4ab	2b	1b	2	0.05
%Ndfa	79	81	82	82	85	2	0.14
BNF, kg N ha^{-1} $cycle^{-1}$	5.5a	2.4ab	3.2ab	1.7b	0.7b	1.8	0.05

† Means not followed by the same letter are different according to the PDIFF procedure by LSD test ($P \leq 0.05$).

SE, Standard error.

The total aboveground N of palisadegrass ($TAGN_{PG}$) was affected by GC ($P = 0.04$; Table 4). The $TAGN_{PG}$ ranged from 13 to 31 kg N ha^{-1} $cycle^{-1}$. Greatest N aboveground was found in GC1 and the lowest in GC4. The difference in the rainfall and temperature (Fig 1) can explain the differences found between GC. Weather conditions influence herbage accumulation and N concentration in the grass and legume. Total herbage accumulation of palisadegrass for five GC was 9785 kg DM ha^{-1} (GC1 to GC5), resulting in a $TAGN_{PG}$ of 100 kg N ha^{-1}. The low proportion of legume in the mixed system may have resulted in low TAGN. The fibrous root systems of the C_4 plants compete for superficial moisture and soil nutrients when compared with taproot systems of C_3 plants [10]. Santos [12] found values of TAGN for bahiagrass mixed with rhizoma peanut of 6 kg N ha^{-1} $harvest^{-1}$.

There was a SM × GC effect on TAGN ($P < 0.01$; Fig 4). Within of GC, there was difference between SM in the CG1 and GC4 (Fig 4A). The N fertilization increased the TAGN in both GC. The mixed treatment was the lowest TAGN in the GC1, while in the GC4

did not differ from control treatment. Within of SM, there was difference between GC for the control and fertilized treatments, while the mixed treatment did not differ between GC (Fig 4B). The TAGN ranged from 22 to 76 kg N ha^{-1} $cycle^{-1}$, 34 to 113 kg N ha^{-1} $cycle^{-1}$, and 15 to 38 kg N ha^{-1} $cycle^{-1}$ for control, fertilized and mixed treatment, respectively. Greater N yields were found in GC1, being greater than other GC for both the control and fertilized treatments. Considering that TAGN is a product of forage N concentration and herbage accumulation, the N fertilizer directly affected results (Fig 1). There was no difference for TAGN between GC when forage peanut and palisadegrass were analyzed together. Santos [12] reported TAGN ranging from 6 to 40 kg N ha^{-1} $harvest^{-1}$ for rhizoma peanut-bahiagrass mixture and bahiagrass fertilized with 90 kg N ha^{-1} $harvest^{-1}$, respectively.

Fig 4. Sward management strategies (SM) × grazing cycle (GC) interaction effect on Total aboveground N (TAGN) (mean ± SE bars) during the establishment period. [†]Non-significant. Data are means across 2 yr, and four replicates (n = 8), three sward management strategies (SM): Control (palisadegrass without nitrogen fertilizer or legume), Fertilized (palisadegrass fertilized with 150 kg N ha^{-1}, split in three equal applications), or Mixed (palisadegrass-forage peanut mixtures no N fertilizer), and five grazing cycle (GC): GC1 to GC5. Different lowercase letter within GC (A) and SM (B) are different according to the PDIFF procedure by LSD test ($P \leq 0.05$). Sward management strategies: $P = 0.01$, Grazing cycle: $P < 0.01$, Sward management strategies × Grazing cycle: $P < 0.01$.

There was no difference between GC for %Ndfa for forage peanut ($P = 0.14$; Table 4). Values ranged from 79 to 85% in GC1 to GC5. Our study found values similar to the literature. Alencar [14] reported a Ndfa of ~80% for forage peanut. Dubeux [47] reported Ndfa ranging from 67 to 96% for seven rhizoma peanut cultivars. Santos [12] found %Ndfa

ranging from 60 to 82% for rhizoma peanut in a monoculture or mixed with bahiagrass. The authors observed that legumes in the mixture presented similar %Ndfa compared with legumes in monocultures.

The BNF varied with GC ($P \leq 0.05$; Table 4), and this was likely linked to the reduction of the leaves. The aboveground biomass is associated with the reduction of BNF [48]. Greater BNF was found in GC1, probably because of better weather conditions for plant growth (Fig 1). The total amount of N_2 fixation was 13.5 kg N ha^{-1} (GC1 to GC5). These results are relatively small due to the reduced growth of forage peanut (< 10% of the legume in the botanical composition) in the mixed system and mainly due to slow establishment. The proportion of 20 to 30% legume in the botanical composition of pastures may fix 50 to 100 kg N ha^{-1} yr^{-1} [46]. Jaramillo [13] found BNF ranging from 0 to 2.7 kg N ha^{-1} $harvest^{-1}$ for forage peanut-bahiagrass mixtures. Alencar [14] reported BNF of 3.4 kg N ha^{-1} for forage peanut. The authors concluded that during the legume establishment period, there was a reduced BNF due to the slow growth of the legume. A similar pattern was observed in our study. Other authors reported greater BNF with the legume inclusion in mixed systems [12]; however, some factors, such as difference between peanut cultivars, establishment time, and persistence may have influenced the proportion of forage peanut in the pasture, and this was reflected in the BNF. Additionally, in our study the pasture was seeded in 2014 and the trial performed in November 2016 to March 2017 and December 2017 to April 2018, 2 yr after the seeding. In the mixed management, we did not fertilize with N, and the competition for nutrients between legume and grass may have resulted in low results for both forages. Forage peanut mixed with palisadegrass is considered promising; however, significant competition that negatively affects forage peanut establishment occurs in early stages following planting [14]. Further studies need to evaluate seeding *Arachis* with machinery and applying N

fertilization during the early stages after planting to determine if legume establishment rate can improve.

Conclusions

The small amount of forage peanut in the mixed system presented similar results to the unfertilized palisadegrass in most of the response variables during the establishment period. Botanical composition fluctuated according to the grazing cycle, with forage peanut disappearance at the beginning of the transition from rainy to dry season. Biological N_2 fixation and $TAGN_{FP}$ decreased with the reduction of leaf mass, which was affected by the different weather conditions during the season. In this study, the forage peanut inclusion was not enough to guarantee better results of the mixed system when compared to the N fertilized palisadegrass. The N fertilization enhanced the pre-grazing herbage mass and green herbage mass of palisadegrass, especially the proportion of leaves.

The evaluation during the grazing cycle demonstrated the growth pattern of the grass in a tropical environment as well as forage peanut. However, the legume was less tolerant to lack of water with the beginning of the transition to the dry season. Different management strategies such as the beginning of the grazing cycle before 95% of light interception or lower stubble heights should be employed, considering that legumes (C_3) and grasses (C_4) require different management mainly during the establishment period.

Acknowledgements

The authors thank the members of UnespFOR (Brazilian forage Team) for the contributions during the field trial setup.

References

1. Silva VJ, Pedreira CGS, Sollenberger LE, Silva LS, Yasuoka JI, Almeida ICL. Canopy height and nitrogen affect herbage accumulation, nutritive value, and grazing efficiency of 'Mulato II' Brachiariagrass. Crop Sci. 2016; 56:2054–2061. doi: http://dx.doi.org/10.2135/cropsci2015.12.0764
2. Boddey RM, Macedo R, Tarré RM, Ferreira E, Oliveira OC, Rezende CP, et al. Nitrogen cycling in *Brachiaria* pastures: The key to understanding the process of pasture decline. Agric. Ecosyst. Environ. 2004; 103:389–403. doi: http://dx.doi.org/10.1016/j.agee.2003.12.010
3. Sollenberger LE, Newman YC, Vendramini JMB. General guidelines for managing pastures for dairy cows. Coop. Ext. Serv. Publ. AG162. Inst. Food Agric. Sci., Univ. Florida, Gainesville. 2009.
4. Delevatti LM, Cardoso AS, Barbero RP, Leite RG, Romanzini EP, Ruggieri AC, et al. Effect of nitrogen application rate on yield, forage quality, and animal performance in a tropical pasture. Sci. rep. 2019; 9.7596. https://doi.org/10.1038/s41598-019-44138-x
5. Gerber PJ, Steinfeld H, Henderson B, Mottet A, Opio C, Dijkman J, Falcucci A, Tempio G. Tackling climate change through livestock – A global assessment of emissions and mitigation opportunities. Food and Agriculture Organization of the United Nations (FAO), Rome. 2013.
6. Zaman M, Saggar S, Blennerhassett JD, Singh J. Effect of urease and nitrification inhibitors on N transformation, gaseous emissions of ammonia and nitrous oxide, pasture yield and N uptake in grazed pasture system. Soil Biol. Biochem. 2009; 41:1270–1280. doi; https://doi.org/10.1016/j.soilbio.2009.03.011
7. Zaman M, Nguyen ML, Šimek M, Nawaz S, Khan MJ, Babar MN, et al. Emissions of nitrous oxide (N_2O) and di-nitrogen (N_2) from the agricultural landscapes, sources, sinks,

and factors affecting N_2O and N_2 ratios. Greenhouse gases-emission, measurement and management (Ed. Guoxiang Liu), 1-32. 2012.

8. Muir JP, Pitman WD, Dubeux JC, Foster JL. The future of warm-season, tropical and subtropical forage legumes in sustainable pastures and rangelands. Afr. J. Range For. Sci. 2014; 31:187–198. doi: http://dx.doi.org/10.2989/10220119.2014.884165

9. Sprent JI. Evolving ideas of legume evolution and diversity: a taxonomic perspective on the occurrence of nodulation. New Phytol. 2007; 174:11–25. doi: http://dx.doi.org/10.1111/j.1469-8137.2007.02015.x

10. Muir JP, Pitman WD, Foster JL. Sustainable, low-input, warm-season, grass-legume grassland mixtures: Mission (nearly) impossible? Grass Forage Sci. 2011; 66:301–315. doi: http://dx.doi.org/10.1111/j.1365-2494.2011.00806.x

11. Williams MJ, Hammond AC, Kunkle WE, Spreen TH. Stocker Performance on Continuously Grazed Mixed Grass-Rhizoma Peanut and Bahiagrass Pastures. J. Prod. Agric. 1991; 4:19–24. doi: http://dx.doi.org/10.2134/jpa1991.0019

12. Santos ER, Dubeux JCB, Sollenberger LE, Blount ARS, Mackowiak C, DiLorenzo N, et al. Herbage responses and biological N_2 fixation of bahiagrass and rhizoma peanut monocultures compared with their binary mixtures. Crop Sci. 2018; 58:1–15. doi: http://dx.doi.org/10.2135/cropsci2018.02.0128

13. Jaramillo DM, Dubeux JCB, Mackowiak C, Sollenberger LE, DiLorenzo N, Rowland DL, et al. Annual and perennial peanut mixed with ‘Pensacola’ bahiagrass in North Florida." Crop Sci. 2018; 58:982–992. doi: http://dx.doi.org/10.2135/cropsci2017.09.0542

14. Alencar NM, Vendramini JMB, Santos AC, Silveira ML, Dubeux JCB, Sousa LF, et al. Herbage characteristics of pintoi peanut and palisadegrass established as monoculture or

mixed swards. Crop Sci. 2018; 58:1–7. doi: http://dx.doi.org/10.2135/cropsci2017.09.0538

15. Tamele OH, Sá OAAL, Bernardes TF, Lara MAS, Casagrande DR. Optimal defoliation management of brachiaria grass–forage peanut for balanced pasture establishment. Grass Forage Sci. 2018; 73:522–531. doi: http://dx.doi.org/10.1111/gfs.12332

16. Hernandez M, Argel PJ, Ibrahim MA, Mannetje LT. Pasture production, diet selection and liveweight gains of cattle grazing *Brachiaria brizantha* with or without *Arachis pintoi* at two stocking rates in the Atlantic zone of Costa Rica. Trop. Grassl. 1995; 29:134–141.

17. Andrade CMS, Garcia R, Valentim JF, Pereira OG. Productivity, utilization efficiency and sward targets for mixed pastures of marandugrass, forage peanut and tropical kudzu. R. Bras. Zootec. 2012; 41:512–520. doi: http://dx.doi.org/10.1590/S1516-35982012000300006

18. Andrade CMS, Garcia R, Valentim JF, Pereira OG. Response of *Arachis pintoi* to grazing intensity when associated with different grasses. In: International Grassland Congress, Dublin. Proceedings. Wageningen: Wageningen Academic Publishers, 2005. p. 347.

19. USDA. Soil Survey Staff. 2014. Keys to Soil Taxonomy, 12th ed. USDA-Natural Resources Conservation Service, Washington, DC. 2014.

20. van Raij B, Andrade JC, Cantarella H, Quaggio JA. Análise química para avaliação da fertilidade de solos tropicais. IAC, Campinas; 2001. (In Portuguese).

21. Argel PJ, Pizarro EA. Germplasm case study: *Arachis pintoi*. In.: Pastures for the Tropical Lowlands: CIAT's (Centro International de Agricultura Tropical) Contribution. Cali, Colombia; 1992.

22. Claessen MEC. Manual de métodos de análise de solo/Centro Nacional de Pesquisa de Solos. 2 ed. rev. Atual. Rio de Janeiro, EMBRAPA-CNPS. Documentos; 1997. (In Portuguese).

23. Werner JC, Paulino VT, Cantarella H, Andrade NO, Quaggio JA. Forrageiras. In: van Raij B, Cantarella H, Quaggio JA, Furlani AMC. Recomendações de adubação e calagem para o Estado de São Paulo. Campinas: Instituto Agronômico, 1996p. 263-273. (In Portuguese).

24. Allen VG, Batello C, Berretta EJ, Hodgson J, Kothmann M, Li X, et al. An international terminology for grazing lands and grazing animals. Grass Forage Sci. 2011; 66:2–28. doi: http://dx.doi.org/10.1111/j.1365-2494.2010.00780.x

25. Pereira JC, Gomes FK, Oliveira MD, Lara MA, Bernardes TF, Casagrande DR. Defoliation management affects morphogenetic and structural characteristics of mixed pastures of brachiaria grass and forage peanut. Afr. J. Range For. Sci. 2017; 34:13–19. doi: https://doi.org/10.2989/10220119.2017.1315960

26. Arnold SL, Schepers JS. A simple roller-mill grinding procedure for plant and soil samples. Commun. Soil Sci. Plan. 2004; 35:537–545. doi: http://dx.doi.org/10.1081/CSS-120029730

27. Shearer G, Kohl D. N_2-fixation in field settings: Estimations based on natural ^{15}N abundance. Aust. J. Plant Physiol. 1986; 13:699–756. doi: http://dx.doi.org/10.1071/PP9860699

28. Unkovich M, Herridge D, Peoples M, Cadisch G, Boddey R, Giller K, et al. Measuring plant-associated nitrogen fixation in agricultural systems. In: Unkovich M, Herridge D, Peoples M, Cadisch G, Boddey R, Giller K, et al. Australian Centre for International Agricultural Research (ACIAR), 2008.

29. Silva DRG, Costa KAP, Faquin V, Oliveira IP, Bernardes TF. Rates and sources of nitrogen in the recovery of the structural and productive characteristics of marandu grass. Rev. Cienc. Agron. 2013; 44, 184–191. doi: http://dx.doi.org/10.1590/S1806-66902013000100023 (Abstract in English)

30. Chapman SC, Barreto HJ. Using a chlorophyll meter to estimate specific leaf nitrogen of tropical maize during vegetative growth. Agron. J. 1997; 89:557–562. doi: http://dx.doi.org/10.2134/agronj1997.00021962008900040004x

31. Dupas E, Buzetti S, Rabêlo FHS, Sarto AL, Cheng NC, Galindo FS, et al. Nitrogen recovery, use efficiency, dry matter yield, and chemical composition of palisade grass fertilized with nitrogen sources in the Cerrado biome. Aust. J. Crop Sci. 2016; 10:1330–1338. doi: http://dx.doi.org/10.21475/ajcs.2016.10.09.p7854

32. Ribeiro OL, Cecato U, Roma CFC, Faveri JC, Gomes JAN, Barbero ML. Forage production and performance animal in coastcross intercropping or not with *Arachis pintoi*, with or without nitrogen. Acta Sci. Anim. Sci. 2008; 30:371–378. doi: http://dx.doi.org/10.4025/actascianimsci.v30i4.6466 (Abstract in English)

33. Sanchez J, Vendramini J, Sollenberger LE, Silveira ML, Dubeux JC, Moriel P, et al. Forage characteristics of bermudagrass pastures overseeded with Pintoi peanut and grazed at different stubble heights. Crop Sci. 2018; 58:1808–1816. doi: http://dx.doi.org/10.2135/cropsci2018.01.0007

34. Santana SS, Brito LF, Azenha MV, Oliveira AA, Malheiros EB, Ruggieri AC, Reis RA. Canopy characteristics and tillering dynamics of Marandu palisade grass pastures in the rainy–dry transition season. Grass Forage Sci. 2016; 72:261–270. doi: https://doi.org/10.1111/gfs.12234

35. Brougham RW. Interception of light by the foliage of pure and mixed stands of pasture plants. Aust. J. Agric. Res. 1958; 9:39–52. doi: https://doi.org/10.1071/AR9580039

36. Gomes FK, Oliveira MDBL, Homem BGC, Boddey RM, Bernardes TF, Gionbelli MP, et al. Effects of grazing management in brachiaria grass-forage peanut pastures on canopy structure and forage intake. J. Anim. Sci. 2018; 96:3837–3849. doi: http://dx.doi.org/10.1093/jas/sky236

37. Pereira LET, Paiva AJ, Guarda VDÁ, Pereira PM, Caminha FO, Silva SC. Herbage utilisation efficiency of continuously stocked marandu palisade grass subjected to nitrogen fertilisation. Sci. Agríc. 2015; 72:114–123. doi: http://dx.doi.org/10.1590/0103-9016-2014-0013

38. Fagundes JL, Fonseca DM, Mistura C, Morais RV, Vitor CMT, Gomide JA, et al. Morphogenetical and structural characteristics of the signalgrass in a nitrogen fertilized pasture evaluated over the seasons of the year. R. Bras. Zootec. 2006; 35:21–29. http://dx.doi.org/10.1590/S1516-35982006000100003. (Abstract in English)

39. Caminha FO, Silva SC, Paiva AJ, Pereira LET, Mesquita P, Guarda VDA. Stability of tiller population of continuously stocked marandu palisade grass fertilized with nitrogen. Pesq. Agropec. Bras. 2010; 45:213–220. doi: http://dx.doi.org/10.1590/S0100-204X2010000200013 (Abstract in English)

40. Sollenberger LE, Burns JC. Canopy characteristics, ingestive behaviour and herbage intake in cultivated tropical grasslands. In: Gomide JA, Mattos WRS, Silva SC, Proceedings of the 19th International Grassland Congress, São Pedro, Brazil. 10–21 Feb. 2001. Brazilian Soc. Anim. Husbandry, Piracicaba, Brazil. 2001, pp. 321–327.

41. Pereira JM, Rezende CDP, Borges AMF, Homem BGC, Casagrande DR, Macedo TM, et al. Production of beef cattle grazing on *Brachiaria brizantha* (Marandu grass)-*Arachis pintoi* (forage peanut cv. Belomonte) mixtures exceeded that on grass monocultures fertilized with 120 kg N/ha. Grass Forage Sci. 2020; 75:28–36. doi: https://doi.org/10.1111/gfs.12463

42. Mullenix MK, Sollenberger LE, Wallau MO, Rowland DL, Blount AR, Vendramini JMB, et al. Sward structure, light interception, and rhizome-root responses of rhizoma peanut cultivars and germplasm to grazing management. Crop Sci. 2016; 56:899–906. doi: http://dx.doi.org/10.2135/cropsci2015.08.0508

43. Burns JC, Sollenberger LE. Grazing behavior of ruminants and daily performance from warm-season grasses. Crop Sci. 2002; 42:873–881. https://doi.org/10.2135/cropsci2002.8730

44. Newman YC, Sollenberger LE, Kunkle WE, Chambliss CG. Canopy height and nitrogen supplementation effects on performance of heifers grazing limpograss. Agron J. 2002; 94:1375–1380. doi: https://doi.org/10.2134/agronj2002.1375

45. Lima JA, Pinto JC, Evangelista AR, Santana RAV. Amendoim Forrageiro (*Arachis pintoi* Krapov. & Gregory); 2003. Available from: http://livraria.editora.ufla.br/upload/boletim/extensao-tmp/boletim-extensao-001.pdf. (In Portuguese).

46. Andrade CMS, Wadt PGS, Zaninetti RA, Valentim JF. Recomendação de calagem e adubação para pastagens no Acre. Circ. Tec. 46. Embrapa. 2014. Available from: https://ainfo.cnptia.embrapa.br/digital/bitstream/item/115015/1/25378.pdf. (In Portuguese)

47. Dubeux JCB, Blount ARS, Mackowiak C, Santos ERS, Pereira Neto JD, Riveros U, et al. Biological N_2 fixation, belowground responses, and forage potential of rhizoma peanut cultivars. Crop Sci. 2017; 57:1027–1038. doi: http://dx.doi.org/10.2135/cropsci2016.09.0810

48. Carvalho LR, Pereira LET, Hungria M, Camargo PBD, Silva SC. Nodulation and biological nitrogen fixation (BNF) in forage peanut (*Arachis pintoi*) cv. Belmonte subjected to grazing regimes. Agric. Ecosyst. Envir. 2019; 278:96–106. doi: https://doi.org/10.1016/j.agee.2019.02.016

Fig1.

Click here to download Figure Fig1.tif.tif

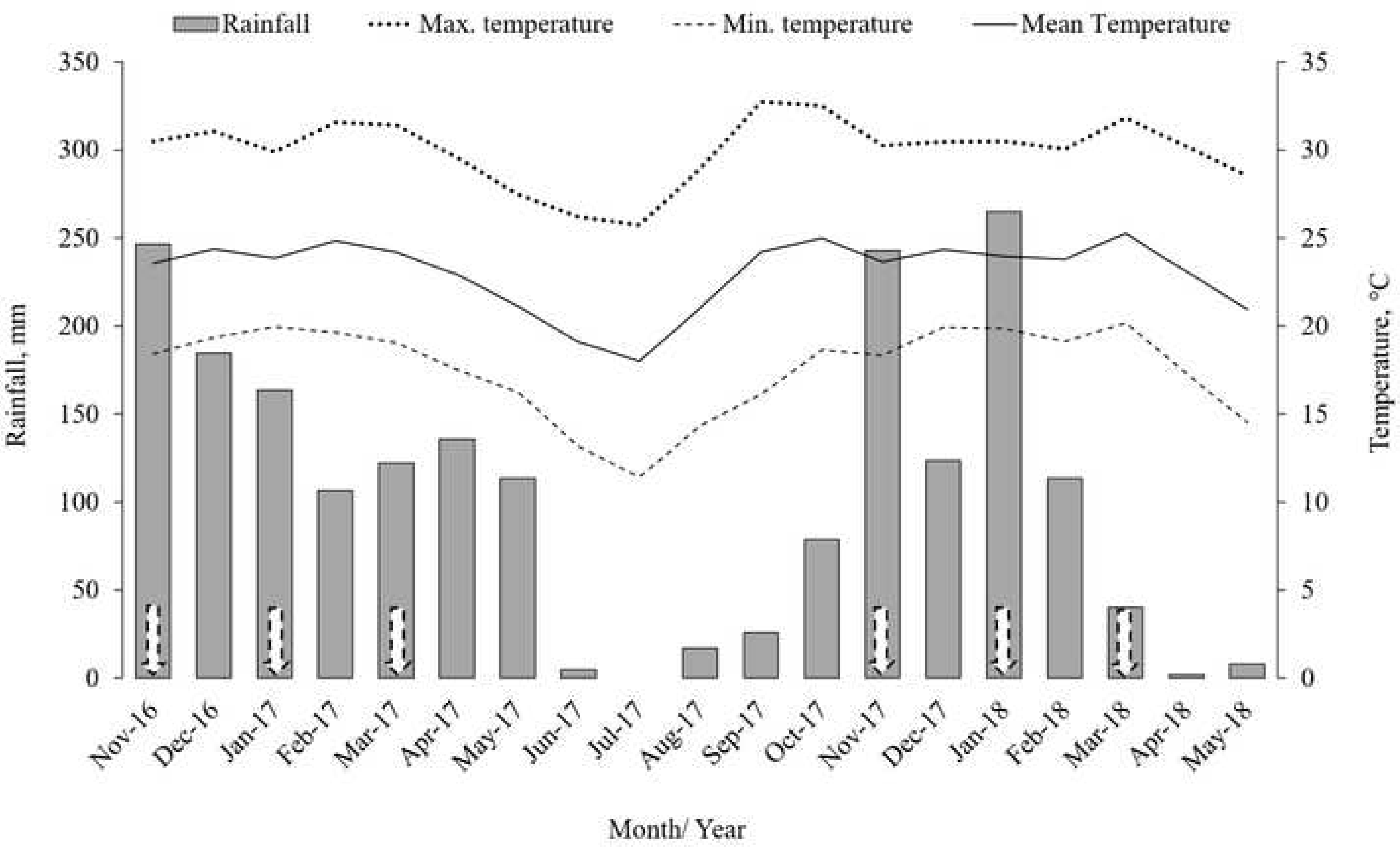

Fig2.

Click here to download Figure Fig2.tif.tif

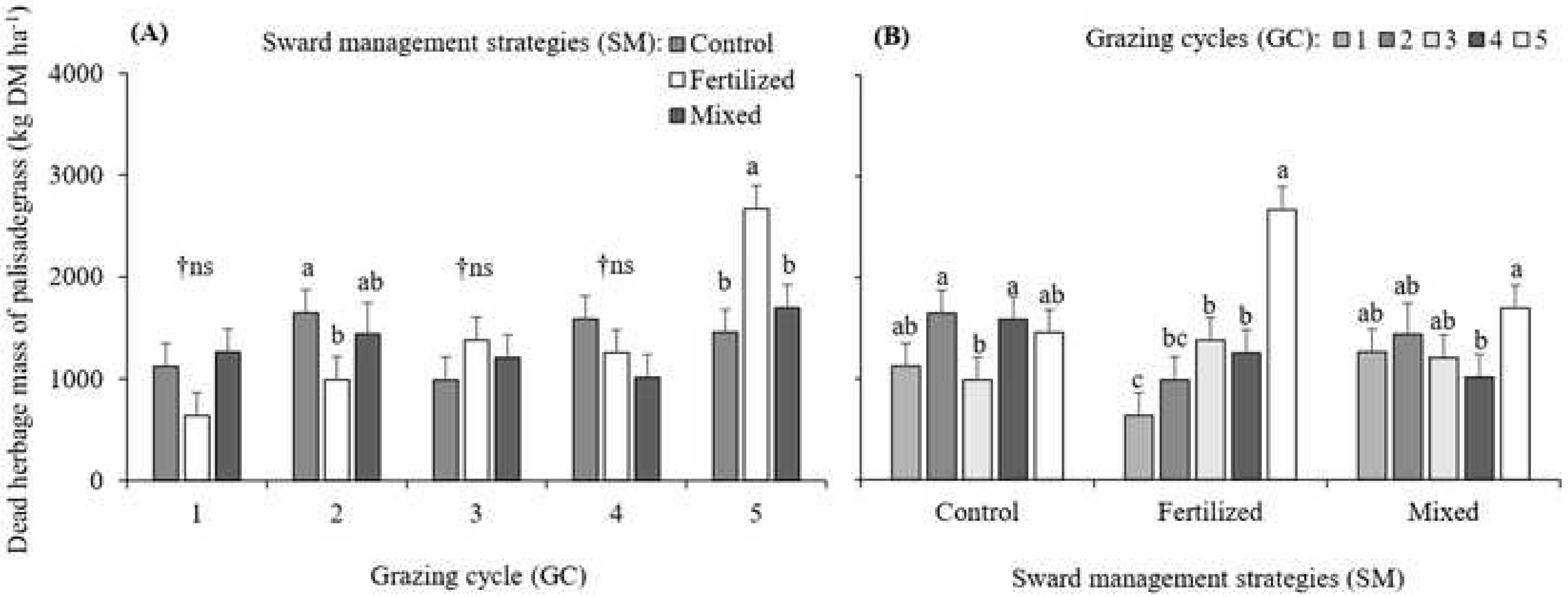

Fig3.

Click here to download Figure Fig3.tif.tif

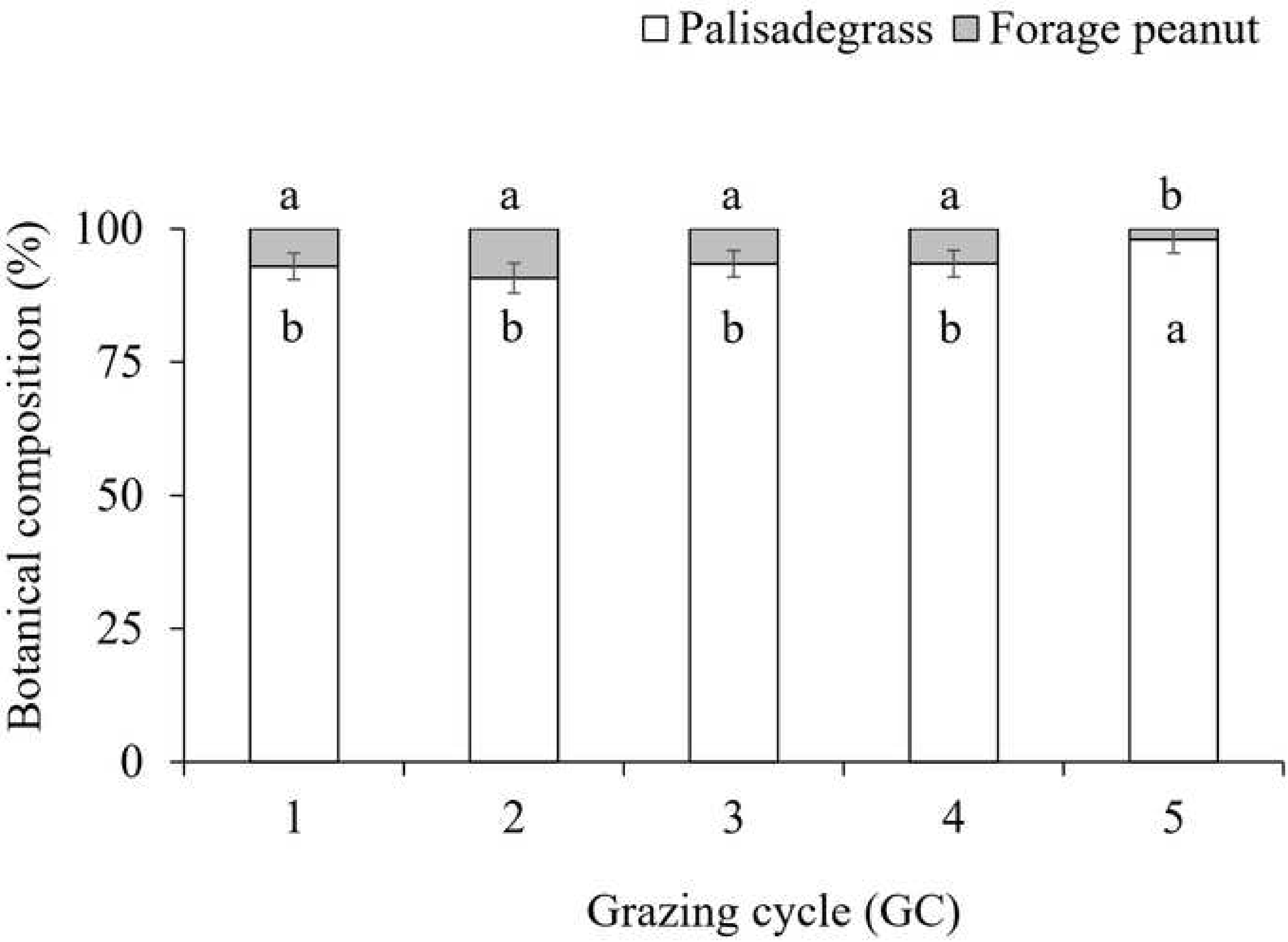

Fig4.

Click here to download Figure Fig4.tif.tif

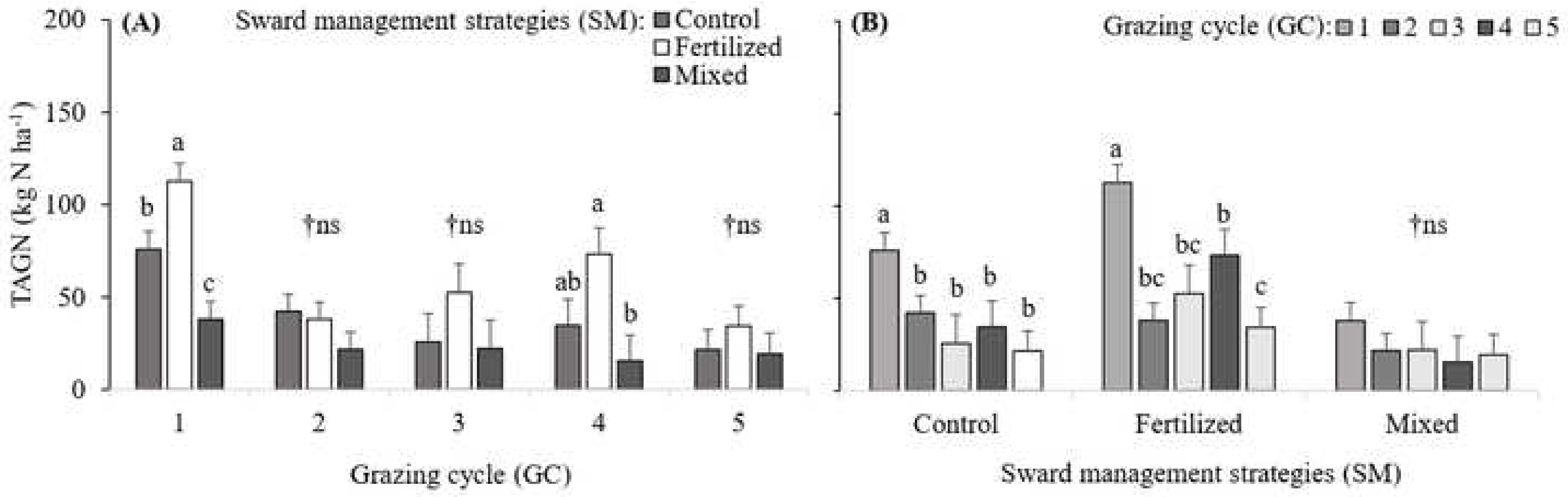

Supplement: Attachment — Submitted filename: PONE-D-20-17268_reviewer.pdf [file pone.0247931.s002.pdf]
