# Supplementary material for: Could forage peanut in low proportion replace N fertilizer in livestock systems?
Source: PLoS One. 2021 Mar 3;16(3):e0247931. doi: 10.1371/journal.pone.0247931 (PMC7928512; doi:10.1371/journal.pone.0247931)
Supplement: Attachment — Submitted filename: PONE-D-20-17268_R1_2021.pdf [file pone.0247931.s006.pdf]

# PLOS ONE

## Could forage peanut in low proportion replace N fertilizer in livestock systems?

--Manuscript Draft--

| | |
|---|---|
| **Manuscript Number:** | PONE-D-20-17268R1 |
| **Article Type:** | Research Article |
| **Full Title:** | Could forage peanut in low proportion replace N fertilizer in livestock systems? |
| **Short Title:** | Could forage peanut in low proportion replace N fertilizer in grassland? |
| **Corresponding Author:** | Vanessa Zirondi Longhini, Ph.D.<br>Universidade Estadual Paulista Julio de Mesquita Filho - Campus de Jaboticabal<br>Jaboticabal, BRAZIL |
| **Keywords:** | biological nitrogen fixation; grasses; legume; nitrogen fertilization; palisadegrass-forage peanut mixtures; pasture management; urea fertilizer |
| **Abstract:** | Palisadegrass [ *Urochloa brizantha* (Hochst. ex A. Rich.) R. D. Webster cv. Marandu] is widely used in Brazil and typically managed with little or no N fertilizer, leading to pasture decline. The current relationship between beef price and fertilizer cost in Brazil does not favor fertilizer use in pastures. Legume inclusion is an alternative to add N, but oftentimes legumes do not reach significant proportion (> 30%) in pasture botanical composition. This study evaluated herbage responses to N inputs and pasture species composition, under intermittent stocking. Treatments included palisadegrass-forage peanut ( *Arachis pintoi* Krapov. & W.C. Greg. cv Amarillo) mixture (mixed), unfertilized palisadegrass (control), and palisadegrass fertilized with 150 kg N $ha^{-1}$ $yr^{-1}$ (fertilized). Treatments were applied for two rainy seasons with five growth cycle (GC) evaluations. Response variables included herbage biomass, herbage accumulation, morphological components, total aboveground N of forage peanut ($TAGN_{FP}$), and contribution of biological $N_2$ fixation (BNF). Herbage biomass at pre-grazing was greater for fertilized palisadegrass [5850 kg dry matter (DM) $ha^{-1}$ ] than for the palisadegrass-forage peanut mixture (3940 kg DM $ha^{-1}$ ), while the unfertilized palisadegrass (4400 kg DM $ha^{-1}$ ) did not differ from the mixed pasture. Nitrogen fertilizer increased leaf mass of palisadegrass (2490 kg DM $ha^{-1}$ ) compared with the control and mixed treatments (1700 and 1310 kg DM $ha^{-1}$ , respectively). The percentage of N derived from the atmosphere (%Ndfa) for forage peanut ranged from 79 to 85% and BNF ranged from 0.5 to 5.5 kg N $ha^{-1}$ $cycle^{-1}$ . Overall, benefits from forage peanut were minimal because legume percentage was less than 10%. Beginning a grazing before 90% canopy light interception or imposing a shorter post-grazing stubble height may aid in reducing shading to the decumbent-growing forage peanut when planted with palisadegrass. |
| **Order of Authors:** | Vanessa Z. Longhini, M.D. |
| | Abmael S. Cardoso |
| | Andressa S. Berça |
| | Robert M. Boddey |
| | Ricardo A. Reis |
| | José C.B. Dubeux |
| | Ana C. Ruggieri |
| **Response to Reviewers:** | Jaboticabal, Sao Paulo State, Brazil, November 18th, 2020.<br><br>Dear Plos One Editor,<br><br>Subject: Submission of a revised manuscript PONE-D-20-17268 entitled “Could forage peanut in low proportion replace N fertilizer in livestock systems?”.<br><br>Thank you for considering our manuscript “Could forage peanut in low proportion replace N fertilizer in livestock systems?” for publication. We carefully addressed each of the reviewers’ comments, and we hope that the changes will meet your |

Powered by Editorial Manager® and ProduXion Manager® from Aries Systems Corporation

expectations. Our answers are listed below (in red) and a version of the revised manuscript with all the modifications (i.e., corrections and additional information inserted) highlighted in “track-change mode”. We would like to thank you and the reviewers for the excellent review, which certainly improved the manuscript quality. Thank you for the suggestions and possible appreciation of our work for publication in this renowned journal. The authors are available for other modifications that may improve the quality of the manuscript, as well as additional clarifications.
Sincerely,

Vanessa Zirondi Longhini, Ph.D.
Department of Animal Science
São Paulo State University (UNESP-FCAV)
Jaboticabal (SP), Zip 14884-900, Brazil

Comments to Author
Additional Editor Comments:
1. Thank you for submitting your manuscript to PLOS ONE. After careful consideration, we feel that it has merit but does not fully meet PLOS ONE’s publication criteria as it currently stands. Therefore, we invite you to submit a revised version of the manuscript that addresses the points raised during the review process. A major problem with the paper – as highlighted by both reviewers – is that the authors goal is to compare the legume-grass mixture with the grass monoculture, but they failed to achieve an adequate amount of legume in the mixture to provide a legitimate test of the mixture's potential. Thus, although they gathered a lot of interesting data, they did not adequately test their hypotheses because their legume-grass mixture had much too little legume to provide a valid test. It may be possible to develop an interesting study even though the pintoi did not perform as expected.
Authors Response: Many thanks for considering a new version of the manuscript. After careful revision, we changed the focus of the manuscript. Please, see the hypothesis in the introduction section: “We hypothesized that the forage peanut mixed with non-N fertilized grass would benefit the herbage responses through BNF, compared with unfertilized grass in a monoculture. However, the proportion of forage peanut in the sward in the present study was low. Thus, we decided to test whether there is a meaningful and measurable contribution of the N from forage peanut even at minimal participation and whether this contribution could be close to those systems using synthetic N fertilizer. Therefore, this study evaluated the performance of palisadegrass under three nitrogen management strategies (unfertilized, N fertilized, or mixed with the legume forage peanut), aiming to identify how different N inputs may affect grass responses”. We rewrote the results and discussion in separate sections.

2. As reviewers suggest, the use the weather and growth cycle data to find correlations (if they exist) and thus an explanation of results.
Authors Response: Thank you for this suggestion. We checked the correlations, and they were included in the manuscript.

3. There is also a substantial lack of organization, excess detail and insufficient attention to testing of hypotheses that they can achieve, given what the study actually tested. I agree with reviewer two that the results and discussion should be separated, and the need to reduce by about 50% including all redundant measurements, and figures that are redundant with tables. So this is a really major revision needed, in terms of reanalysis and rewriting - just so this is a clear recommendation and I hope that this is undertaken as the work is important, just challenging given that the original hypotheses were not possible to test due to establishment issues.
Authors Response: We fully agree with this critical and useful comments. Please, note that we split the results and discussion section, removed redundant measurements and figures, which was possible to reduce the manuscript.

Reviewers' comments to author
Reviewer 1
The paper is generally well written. In the attached pdf, there are comment boxes suggesting revisions or asking questions. The biggest issue the authors are dealing with is that the establishment of pintoi was not satisfactory. This impacts any conclusions about the three treatments, because you are comparing the two strong grass monocultures against a mixture that does not reflect the true potential of the

Powered by Editorial Manager® and ProduXion Manager® from Aries Systems Corporation

mixture. The authors try to get around this by saying in one case that they are describing what happens during the establishment period, but in another case they say they are addressing whether there is meaningful and measurable contribution from peanut even at a very low participation. I encourage them to focus the paper on one of these two approaches, not both of them. I think the latter is most plausible. The problem with saying they are looking at what happens during establishment is that the planting occurred in 2014 and the experiment did not start until 2016. Yes, I very well know that Arachis establishes slowly, but to suggest that Years 3 and 4 after planting are the establishment period is a rather large stretch. I suggest that instead they focus on whether there is value even of a small proportion of peanut in the association and do that intentionally and consistently throughout the paper so that the theme is well developed and serves as a point of focus.
Authors Response: Thank you very much for your effort in revising our manuscript. All suggestion made in the pdf file was included in the new version. We agree with you on all points raised during the revision process. We are now focusing on the small contribution of Arachis pintoi to the system instead of the legume establishment process. Please, note that some sentences and references were removed during the review process to reduce redundancy and be more textually efficient, as suggested by the editor and reviewer 2.

1.This is not true everywhere all the time, so I suggest "The current relationship between beef price and fertilizer in Brazil does not".
Authors Response: We appreciate this comment. The sentence was corrected according to the suggestion (line 21).

2.vegetation types implies species or growth habit, suggest using "sward management strategies" as you do later, or perhaps "pasture species composition".
Authors Response: Thank you for the suggestion. We changed to “pasture species composition” as suggested (line 24).

3.I prefer "season" because trial implies a different experiment, and you simply repeated the same experiment in two year, at least that is what I understand.
Authors Response: We agree with the reviewer. We changed to “season” as suggested (line 196).

4.In-stead of "Herbage structures", I suggest "Herbage and canopy characteristics".
Authors Response: Thank you for the suggestion. We have made changes as suggested (line 207).

5.I am not sure what you mean by this sentence. If height is not consistent with LI, then how can you use the same height for comparisons? Expand what you are trying to say here so that it is more clear. What do you mean by lower cost? Easier and cheaper to measure height than LI???
Authors Response: We agree with your comment. The sentence was confusing. After careful revision, we removed the sentence from the new version.

6.suggest "herbage accumulation rates in monoculture or mixed pastures". The word especially does not seem to fit when you are discussing more than one item.
Authors Response: The sentence was removed in the new version.

7.occurred due to drought conditions.
Authors Response: Thank you for the suggestion. We rephrased the sentence in the new version: “Nitrogen fertilization combined with favorable weather conditions promoted greater green biomass (leaf and stem) and reduced the senescent biomass, while the N fertilization and drought conditions of the transition period between rainy to dry season may have increased the senescent biomass”.

8.resulting in lesser green herbage mass (leaf and stem) and greater dead mass in GC5.
Authors Response: Thank you for the suggestion. We rephrase the sentence in the new version (Please, see the last answer).

9.interception threshold before the other treatments. This may have resulted in competition for light among tillers, and the plant elongates stem under these conditions

in search of light for the younger leaves.
Authors Response: Many thanks for your suggestion. We have made changes as suggested (lines 379 and 383-384).

10.season, tillers may have increased in number or weight [34].
Authors Response: Removed from the new version.

11.It isn't totally clear to me how you planted in 2014 and started the study in 2016, yet you describe the period of study as the establishment period. It seems that you simply had an inferior stand of peanut, and it was never going to get better.
Authors Response: You are correct. We are now focusing on the forage peanut contribution to the system, even at minimal participation. This sentence was removed from the new version.

12.Keep in mind that the mixed treatment had only 90% LI on average, right? So, you would need to reduce this number to considerably less than 90, would you not?
Authors Response: We are grateful for this comment. We agree with you. In the mixed system the grazing started at 90% LI, therefore, we changed to “less than 90% of light interception” as suggested (line 805).

Reviewer 2
The authors report on the response of palisade pasture with no N inputs, mineral N, or Pintoi peanut (after 2-year establishment phase). The legume did not benefit sward productivity, which was assumed to be due to inadequate establishment length and periodic dry periods across several grazing cycles. Negative results in research are useful if well-delivered and if it addresses important topics. Since mineral N replacement is of great interest in food production, this topic has value. However, there are some major challenges the authors need to address. Many were included in the attached Word document (see attachment).
Authors Response: Thank you very much for all the valuable advice and points raised during the revision process. We hope that all changes will meet your expectations. We included the suggestions made in both files.

Other notable points:
- Experimental design as a Completely Randomized Design. Is that the case? Typically, these experiments are Randomized, Complete Block Design. It might be beneficial to include a figure with the experimental design illustrated.
Authors Response: Yes, the experimental design was in a completely randomized design. We included a figure with the experimental design (Figure 2).

- The manuscript was exceptionally long and many of the measurements were variations of key measurements, which made several parts of the Results and Discussion section seem redundant. I think the Conclusions section exemplified the simplicity of the results and the authors should use that as a guide on how to address their results in the main body of the document. Biomass was measured, leaf biomass, stem biomass, green herbage, dead herbage (not sure how that is defined), and daily growth rates. The results were clear but the authors greatly complicate the results with long discussion on the each of these (and several other) measurements that all lead to the same conclusion (the pintoi did not contribute to productivity as much as N fertilizer). Not much of a mystery there! I suggest dropping much of the redundant measurements, figures that are redundant with tables and focus on the key points. The pintoi did not perform as expected and to use the weather and growth cycle data to find correlations (if they exist) and explanation of results. This manuscript can easily be reduce by 50% without impacting the message. Just because you measured lots of things does not mean they all need to be reported on! Pick the key points and focus on them. Keep the reader interested!
Authors Response: We agree with you. We removed some redundant data and separated the results from the discussion section to reduce the manuscript. We are now focusing on the small contribution of Arachis pintoi to the system instead of the legume establishment process. Correlations were tested and were included as an explanation of the results. The term “dead herbage” was changed to “senescent biomass” and was defined in the Materials and methods section as “senescent biomass (brown leaf and stem attached to the plant)”. We hope that all changes will meet your expectations.

Powered by Editorial Manager® and ProduXion Manager® from Aries Systems Corporation

- To help reduce redundancy and be more textually efficient, it may help to structure this manuscript with separate Results and Discussion sections rather than combining them as Results and Discussion. Then the Discussion section can flow freely without stepping through each measurement one by one and repeating the same cause-effect for every dependent variable.
Authors Response: Many thanks for your suggestion. Please, note that we separated the Results and Discussion section, in this way, the manuscript was reduced without changing the message.

- Paragraphs are not consistently indented (run-on paragraphs).
Authors Response: Thank you for catching it. We revised according to the pdf in the submission guidelines.

- Was not sure how the 3 N fertilizer split applications factored into grass response (growth cycles) in terms of biomass, as some cycles had greater biomass than others. Seems that would impact biomass differently over the season.
Authors Response: We split the application to reduce possible N losses through volatilization, leaching, or runoff. However, some losses may have occurred in the first growth cycles due to high rainfall after urea application. However, the biomass in the fertilizer treatment was greater than in the mixed and control treatment. To improve the results visualization, we change the table for the figure (Fig 3). Please note that the fertilized treatment had the same pattern across all growth cycles.

- Make sure any abbreviations used in tables and figures are written out (in title, footnote, caption, as appropriate).
Authors Response: Checked.

1.Do not understand the notations, in terms of the different years for each grazing evaluation. Might be easier to follow if presented as a table, with a column for each grazing season.
Authors Response: Many thanks for your suggestion. We included the date information in Table 1.

2.This paragraph is probably better suited for the Discussion section.
Authors Response: We appreciate this suggestion. We skip the sentence to the Discussion section (lines 422-424).

3.Include the year.
Authors Response: We included the year in the revised manuscript.

4.Were the GC equally separated in time? If not, then repeated measures cannot be used. Difficult to determine by the way the sampling dates are presented. The last sampling date seems the most suspicious.
Authors Response: The growth cycle was not equally separated because we used the intermittent stocking method, i.e., GC with irregular intervals (Allen et al., 2011). The rest period was interrupted only when the first treatment was achieved at 95% of light interception. We included Table 1 with the date of the growth cycle. Therefore, with your affirmation, we reanalyzed the statistics removing the repeated measures.

5.Why is forage peanut addressed separately? It seems that treatment (regardless of which one) is addressed as a single factor. Not sure I understand why you separate the different treatments (grass ANOVA vs peanut ANOVA).
Authors Response: We separated the forage peanut results from the mixed treatment to evaluate the palisadegrass performance among nitrogen management strategies and the forage peanut patterns among the growth cycle. Thus, forage peanut was statistically compared among GC (fixed effect) without the other treatments.

6.You should not imply that there were significant differences when there was not by talking in terms of percent differences. The PHM was not different between control and fertilized grass.
Authors Response: Corrected according to the new statistical analyses ($P \leq 0.1$). Please, see lines 373-376.

7.If you had legume growth in the mixture but the sward yield was unchanged, then it

must have reduced the grass growth! What you might say instead is that inclusion of the legume did not impact sward productivity compared to grass monoculture.
Authors Response: Removed from the new version.
8.Abbreviations need to be defined.
Authors Response: Revised.

9.If true then that would be a SM x GC interaction, which you did not have. All you can say is that there was a GC effect and explain what it was. GC response was independent of SM.
Authors Response: Revised according to the new statistical analyses ($P \leq 0.1$). Please, see lines 285-290.

10.Unclear how this statement explains the reported response. Either explain more thoroughly or omit the sentence.
Authors Response: The sentence was removed.

11.I am certain there are water response literature available to support your supposition about Arachis pintoi response to drought or dry soils. We know this to be the case in the US.
Authors Response: Thank you for this suggestion. We included two references to support this supposition: "In Northwest Brazil, [17] reported that the drought stress reduced the forage peanut biomass in the mixed system during the dry season. On the other hand, in Northeast Brazil, [46] did not find a negative impact of the dry season on the forage peanut growth. The authors suggested positive legume performance due to the favorable climatic weather in the study region, even during the dry season. Thus, differences among Brazilian regions, weather conditions, and latitude play an important role in the forage peanut growth, mainly in the dry season" (lines 411-417).

12.I think this is more a factor of large variability in your data set. Since the yields were functions of the growth rate and they were significantly different, then it would reason that the growth rate would also differ. You have SM x GC interaction (p=0.1) for HAR. For field studies, sometimes using 0.1 as your limit is acceptable.
Authors Response: Many thanks for your suggestion. We are now considering the $P \leq 0.1$ for all variables, including the correlations. Please, see lines 285-292.

13.Not easy to see how Fig 1 (given in dates) relates to the growth response (given as cycle numbers). You should consider how to compare them to better support your argument.
Authors Response: Thank you. We changed the figure grouping the date by growth cycle to support the data. We also performed a correlation between rainfall and growth cycle to explain the results.

14.What time of year. It's a real challenge to keep scrolling back to determine when each cycle was compared to the season/date.
Authors Response: The greater L:S ratio occurred in the first growth cycle (November 2016 and December 2017) at the onset of the rainy season in Brazil. The growth cycle dates were detailed in Table 1.

15.Basically, the extra N contributed to greater stem growth. That seems reasonable. It seems that extra N would either have no effect or decrease L:S. As part of a sward, there is no benefit for a plant to creates relatively more leaf mass, when it cannot intercept anymore light than what it already has. A quick check, led to this example. I'm sure you can find more relevant references, as well: Grass and Forage Science 54(3):255 – 266 DOI: 10.1046/j.1365-2494.1999.00178.x
Authors Response: Thank you for this suggestion. More references was included about the extra N: "The extra N may have contributed to competition for light among tillers, and the plant elongates the stem under these conditions in search of the light for the younger leaves [30]. As part of a canopy sward, there is no benefit for a plant to create relatively more leaf biomass when it cannot intercept anymore light than it already has [38], which may explain the greatest stem proportion and canopy height in the N fertilized treatment without effect on the L:S ratio among N management strategies. In the present study, the rainfall was positively correlated with canopy height ($r = 0.23$, $P = 0.013$), leaf biomass ($r = 0.31$, $P < 0.001$), and L:S ratio ($r = 0.17$, $P = 0.064$) of palisadegrass. Stem elongation is not desirable for grazing animal nutrition, basically

Powered by Editorial Manager® and ProduXion Manager® from Aries Systems Corporation

for three reasons. First, increasing the stem can reduce the leaf proportion [39]; second, the stem elongation increases the canopy height, which will affect the ingestive animal behavior, such as reducing the number of tongue sweeps, bite volume, and bite weight [39, 40]; and third, the stem acts as a support structure of leaves, which has secondary cell walls, such as lignin, which are undigestible components [41, 42]. These factors can limit the selection of leaf and, consequently, reduces animal gain [43]" (lines 383-396).

16.Why is leaf:stem discussed under "herbage structures" instead of morphological components?
Authors Response: Thank you for catching it. We included the leaf:stem on the morphological components.

17.Leaf:stem ratio is derived from these measurements. Therefore, leaf:stem ratio should be placed in this section.
Authors Response: You are correct. We included the L:S ratio in the morphological components.

18.I do not understand this argument. I suspect that initiating grazing at 95% light interception was not well maintained rather than canopy heights differing at exactly 95% light interception. I do not think you could have managed the systems that accurately, based upon the information provided. An alternative interpretation is does this mean that canopy height was measured at less than 95% light interception for the unfertilized grass treatment? My interpretation of the data is that the low N treatments (grass or mixture) did not grow as much over the same amount of time as the N fertilized grass. Shows up in biomass and related measurements. No surprise…
Authors Response: Thank you for the critical and useful comments. After careful revision, we removed the sentence and included the suggestion (line 380-382).

19.Abbreviations need to be written out the first time they are used in the Materials and Methods or Results and Discussion section, regardless if they were used in the Introduction.
Authors Response: We appreciate this comment. We checked the abbreviations in the whole manuscript.

20.You did not have grazing frequency comparisons, but rather repeated grazing (cycles). There is nothing presented that suggests that you were testing different frequencies.
Authors Response: We appreciate this comment. We removed the sentence to fix it.

21.This is a key point and brings to question why this study was attempted if the peanut was not established. The message seems to be that in terms of yield, the peanut provides no benefit. IF you had addressed forage quality, animal gain, etc., then perhaps you would have found benefit of the mixed sward. How many years does it take to declare the peanut fully established in a grass sward?
Authors Response: Many thanks for this useful comment. We included a sentence to bring this key point: "Pereira [46] reported that 20.5 to 23.8% of forage peanut mixed with palisadegrass resulted in a liveweight gain per ha 29% greater than in N fertilized system (dose of 120 kg N ha-1). Therefore, if our study had addressed nutritive value and animal performance, perhaps we would have found the mixed system benefits even at minimal legume participation" (lines 427-431). Based on Tamele et al. (2018), in similar environmental conditions of this study, after three years of establishment with the canopy height of palisadegrass at 20 cm, it was possible to guarantee a successful forage peanut establishment with 19 to 38% of the legume in the botanical composition. However, we are now focusing on the small contribution of Arachis pintoi to the system instead of the legume establishment process.

Tamele OH, Sá OAAL, Bernardes TF, Lara MAS, Casagrande DR. Optimal defoliation management of brachiaria grass–forage peanut for balanced pasture establishment. Grass Forage Sci. 2018; 73:522–531. doi: http://dx.doi.org/10.1111/gfs.12332

22.This is much greater than your reported values, so what can the reader conclude about your system?
Authors Response: You are right. However, we rewrote the sentence to support the

last answer (Please, see lines 427-429).

23.Maybe true for grasses, particularly those that lose quality, with time between grazing events, but do you expect to the same extent (or at all) with the peanut species? I doubt it. You can use this argument for the grass discussion section.
Authors Response: We appreciate this comment, you are correct. We moved the sentence to the grass discussion section (lines 392-393 and 395-396).

24.Across all conditions?
Authors Response: Many thanks for your comment. Please, note that we included the sentence: “Under regular weather conditions (rainfall, temperature, and photoperiod), warm-season C4 grasses are typically more competitive than legumes, shifting the balance in mixed swards towards the grass [14]” (lines 405-407).
25.Rhizoma peanut is not “seeded”, thus the name “rhizoma” peanut (Arachis glabrata). It is sprigged.
Authors Response: Thank you for catching it. However, the sentence was removed in the revision.

26.I do not understand what you are trying to say (in the dung?). You have the biomass values. You can simply compare proportions based on biomass.
Authors Response: We were trying to say that although the legume proportion in the botanical composition was low, the animals selected the forage peanut to compose their diet, and this affirmation we could perform by the isotopic 13C in the dung, which was numerically greater than in the botanical composition. However, we removed the sentence to avoid confusion.

27.Are the last two sentences addressing Reference 45 or your study? This is unclear and the discussion seems to go off-track.
Authors Response: The sentence was about our study. This was about the forage peanut selection by grazing animals. As we said in the last question, we were trying to say that although the legume proportion in the botanical composition was low, the animals selected the forage peanut to compose their diet. We could perform this affirmation by the isotopic 13C in the dung, which was numerically greater than in the botanical composition (but we did not perform the statistical analyses). Taking into account the digestibility of forage peanut, probably the legume in heifers’ diet was greater than in the botanical composition, which occurred by the animal selection. However, we removed the sentence to avoid confusion.

28.Since the proportions don’t change (except C5), you can say it in the text and no need for Fig. 3.
Authors Response: We are grateful for this comment. We removed the Fig. 3 and wrote the information in the results section: “The botanical composition of forage peanut and palisadegrass in the mixed treatment differed among GC ($P < 0.001$), however, the proportions did not change, except in GC5, which had the lowest forage peanut proportion (2% of the botanical composition). The proportion of forage peanut among GC1 to GC4, ranged between 7 to 9% of the botanical composition” (lines 343-347).

29.This sentence does not sound correct. What do you mean?
Authors Response: Thank you for this comment. It is about the amount of herbage mass aboveground, which will affect the biological N2 fixation. We removed the sentence in the new manuscript version.

30.How many stages are there and what is the duration of these stages?
Authors Response: Thank you for this comment. In Alencar et al. (2018), the evaluations occurred only at the end of each experimental period, i.e., it was seven and eight months after seeding. However, the sentence was removed from the new manuscript version.

Alencar NM, Vendramini JMB, Santos AC, Silveira ML, Dubeux JCB, Sousa LF, et al. Herbage characteristics of pintoi peanut and palisadegrass established as monoculture or mixed swards. Crop Sci. 2018; 58:1–7. doi: http://dx.doi.org/10.2135/cropsci2017.09.0538

| Additional Information: | |
|---|---|
| **Question** | **Response** |
| **Financial Disclosure**<br><br>Enter a financial disclosure statement that describes the sources of funding for the work included in this submission. Review the submission guidelines for detailed requirements. View published research articles from *PLOS ONE* for specific examples.<br><br>This statement is required for submission and **will appear in the published article** if the submission is accepted. Please make sure it is accurate.<br><br>**Unfunded studies**<br>Enter: *The author(s) received no specific funding for this work.*<br><br>**Funded studies**<br>Enter a statement with the following details:<br>• Initials of the authors who received each award<br>• Grant numbers awarded to each author<br>• The full name of each funder<br>• URL of each funder website<br>• Did the sponsors or funders play any role in the study design, data collection and analysis, decision to publish, or preparation of the manuscript?<br>• **NO** - Include this sentence at the end of your statement: *The funders had no role in study design, data collection and analysis, decision to publish, or preparation of the manuscript.*<br>• **YES** - Specify the role(s) played.<br><br>* typeset | The authors were supported by São Paulo Research Foundation – FAPESP (VZL: grant # 2016/11086-1; ASC: grant# 2017/11274-5, and RAR: thematic grant # 2015/16631-5, http://www.fapesp.br/) and by Cientista de Nosso Estado for author RMB from the Rio de Janeiro State Research Foundation – FAPERJ (http://www.faperj.br/). This work was funded by National Council for Scientific and Technological Development – CNPq (RMB: grant # 404169/2013-9, http://www.cnpq.br/). The funders had no role in study design, data collection and analysis, decision to publish, or preparation of the manuscript. |
| **Competing Interests**<br><br>Use the instructions below to enter a competing interest statement for this submission. On behalf of all authors, disclose any competing interests that could be perceived to bias this work—acknowledging all financial support and any other relevant financial or non-financial competing interests. | The authors have declared that no competing interests exist. |

*Powered by Editorial Manager® and ProduXion Manager® from Aries Systems Corporation*

| | |
|---|---|
| This statement **will appear in the published article** if the submission is accepted. Please make sure it is accurate. View published research articles from *PLOS ONE* for specific examples.<br><br>**NO authors have competing interests**<br><br>Enter: *The authors have declared that no competing interests exist.*<br><br>**Authors with competing interests**<br><br>Enter competing interest details beginning with this statement:<br><br>*I have read the journal's policy and the authors of this manuscript have the following competing interests: [insert competing interests here]*<br><br>* typeset | |
| **Ethics Statement**<br><br>Enter an ethics statement for this submission. This statement is required if the study involved:<br><br>• Human participants<br>• Human specimens or tissue<br>• Vertebrate animals or cephalopods<br>• Vertebrate embryos or tissues<br>• Field research<br><br>Write "N/A" if the submission does not require an ethics statement.<br><br>General guidance is provided below. Consult the submission guidelines for detailed instructions. **Make sure that all information entered here is included in the Methods section of the manuscript.** | The procedures involving animals were approved by the Institutional Animal Care and Use Committee at São Paulo State University, SP, Brazil (No. 10356/14-CEUA). |

| | |
|---|---|
| **Format for specific study types**<br><br>**Human Subject Research (involving human participants and/or tissue)**<br>• Give the name of the institutional review board or ethics committee that approved the study<br>• Include the approval number and/or a statement indicating approval of this research<br>• Indicate the form of consent obtained (written/oral) or the reason that consent was not obtained (e.g. the data were analyzed anonymously)<br><br>**Animal Research (involving vertebrate animals, embryos or tissues)**<br>• Provide the name of the Institutional Animal Care and Use Committee (IACUC) or other relevant ethics board that reviewed the study protocol, and indicate whether they approved this research or granted a formal waiver of ethical approval<br>• Include an approval number if one was obtained<br>• If the study involved *non-human primates*, add *additional details* about animal welfare and steps taken to ameliorate suffering<br>• If anesthesia, euthanasia, or any kind of animal sacrifice is part of the study, include briefly which substances and/or methods were applied<br><br>**Field Research**<br><br>Include the following details if this study involves the collection of plant, animal, or other materials from a natural setting:<br>• Field permit number<br>• Name of the institution or relevant body that granted permission | |
| **Data Availability**<br><br>Authors are required to make all data underlying the findings described fully available, without restriction, and from the time of publication. PLOS allows rare exceptions to address legal and ethical concerns. See the PLOS Data Policy and FAQ for detailed information. | Yes - all data are fully available without restriction |

*Powered by Editorial Manager® and ProduXion Manager® from Aries Systems Corporation*

| | |
|---|---|
| A Data Availability Statement describing where the data can be found is required at submission. Your answers to this question constitute the Data Availability Statement and **will be published in the article**, if accepted.<br><br>**Important:** Stating 'data available on request from the author' is not sufficient. If your data are only available upon request, select 'No' for the first question and explain your exceptional situation in the text box.<br><br>Do the authors confirm that all data underlying the findings described in their manuscript are fully available without restriction? | |
| **Describe where the data may be found in full sentences. If you are copying our sample text, replace any instances of XXX with the appropriate details.**<br><br>• If the data are **held or will be held in a public repository**, include URLs, accession numbers or DOIs. If this information will only be available after acceptance, indicate this by ticking the box below. For example: *All XXX files are available from the XXX database (accession number(s) XXX, XXX.).*<br>• If the data are all contained **within the manuscript and/or Supporting Information files**, enter the following: *All relevant data are within the manuscript and its Supporting Information files.*<br>• If neither of these applies but you are able to provide **details of access elsewhere**, with or without limitations, please do so. For example:<br><br>*Data cannot be shared publicly because of [XXX]. Data are available from the XXX Institutional Data Access / Ethics Committee (contact via XXX) for researchers who meet the criteria for access to confidential data.*<br><br>*The data underlying the results presented in the study are available from (include the name of the third party* | Data will be available after acceptance. |

| | |
|---|---|
| *and contact information or URL).*<br>• This text is appropriate if the data are owned by a third party and authors do not have permission to share the data.<br><br>* typeset | |
| Additional data availability information: | |

Powered by Editorial Manager® and ProduXion Manager® from Aries Systems Corporation

Manuscript

Click here to access/download;Manuscript;Manuscript.docx



Could forage peanut in low proportion replace N fertilizer in livestock systems?

Vanessa Z. Longhini[1*], Abmael S. Cardoso[1], Andressa S. Berça[1], Robert M. Boddey[2], Ricardo A. Reis[1], José C.B. Dubeux[3], Jr., Ana C. Ruggieri[1]

[1] Department of Animal Science, São Paulo State University, via de acesso Professor Paulo Donato Castellane, Jaboticabal, SP, 14884-900, Brazil

[2] Embrapa Agrobiologia, Antiga Rodovia Rio-São Paulo, km 47. Seropédica, RJ, 23891-000, Brazil

[3] University of Florida, North Florida Research and Education Center, Marianna, FL, 32446, United States

* Corresponding author

E-mail: vanessa.longhini@unesp.br (VZL)

# Abstract

Palisadegrass [*Urochloa brizantha* (Hochst. ex A. Rich.) R. D. Webster cv. Marandu] is widely used in Brazil and typically managed with little or no N fertilizer, ~~leading~~ to pasture decline. The current relationship between beef price and fertilizer cost in Brazil does not favor fertilizer use in pastures. Legume inclusion is an alternative to add N, but oftentimes legumes do not reach significant proportion (> 30%) in pasture botanical composition. This study evaluated herbage responses to N inputs and pasture species composition, under intermittent stocking. Treatments included palisadegrass-forage peanut (*Arachis pintoi* Krapov. & W.C. Greg. cv Amarillo) mixture (mixed), unfertilized palisadegrass (control), and palisadegrass

fertilized with 150 kg N ha$^{-1}$ yr$^{-1}$ (fertilized). Treatments were applied for two rainy seasons with five growth cycle (GC) evaluations. Response variables included herbage biomass, herbage accumulation, morphological components, total aboveground N of forage peanut ($TAGN_{FP}$), and contribution of biological $N_2$ fixation (BNF). Herbage biomass at pre-grazing was greater for fertilized palisadegrass [5850 kg dry matter (DM) ha$^{-1}$] than for the palisadegrass-forage peanut mixture (3940 kg DM ha$^{-1}$), while the unfertilized palisadegrass (4400 kg DM ha$^{-1}$) did not differ from the mixed pasture. Nitrogen fertilizer increased leaf mass of palisadegrass (2490 kg DM ha$^{-1}$) compared with the control and mixed treatments (1700 and 1310 kg DM ha$^{-1}$, respectively). The percentage of N derived from the atmosphere (%Ndfa) for forage peanut ranged from 79 to 85% and BNF ranged from 0.5 to 5.5 kg N ha$^{-1}$ cycle$^{-1}$. Overall, benefits from forage peanut were minimal because legume percentage was less than 10%. Beginning a grazing before 90% canopy light interception or imposing a shorter post-grazing stubble height may aid in reducing shading to the decumbent-growing forage peanut when planted with palisadegrass.

# Introduction

In Brazil, grasses of the genus *Urochloa* cover an estimated 80 million ha. Marandu palisadegrass [*Urochloa brizantha* (Hochst. ex A. Rich.) R. D. Webster cv. syn. *Brachiaria brizantha* (Hochst. ex A. Rich.) Stapf.] the most used palisadegrass cultivar, is estimated to cover 56% of this area [1]. Low-input systems prevail in Brazilian livestock operations with minimal applications of limestone, gypsum, and N fertilizers. The combination of low soil fertility, lack of N fertilizer application, and overgrazing results in pasture degradation or pasture decline [2].

Nitrogen is a key element to improve pasture productivity [3] and many warm-season $C_4$ grasses respond positively to N fertilization by increased herbage accumulation and improved crude protein [4]. However, this response can vary with the environment, species, and management practices. Herbage accumulation of palisadegrass increased with increasing N fertilizer applications from 90 to 270 kg $ha^{-1}$, but there was a diminished increase in herbage accumulation per unit of N applied [4]. In this case, tripling the amount of N applied increased herbage accumulation by only 18% and increased potential for negative environmental impacts. Nitrogen fertilizer is manufactured using fossil fuel, which results in a significant carbon footprint [5]. Furthermore, N fertilizer may be lost as nitric oxide (NO), nitrogen dioxide ($NO_2$), nitrous oxide ($N_2O$), di-nitrogen ($N_2$), ammonia ($NH_3$), or via nitrate ($NO_3^-$) leaching [6, 7]. Several of these processes contribute to climate change (e.g., $N_2O$ emission) or increase $NO_3^-$ concentrations in surface water bodies and groundwater.

Integrating legumes into grazing systems is an alternative to mineral N fertilizer applications that may reduce negative environmental impacts [8]. Legumes add N to the system via symbiotic biological $N_2$ fixation (BNF) between legume and rhizobium bacteria in root nodules [9]. Legumes can also reduce production costs compared with N fertilizer [8, 10].

The use of grass-legume mixed pastures in tropical regions is less commonplace than in temperate regions. In North America, mixing grasses and legumes is a more common practice [11], that requires further study [12, 13]. In Brazil, few studies have evaluated grass-legume mixtures under grazing. Challenges to legume use have been attributed to poor establishment and persistence compared with grasses [10, 14, 15]. Forage peanut (*Arachis pintoi* Krapov. & W.C. Greg. cv. Amarillo) is a legume that has good potential for mixtures in Brazil because it persists well when mixed with fast-growing grasses such as *Urochloa* [16, 17]. A stoloniferous and prostrate growth habit makes the forage peanut ideal for grazing

because its meristems are protected from grazing animals [18]. Under tropical conditions, Tamele [15] reported that palisadegrass managed at a 20-cm canopy height improved forage peanut establishment in mixed systems. However, further research is necessary to determine if N from forage peanut grown in mixture with a grass is able to substitute for synthetic N fertilizer. The proportion of the legume in the mixture is important to determine the benefits of the association. According to Thomas [19], proportions between 20 - 45% in the herbage biomass was considered sufficient to supply N to the agroecosystem. However, not much has been done in terms of determining at which level the legume becomes important, or even if, at low participation, the legume is still able to benefit the overall system.

We hypothesized that the forage peanut mixed with non-N fertilized grass would benefit the herbage responses through BNF, compared with unfertilized grass in a monoculture. However, the proportion of forage peanut in the sward in the present study was low. Thus, we decided to test whether there is a meaningful and measurable contribution of the N from forage peanut even at minimal participation and whether this contribution could be close to those systems using synthetic N fertilizer. Therefore, this study evaluated the performance of palisadegrass under three nitrogen management strategies (unfertilized, N fertilized, or mixed with the legume forage peanut), aiming to identify how different N inputs may affect grass responses.

# Materials and methods

## Ethical approval

The procedures involving animals were approved by the Institutional Animal Care and Use Committee at São Paulo State University, SP, Brazil (No. 10356/14-CEUA).

# Experimental site

This study was conducted at Sao Paulo State University, Jaboticabal, Sao Paulo, Brazil (21º14’S and 48º17’W; 586 m asl) during two rainy seasons (November 2016 to March 2017 and December 2017 to April 2018). The soil at the experimental site is classified as an Oxisol [20]. Soil samples (0- to 20-cm depth) collected in 2014, 2017, and 2018 indicated the following means: pH ($CaCl_2$) 5.3; organic matter 32.4 g kg$^{-1}$; cation exchange capacity 74.8 mmolc dm$^{-3}$; P (ion-exchange resin extraction method) 10.9 mg dm$^{-3}$; Meh[illegible]h$^{-1}$ extractable Ca 28.3 mmolc dm$^{-3}$; Mehlich$^{-1}$ extractable Mg 9.7 mmolc dm$^{-3}$; Mehlich$^{-1}$ extractable K 4.2 mmolc dm$^{-3}$; base saturation 561 g kg$^{-1}$~~, respectively~~ [21]. Soil texture was 340 g kg$^{-1}$ sand, 140 g kg$^{-1}$ silt and 520 g kg$^{-1}$ clay [22]. The annual rainfall in this area is 1314 mm and mean annual air temperature is 22.7 °C (2010-2018). Rainfall and ~~mean~~ temperature data from the experimental site were extracted from a dataset belonging to the Agrometeorology section of the Department of Exact Sciences (Fig 1).

**Fig 1. Cumulative rainfall and mean air temperatures (maximum, mean, and minimum) for five growth cycles (GC) in Jaboticabal, Brazil.**

# Treatments and experimental design

There were three treatments (nitrogen management strategies, NM) with four replications, arranged in a completely randomized design with plot size of 20 × 60 m (Fig 2). Treatments were Control: Marandu palisadegrass in a monoculture, unfertilized (without N fertilizer or legume); Fertilized: Marandu palisadegrass fertilized with 150 kg N ha$^{-1}$ year$^{-1}$

(urea), split into three equal applications; and Mixed: Marandu palisadegrass mixed with forage peanut cv. Amarillo and no N fertilizer. These treatments were evaluated for two rainy seasons, with five growth cycles (GC) within each season (Table 1). Nitrogen was applied to the fertilized treatment in November, January, and March ~~for~~ each rainy season (50 kg N ha$^{-1}$ per application).

**Fig 2. Schematic representation of the experimental design. Three treatments with four replications, arranged in a completely randomized design. Experimental unit of 20 × 60 m (1200 m$^2$).** Control: Marandu palisadegrass in a monoculture (without N fertilizer or legume); Fertilized: Marandu palisadegrass fertilized with 150 kg N ha$^{-1}$ year$^{-1}$ (urea split into three equal applications); and Mixed: Marandu palisadegrass mixed with forage peanut cv. Amarillo and no N fertilizer. Intermittent stocking method with grazing periods lasting 3 d, 1 d for each paddock. Palisadegrass pasture was used as a reserve area to ~~keep~~ the animals during the rest periods.

**Table 1.** **Date of experimental period during five growth cycles and for two growing seasons.**

| Growth cycle (GC) | Initial date | Final date | Rest period [a] days |
|---|---|---|---|
| | **Growing season (2016/2017)** | | |
| Staging grazing | 07 Sept 2016 | 18 Sept 2016 | - |
| GC1 | 19 Nov 2016 | 30 Nov 2016 | 62 |
| GC2 | 10 Dec 2016 | 21 Dec 2016 | 21 |
| GC3 | 09 Jan 2017 | 20 Jan 2017 | 30 |
| GC4 | 06 Feb 2017 | 17 Feb 2017 | 28 |
| GC5 | 13 Mar 2017 | 24 Mar 2017 | 35 |
| | **Growing season (2017/2018)** | | |
| Staging grazing | 07 Sept 2017 | 18 Sept 2017 | - |
| GC1 | 04 Dec 2017 | 15 Dec 2017 | 77 |
| GC2 | 03 Jan 2018 | 14 Jan 2018 | 30 |
| GC3 | 05 Feb 2018 | 16 Feb 2018 | 33 |
| GC4 | 12 Mar 2018 | 23 Mar 2018 | 35 |
| GC5 | 23 Apr 2018 | 04 May 2018 | 42 |

[a] The rest period was interrupted, and grazing for all treatments initiated when one of the treatments reached 95% light interception.

# Plot establishment

In March 2014, existent 'Tifton 85' bermudagrass (*Cynodon* spp.) was sprayed with 2880 g a.i. ha$^{-1}$ of glyphosate [isopropylamine salt of N- (phosphonomethyl) glycine] in order to eradicate the existing vegetation. In October 2014, glyphosate plus 402 g a.i. ha$^{-1}$ of 2,4-D amine (2,4-dichlorophenoxyacetic acid) were applied, and subsequently, a crusher and chopper (Tritton, model 1800) was used to remove dead residue. Grass monoculture was seeded subsequently with a row spacing of 0.45 m and a seeding rate of 10 kg ha$^{-1}$ of pure live seed using a no-till seed drill (Semeato, model SHM 11-13). In the mixed treatment, forage peanut was simultaneously sown between rows of palisadegrass at a seeding rate of 8 kg ha$^{-1}$ of pure

live seed using a seed drill (Jumil, model 2640 PD Exacta). After seeding, all paddocks received 60 kg P, 20 kg S, 50 g Mo, 1 kg Cu and 1 kg B ha$^{-1}$, following recommendations of Werner [23]. At the beginning of the grazing trial in both years, single superphosphate (40 kg P ha$^{-1}$) and potassium chloride (50 kg K ha$^{-1}$) were applied. The grass-legume mixed pastures were not fertilized with N.

Before seeding in 2014, the three treatments were randomized and allocated into 12 experimental units (1200 m$^2$ each). Each experimental unit was split into three paddocks of 400 m$^2$.

## Grazing management

In September of each year, heifers were used to stage the plots. The grazing method was intermittent stocking with grazing periods lasting 3 d with 1 d for each paddock (Fig 2). The mob stocking technique was used for pasture defoliation [24] with a minimum of five crossbred dairy heifers (311 ± 6 and 234 ± 4 kg, 2016/2017 and 2017/2018, respectively) for grazing. Additional heifers were 'put-and-take' as needed to achieve the target stubble height of 15 cm within one day of occupation of a given paddock. The same animal group was used for the three paddocks within an experimental unit.

Grazing for all treatments initiated when one of the treatments reached 95% light interception (LI) [25] determined using the canopy analyzer LAI-2200 (LI-COR®, USA). The average light interception was 95, 96, and 90% for control, fertilized, and mixed treatments, respectively. Five growth cycles occurred in each rainy season with a rest period (RP) between them varying from 21 to 42 d (Table 1), due to seasonal and annual growth variation for time to reach 95% LI. A reserve palisadegrass pasture was used to keep the animals during the rest periods.

# Response variables

## Herbage and canopy characteristics

All samples were collected from the second paddock on the second grazing day (Fig 2). Before putting the grazing animals on paddocks, the canopy height was estimated using a sward stick at 16 random points. The average canopy height was calculated and two samples representing the average herbage mass were taken using 0.25-m$^2$ quadrats. The herbage biomass at pre-grazing was harvested at 2 cm ~~from the ground level~~. A subsample taken from the herbage biomass (fertilized and control treatment) was separated into leaf blade (leaf biomass), stem plus sheath (stem biomass), and senescent biomass (brown leaf and stem attached to the plant). When present, the inflorescence was included in the stem fraction. For the mixed treatment, the entire sample was hand separated to measure the proportion of legume and grass (botanical composition). For calculation of herbage biomass at pre-grazing [kg of dry mass (DM) ha$^{-1}$], all aboveground biomass (leaf + stem + senescent biomass) was considered; for the mixed treatment, total grass plus legume was considered. These fractions and the remaining non-separated herbage were oven-dried at 55°C for 72 h to calculate leaf:stem ratio (L:S). Herbage accumulation rate (kg DM ha$^{-1}$ d$^{-1}$) was estimated as the difference between the herbage biomass at pre-grazing of the current growth cycle and the herbage biomass at post-grazing of the previous growth cycle (leaf + stem + senescent biomass was considered in this calculation), divided by the number of rest days between growth cycles (Table 1).

## Nitrogen uptake, fixation, and allocation

At the beginning of each growth cycle, samples of total aboveground biomass of forage peanut and non-$N_2$-fixing reference plants (weeds) including billygoat-weed (*Ageratum*

*conyzoides* L.), beggar-ticks (*Bidens pilosa* L.), coat-buttons (*Tridax procumbens* L.), gomphrena-weed (*Gomphrena celosioides* Mart.), cockroach berry (*Solanum capsicoides* All.), and sanguinarea [*Alternanthera ficoidea* (L.) P. Beauv.] were collected from the same area. Different reference plants were collected due to the low number of weeds in the paddock. Samples were ground using a Wiley mill (Marconi, Ma680) to pass through a 1-mm stainless steel screen. After samples were roller milled for 16 h [26], total N concentration, abundance of $^{15}N$ (‰) were analyzed using a mass spectrometer (model Delta V Advantage, Thermo Scientific) coupled to an elemental analyzer (model Flash 2000, Thermo Scientific) in the "John Day Stable Isotope Laboratory" at Embrapa Agrobiologia. The total aboveground N of forage peanut ($TAGN_{FP}$, kg N $ha^{-1}$) was calculated multiplying the N concentration by the herbage accumulation of forage peanut. The percentage of N derived from the atmosphere (Ndfa) for forage peanut was calculated using the equation described by Shearer and Kohl [27]:

$$Ndfa\ (\%) = \left(\frac{\delta^{15}N_{\mathrm{refence}} - \delta^{15}N_{legume}}{\delta^{15}N_{\mathrm{refence}} - \mathrm{B}}\right) \times 100$$

where $\delta^{15}N_{reference}$ is the $\delta^{15}N$ value detected in a reference plant (non-N fixing) growing in the same soil at the same growth cycle in the palisadegrass-forage peanut paddock, $\delta^{15}N_{legume}$ is the $\delta^{15}N$ abundance of the forage peanut, and *B* is the $\delta^{15}N$ value of the N in the legume obtained from $N_2$ fixation. We used the *B* value of −1.35‰ reported by Unkovich [28] for *Arachis hypogea* L. in Brazil. The $\delta^{15}N$ from reference plants ranged from 4.4 to 5.9‰ ± 0.6‰, with values changing with sampling date. The total contribution of N from biological $N_2$ fixation (BNF) was calculated by multiplying $TAGN_{FP}$ by the Ndfa [28].

## Statistical analysis

Data were analyzed using the MIXED procedure of the SAS statistical package (SAS Inst. Inc., Cary, NC, USA). Herbage biomass at pre-grazing, herbage accumulation rate, and morphological components of palisadegrass were analyzed with N management, growth cycle and their interaction as fixed effects, whereas paddock within N management and year were used as random effects. Morphological components of forage peanut, $TAGN_{FP}$, Ndfa, and BNF were analyzed with growth cycle as fixed effect and paddock within year as random effect. The Satterthwaite approximation was used to determine the denominator degrees of freedom for the test of fixed effects. The LSMEANS were compared using the PDIFF procedure by Fisher's protected least significant difference (LSD). Differences were considered significant at $P \leq 0.1$. Pearson correlation were carried out to explored the relationship between growth cycle (GC) and rainfall using the CORR procedure of the SAS.

# Results

Nitrogen fertilizer increased the herbage biomass compared with unfertilized palisadegrass and palisadegrass-forage peanut mixture, which did not differ from each other ($P$ = 0.028; Figure 3A). There was an approached difference in N management × growth cycle on the herbage accumulation rate ($P$ = 0.106; Figure 3B). The urea fertilization increased the herbage accumulation rate in the GC4 compared to control and mixed treatment. Regarding the GC effect within N management, no difference was found in the herbage accumulation rate among GC for the mixed system. The greatest herbage accumulation rate occurred in the GC2 for the control treatment and the GC4 for the fertilized treatment. There was a positive

correlation between rainfall and herbage accumulation rate (r = 0.18, $P = 0.085$). However, the correlation was weak.

**Fig 3. Herbage biomass (A) and herbage accumulation rate (B) of palisadegrass under three nitrogen management strategies (NM) for five growth cycles (mean ± SE bars).** Control: palisadegrass without N fertilizer or legume; Fertilized: palisadegrass fertilized with 150 kg N $ha^{-1}$ $year^{-1}$; and Mixed: palisadegrass mixed with forage peanut and no N fertilizer. *Growth cycle 1 (GC1) was not evaluated, because ~~the stubble mass was not performed after the staging grazing cycle~~ in September. †Non-significant. Data are means across 2 years, and 4 replicates (n = 8). ~~Different lowercase letter is different~~ according to the PDIFF procedure by LSD test ($P \leq 0.1$).

The mixed treatment was separated into palisadegrass and forage peanut to compare the morphological components of palisadegrass among treatments (Table 2). Canopy height, leaf biomass, and stem biomass of palisadegrass ~~at the pre-grazing event~~ were affected by NM and GC, while the L:S ratio was only affected by GC ($P \leq 0.1$). Nitrogen fertilization increased canopy height, leaf biomass, and stem biomass of palisadegrass compared with the control and mixed treatments. Canopy height, stem biomass, and L:S ratio of palisadegrass was similar among GC, except in GC5, which had the lowest canopy height. In contrast, the lowest stem biomass, and the greatest L:S ratio occurred ~~in~~ GC1. The greatest leaf biomass of palisadegrass was observed in GC1, while the lowest ~~mean~~ occurred ~~in~~ GC5. There ~~was a~~ positive correlation between rainfall and canopy height (r = 0.23, $P = 0.013$), leaf biomass (r = 0.31, $P < 0.001$), and L:S ratio (r = 0.17, $P = 0.064$).

**Table 1. Morphological components of palisadegrass under three nitrogen management strategies, and forage peanut biomass and nitrogen fixation of forage peanut in the mixed system, for two rainy seasons conducted during five growth cycles.**

| | Nitrogen management strategies (NM) | | | SE | Growth cycle (GC) | | | | | SE | *P*-value | | |
|---|---|---|---|---|---|---|---|---|---|---|---|---|---|
| | Control | Fertilized | Mixed[a] | | 1 | 2 | 3 | 4 | 5 | | NM | GC | NM×GC |
| *Grass* | | | | | | | | | | | | | |
| CH | 26b | 38a | 27b | 3 | 33a | 31a | 32a | 31a | 25b | 2 | 0.023 | 0.002 | 0.228 |
| Leaf | 1700b | 2490a | 1310c | 130 | 2230a | 1670bc | 1910b | 1970ab | 1390c | 130 | <0.001 | <0.001 | 0.567 |
| Stem | 1330ab | 1970a | 1070b | 280 | 1050b | 1510a | 1450a | 1750a | 1520a | 210 | 0.083 | 0.032 | 0.669 |
| Senescent | 1360 | 1390 | 1220 | 140 | 1010c | 1360b | 1220bc | 1270bc | 1940a | 140 | 0.942 | <0.001 | <0.001 |
| L:S ratio | 1.7 | 1.5 | 1.8 | 0.2 | 2.3a | 1.5b | 1.6b | 1.5b | 1.6b | 0.2 | 0.679 | <0.001 | 0.672 |
| *Le[illegible]me* | | | | | | | | | | | | | |
| CH | - | - | 7.4 | 1.3 | 8.7a | 7.8ab | 8.3a | 8.0a | 4.2b | 1.3 | - | 0.104 | - |
| FP | - | - | 239 | 126 | 263a | 321a | 304a | 249a | 60b | 126 | - | 0.143 | - |
| L:S ratio | - | - | 1.1 | 0.2 | 1.4a | 1.2abc | 1.3ab | 0.9bc | 0.8c | 0.2 | - | 0.092 | - |
| $TAGN_{FP}$ | - | - | 3.1 | 2.5 | 6.8a | 1.9b | 4.1ab | 2.1b | 0.6b | 2.5 | - | 0.099 | - |
| Ndfa | - | - | 82 | 2 | 79c | 81bc | 82ab | 82b | 85a | 2 | - | 0.029 | - |
| BNF | - | - | 2.5 | 2.0 | 5.5a | 1.5ab | 3.2a | 1.7ab | 0.5b | 2.0 | - | 0.010 | - |

[a] Mixed treatment was split in palisadegrass and forage peanut data.

BN[illegible] biological $N_2$ fixation (kg N $ha^{-1}$ $cycle^{-1}$); CH, canopy height (cm); FP, forage peanut biomass (kg DM $ha^{-1}$); leaf biomass (kg DM $ha^{-1}$); L:S, leaf: stem ratio; Ndfa, nitrogen derived from the atmosphere by forage peanut (%); SE, standard error; stem biomass (kg DM $ha^{-1}$); senescent biomass (kg DM $ha^{-1}$); $TAGN_{FP}$, total aboveground N of forage peanut (kg N $ha^{-1}$ $cycle^{-1}$).

Senescent biomass was affected by NM × GC interaction ($P < 0.001$; Fig 4). The senescent biomass varied with N management strategies during the growth cycles. The N fertilization resulted in less senescent biomass in GC1 and GC2 compared with control and mixed treatment, while an increase was observed in GC5 (Fig 4A). Regarding the GC effect within N management, the lowest senescent biomass was observed in the GC1, GC3, and GC4 for the fertilized, control, and mixed treatment, respectively (Fig 4B). The greatest senescent biomass was observed in GC2 and GC4 for the control treatment and in GC5 for fertilized and mixed treatments. There was a negative correlation between rainfall and senescent biomass (-0.28, $P = 0.003$), i.e., a rainfall reduction increased the senescent biomass of the palisadegrass.

**Fig 4. Nitrogen management strategies (NM) × growth cycle (GC) interaction effect on senescent biomass of palisadegrass (mean ± SE bars).** Control: palisadegrass without N fertilizer or legume; Fertilized: palisadegrass fertilized with 150 kg N ha$^{-1}$ year$^{-1}$; and Mixed: palisadegrass mixed with forage peanut and no N fertilizer. $^{\dagger}$Non-significant. Data are means across 2 years, and 4 replicates (n = 8). Different lowercase letter within NM are different according to the PDIFF procedure by LSD test ($P \leq 0.1$).

Forage peanut was separated from the palisadegrass in the mixed treatment to evaluate their pattern across the growth cycles (Table 2). ~~There was an approached difference of forage peanut biomass across growth cycle ($P = 0.143$).~~ Forage peanut canopy height, L:S ratio, total aboveground N ~~of forage peanut~~ ($TAGN_{FP}$), and ~~biological $N_2$ fixation (BNF)~~ showed a reduction mainly ~~in the~~ GC5 ($P \leq 0.1$), while nitrogen derived from the atmosphere (Ndfa) increased in the same cycle. There was a positive correlation between rainfall and canopy height (r = 0.41, $P = 0.017$) and L:S ratio (r = 0.54, $P = 0.001$). The bo[illegible]cal composition of forage peanut and palisadegrass in the mixed treatment differed among GC ($P < 0.001$),

~~h~~owever, the proportions did not change, except in GC5, which had the lowest forage peanut proportion (2% of the botanical composition). The proportion of forage peanut ~~among~~ GC1 to GC4, ranged between 7 to 9% of the botanical composition.

# Discussion

The goal of our study was to test whether there was a meaningful and measurable contribution of ~~the~~ N from forage peanut even at minimal participation in the mixed system. Nitrogen is generally the most limiting nutrient for plant growth~~,~~ enhances tillering [29], leaf number and/or leaf elongation [30], and is a major component of the chlorophyll molecule [31]. Results of studies that accounted for the capacity of forage peanut to fix the nitrogen derived from atmospheric (Ndfa) were consistent with our findings. Assessments developed in the Southeastern United States [12, 32] and Northeast and Southeastern Brazil [14, 33] have shown that the %Ndfa for rhizome peanut and forage peanut can vary between 60 to 96% regardless of ~~being in~~ monocultures or mixtures. However, ~~the $N_2$ input~~ by the forage peanut in our study was insufficient to promote palisadegrass growth beyond what was found under unfertilized palisadegrass management ~~or close to synthetic N fertilized systems.~~

In the present study, the total amount of ~~$N_2$ fixation~~ was 15.5 kg N ha$^{-1}$ ~~for~~ five growth cycles. The weather and soil conditions are considered key drivers that regulate ~~the biological nitrogen fixation (~~BNF~~)~~, mainly by affecting ~~the~~ bacterial activiti~~es, which are responsible for fixing the $N_2$~~ [34]. However, no correlation between rainfall and BNF was found in the present study (r = 0.26, $P$ = 0.128). Another assumption of the~~se~~ relatively low BNF was due to the reduced participation of forage peanut in the botanical composition (< 10%) ~~in~~ the mixed system. ~~The l~~egume proportion between 20 - 45% in the herbage biomass was considered sufficient to supply N to the agroecosystem [19], fixing between 50 - 100 kg N ha$^{-1}$ yr$^{-1}$ [35].

On the other hand, the N supply ~~through the~~ synthetic fertilizer enhanced herbage biomass by 33 and 49% and leaf biomass by 46 and 90% of palisadegrass compared with unfertilized palisadegrass and palisadegrass-forage peanut mixture, respectively (Fig 3; Table 2). ~~The N fertilization increases the cells production, which increases~~ leaf elongation [36]~~ and regeneration of the tiller population, by increasing tiller appearance rate and tiller death; these~~ factors can vary with ~~season due to the weather conditions, such as~~ temperature and rainfall [37]. In addition, the N fertilized treatment always reached the 95% light interception threshold before the other treatments. Therefore, the low N treatments (grass or mixture) ~~did not grow as much over the same amount of time as the N fertilized grass, which was reflected in herbage biomass and related measurements.~~

~~[illegible]e extra N may have contributed to competition for light among tillers, and the plant elongates the stem under these conditions in search of the light for the younger leaves [30]. As part of a canopy sward, there is no benefit for a plant to create relatively more leaf biomass when it cannot intercept anymore light than it already has [38], which may explain the greatest stem proportion and canopy height in the N fertilized treatment without effect on the L:S ratio among N management strategies.~~ In the present study, the rainfall was positively correlated with canopy height (r = 0.23, $P = 0.013$), leaf biomass (r = 0.31, $P < 0.001$), and L:S ratio (r = 0.17, $P = 0.064$) of palisadegrass. Stem elongation is not desirable for grazing animal nutrition, ~~basically for three reasons~~. ~~First, increasing~~ ~~the stem~~ can reduce the leaf proportion [39]~~;~~ ~~second, the stem~~ elongation increases the canopy height, which will affect the ingestive animal behavior, such as reducing the number of tongue sweeps, bite volume, and bite weight [39, 40]; and third, the stem acts as a support structure ~~of leaves, which has~~ secondary cell ~~walls, such as lignin, which~~ ~~are undigestible~~ components [41, 42]. ~~These factors can limit the selection of leaf and, consequently, reduces~~ animal gain [43].

Nitrogen fertilization combined with favorable weather conditions promoted greater green biomass (leaf and stem) and reduced the senescent biomass, while the N fertilization and drought conditions of the transition period between rainy to dry season may have increased ~~the~~ senescent biomass. A negative correlation occurred between senescent biomass and rainfall (-0.28, *P* = 0.003). ~~The nitrogen fertilization associated with rainfall reduction may have affected maturity stage in the last GC and may have hastened flowering stem elongation and leaf senescent compared to other treatments. The N fertilization reduce the leaf lifespan, which increase the senescent biomass compared with unfertilized grass [44].~~

Under regular weather conditions (rainfall, temperature, and photoperiod), warm-season $C_4$ grasses are typically more competitive than legumes, shifting the balance in mixed swards towards the grass [14]. This competition can reduce ~~the persistence of~~ forage peanut in mixed systems. Grass establishment, growth, and time to recover after grazing tend to be faster ~~than for~~ legumes [10]. ~~Our study evidenced~~ a reduction in the presence of forage peanut due to drought conditions during the transition period ~~of the~~ rainy to dry season. During the dry season, ~~leaf loss typically occurs for~~ forage peanut [45]. In Northwest Brazil, [17] reported that ~~the~~ drought stress reduced ~~the~~ forage peanut biomass in ~~the~~ mixed system ~~during the dry season.~~ On the other hand, in Northeast Brazil, [46] did not find a negative impact of the dry season on ~~the~~ forage peanut growth. The authors suggested positive legume performance due to the favorable climatic weather in the study region, even during the dry season. Thus, differences among Brazilian regions, weather conditions, and latitude play an important role in the forage peanut growth, mainly ~~in~~ the dry season. With the onset of the rainy season, forage peanut initiates growth. ~~Remarkably,~~ the transition period between dry to rainy season ~~plays an important role in the~~ forage peanut recovery due to the ~~low grass canopy height and biomass~~ and bare ground ~~space for emission of stolons and~~ germination of soil seed bank after the first rains [17, 47]~~, however, this~~ recovery occurs slowly [48].

Forage peanut presents a slow establishment when mixed with aggressive-growing grasses such as palisadegrass, however, over time this legume was able to compete and enhance its contribution in mixed system [49]. Besides, *Arachis pintoi* has great tolerance to grazing, mainly because the growing points are protected from grazing animals, unlike most other tropical legume species. Thus, it is possible to maintain residual leaf area and initial growth even when the plant is subjected to continuous stocking and intense defoliation [50]. Pereira [46] reported that 20.5 to 23.8% of forage peanut mixed with palisadegrass resulted in a liveweight gain per ha 29% greater than in N fertilized system (dose of 120 kg N $ha^{-1}$). Therefore, if our study had addressed nutritive value and animal performance, perhaps we would have found the mixed system benefits even at minimal legume participation.

Keeping the light interception between 90 and 95% with canopy height ranging from 26 to 32 cm for palisadegrass-forage peanut mixtures was considered the best grazing management to provide greater herbage accumulation rate, especially leaves [25]. However, in our study with a minimal proportion of forage peanut, it is necessary to keep the LI less than 90%. Canopy height control in mixed systems is important because two different plant species compete for light, water, and nutrient in the same area [51]. Studies have been shown that a canopy height of 20 cm as the optimum to guarantee the best forage peanut proportion, mainly in the first years after seeding without compromising the grass growth [15, 52]. The increase in grass canopy height can stimulate vertical growth of forage peanut, reducing the number of stolons and decreasing legume proportion in the mixed system. These changes in growth habit of forage peanut may limit its ability to compete with palisadegrass in mixed stands. In the current study, high canopy height might have delayed the full establishment of forage peanut in the mixed system, with their growth being limited by shading from palisadegrass [15].

Nutrient competition between grass and legume, the lack of N fertilization in the first years after seeding of the mixed system, and the slow establishment of forage peanut may have

resulted in low herbage biomass of both legume and grass in the mixed system. During the first years after seeding of grass-legume mixed pastures, N fertilization could be used, considering that legumes have limited capability to fix atmospheric $N_2$ during initial developmental stages [35]. Further studies are required to evaluate whether the N fertilization could hasten the legume proportion and provide nutrients to sustain grass growth, at least in the mixed system initial years. The ultimate goal is to develop a sustainable mixed system that provides quality feed to the grazing animal, reflecting on the cost reduction to the farmer and the environmental impacts of synthetic N fertilizers. Therefore, further studies are required to test whether there is a meaningful and measurable contribution of the forage peanut on nutritional value and animal performance, even at minimal legume participation.

# Conclusions

The small amount of forage peanut in the mixed system presented similar results to the unfertilized palisadegrass in most response variables. Botanical composition fluctuated according to the growth cycle, with forage peanut disappearance at the beginning of the transition from rainy to dry season. Biological $N_2$ fixation and $TAGN_{FP}$ decreased with the reduction of forage peanut biomass, which were affected by periodic dry weather conditions during the season. In this study, the forage peanut inclusion was not enough to guarantee better results of the mixed system when compared to the N fertilized palisadegrass. The N fertilization enhanced the herbage biomass, especially the proportion of leaves.

The evaluation during the growth cycles demonstrated the growth pattern of the grass in a tropical environment as well as forage peanut. However, the legume was less tolerant to lack of water with the beginning of the transition to the dry season. Different management strategies such as the beginning of the grazing less than 90% of light interception or lower

stubble heights should be employed, considering that legumes ($C_3$) and grasses ($C_4$) require different management.

# Acknowledgements

The authors thank the members of UnespFOR (Brazilian forage Team) for the contributions during the field trial setup.

# References

**1.** Silva VJ, Pedreira CGS, Sollenberger LE, Silva LS, Yasuoka JI, Almeida ICL. Canopy height and nitrogen affect herbage accumulation, nutritive value, and grazing efficiency of 'Mulato II' Brachiariagrass. Crop Sci. 2016; 56:2054–2061. doi: 10.2135/cropsci2015.12.0764

**2.** Boddey RM, Macedo R, Tarré RM, Ferreira E, Oliveira OC, Rezende CP, et al. Nitrogen cycling in *Brachiaria* pastures: The key to understanding the process of pasture decline. Agric. Ecosyst. Environ. 2004; 103:389–403. doi: 10.1016/j.agee.2003.12.010

**3.** Sollenberger LE, Newman YC, Vendramini JMB. General guidelines for managing pastures for dairy cows. Coop. Ext. Serv. Publ. AG162. Inst. Food Agric. Sci., Univ. Florida, Gainesville. 2009.

**4.** Delevatti LM, Cardoso AS, Barbero RP, Leite RG, Romanzini EP, Ruggieri AC, et al. Effect of nitrogen application rate on yield, forage quality, and animal performance in a tropical pasture. Sci. rep. 2019; 9.7596. doi: 10.1038/s41598-019-44138-x

**5.** Gerber PJ, Steinfeld H, Henderson B, Mottet A, Opio C, Dijkman J, Falcucci A, Tempio G. Tackling climate change through livestock – A global assessment of emissions and

mitigation opportunities. Food and Agriculture Organization of the United Nations (FAO), Rome. 2013.

**6.** Zaman M, Saggar S, Blennerhassett JD, Singh J. Effect of urease and nitrification inhibitors on N transformation, gaseous emissions of ammonia and nitrous oxide, pasture yield and N uptake in grazed pasture system. Soil Biol. Biochem. 2009; 41:1270–1280. doi: 10.1016/j.soilbio.2009.03.011

**7.** Zaman M, Nguyen ML, Šimek M, Nawaz S, Khan MJ, Babar MN, et al. Emissions of nitrous oxide ($N_2O$) and di-nitrogen ($N_2$) from the agricultural landscapes, sources, sinks, and factors affecting $N_2O$ and $N_2$ ratios. Greenhouse gases-emission, measurement and management (Ed. Guoxiang Liu), 1-32. 2012.

**8.** Muir JP, Pitman WD, Dubeux JC, Foster JL. The future of warm-season, tropical and subtropical forage legumes in sustainable pastures and rangelands. Afr. J. Range For. Sci. 2014; 31:187–198. doi: 10.2989/10220119.2014.884165

**9.** Sprent JI. Evolving ideas of legume evolution and diversity: a taxonomic perspective on the occurrence of nodulation. New Phytol. 2007; 174:11–25. doi: 10.1111/j.1469-8137.2007.02015.x

**10.** Muir JP, Pitman WD, Foster JL. Sustainable, low-input, warm-season, grass-legume grassland mixtures: Mission (nearly) impossible? Grass Forage Sci. 2011; 66:301–315. doi: 10.1111/j.1365-2494.2011.00806.x

**11.** Williams MJ, Hammond AC, Kunkle WE, Spreen TH. Stocker Performance on Continuously Grazed Mixed Grass-Rhizoma Peanut and Bahiagrass Pastures. J. Prod. Agric. 1991; 4:19–24. doi: 10.2134/jpa1991.0019

**12.** Santos ER, Dubeux JCB, Sollenberger LE, Blount ARS, Mackowiak C, DiLorenzo N, et al. Herbage responses and biological $N_2$ fixation of bahiagrass and

rhizoma peanut monocultures compared with their binary mixtures. Crop Sci. 2018; 58:1–15. doi: 10.2135/cropsci2018.02.0128

**13.** Jaramillo DM, Dubeux JCB, Mackowiak C, Sollenberger LE, DiLorenzo N, Rowland DL, et al. Annual and perennial peanut mixed with 'Pensacola' bahiagrass in North Florida." Crop Sci. 2018; 58:982–992. doi: 10.2135/cropsci2017.09.0542

**14.** Alencar NM, Vendramini JMB, Santos AC, Silveira ML, Dubeux JCB, Sousa LF, et al. Herbage characteristics of pintoi peanut and palisadegrass established as monoculture or mixed swards. Crop Sci. 2018; 58:1–7. doi: 10.2135/cropsci2017.09.0538

**15.** Tamele OH, Sá OAAL, Bernardes TF, Lara MAS, Casagrande DR. Optimal defoliation management of brachiaria grass–forage peanut for balanced pasture establishment. Grass Forage Sci. 2018; 73:522–531. doi: 10.1111/gfs.12332

**16.** Hernandez M, Argel PJ, Ibrahim MA, Mannetje LT. Pasture production, diet selection and liveweight gains of cattle grazing *Brachiaria brizantha* with or without *Arachis pintoi* at two stocking rates in the Atlantic zone of Costa Rica. Trop. Grassl. 1995; 29:134–141.

**17.** Andrade CMS, Garcia R, Valentim JF, Pereira OG. Productivity, utilization efficiency and sward targets for mixed pastures of marandugrass, forage peanut and tropical kudzu. R. Bras. Zootec. 2012; 41:512–520. doi: 10.1590/S1516-35982012000300006

**18.** Andrade CMS, Garcia R, Valentim JF, Pereira OG. Response of *Arachis pintoi* to grazing intensity when associated with different grasses. In: International Grassland Congress, Dublin. Proceedings. Wageningen: Wageningen Academic Publishers, 2005. p. 347.

**19.** Thomas RJ. The role of the legume in the nitrogen cycle of productive and sustainable pastures. Grass Forage Sci. 1992; 47:133–142. doi: 10.1111/j.1365-2494.1992.tb02256.x

**20.** USDA. Soil Survey Staff. 2014. Keys to Soil Taxonomy, 12th ed. USDA-Natural Resources Conservation Service, Washington, DC. 2014.

**21.** van Raij B, Andrade JC, Cantarella H, Quaggio JA. Análise química para avaliação da fertilidade de solos tropicais. IAC, Campinas; 2001. (In Portuguese).

**22.** Claessen MEC. Manual de métodos de análise de solo/Centro Nacional de Pesquisa de Solos. 2 ed. rev. Atual. Rio de Janeiro, EMBRAPA-CNPS. Documentos; 1997. (In Portuguese).

**23.** Werner JC, Paulino VT, Cantarella H, Andrade NO, Quaggio JA. Forrageiras. In: van Raij B, Cantarella H, Quaggio JA, Furlani AMC. Recomendações de adubação e calagem para o Estado de São Paulo. Campinas: Instituto Agronômico, 1996p. 263-273. (In Portuguese).

**24.** Allen VG, Batello C, Berretta EJ, Hodgson J, Kothmann M, Li X, et al. An international terminology for grazing lands and grazing animals. Grass Forage Sci. 2011; 66:2–28. doi: 10.1111/j.1365-2494.2010.00780.x

**25.** Pereira JC, Gomes FK, Oliveira MD, Lara MA, Bernardes TF, Casagrande DR. Defoliation management affects morphogenetic and structural characteristics of mixed pastures of brachiaria grass and forage peanut. Afr. J. Range For. Sci. 2017; 34:13–19. doi: 10.2989/10220119.2017.1315960

**26.** Arnold SL, Schepers JS. A simple roller-mill grinding procedure for plant and soil samples. Commun. Soil Sci. Plan. 2004; 35:537–545. doi: 10.1081/CSS-120029730

**27.** Shearer G, Kohl D. $N_2$-fixation in field settings: Estimations based on natural $^{15}N$ abundance. Aust. J. Plant Physiol. 1986; 13:699–756. doi: 10.1071/PP9860699

**28.** Unkovich M, Herridge D, Peoples M, Cadisch G, Boddey R, Giller K, et al. Measuring plant-associated nitrogen fixation in agricultural systems. In: Unkovich M, Herridge D, Peoples M, Cadisch G, Boddey R, Giller K, et al. Australian Centre for International Agricultural Research (ACIAR), 2008.

**29.** Silva DRG, Costa KAP, Faquin V, Oliveira IP, Bernardes TF. Rates and sources of nitrogen in the recovery of the structural and productive characteristics of marandu grass. Rev. Cienc. Agron. 2013; 44, 184–191. doi: 10.1590/S1806-66902013000100023 (Abstract in English)

**30.** Santana SS, Brito LF, Azenha MV, Oliveira AA, Malheiros EB, Ruggieri AC, et al. Canopy characteristics and tillering dynamics of Marandu palisade grass pastures in the rainy–dry transition season. Grass Forage Sci. 2016; 72:261–270. doi: 10.1111/gfs.12234

**31.** Chapman SC, Barreto HJ. Using a chlorophyll meter to estimate specific leaf nitrogen of tropical maize during vegetative growth. Agron. J. 1997; 89:557–562. doi: 10.2134/agronj1997.00021962008900040004x

**32.** Dubeux JCB, Blount ARS, Mackowiak C, Santos ERS, Pereira Neto JD, Riveros U, et al. Biological $N_2$ fixation, belowground responses, and forage potential of rhizoma peanut cultivars. Crop Sci. 2017; 57:1027–1038. doi: 10.2135/cropsci2016.09.0810

**33.** Carvalho LR, Pereira LET, Hungria M, Camargo PBD, Silva SC. Nodulation and biological nitrogen fixation (BNF) in forage peanut (*Arachis pintoi*) cv. Belmonte subjected to grazing regimes. Agric. Ecosyst. Envir. 2019; 278:96–106. doi: 10.1016/j.agee.2019.02.016

**34.** Phelan P, Moloney AP, McGeough EJ, Humphreys J, Bertilsson J, O'Riordan EG, et al. Forage legumes for grazing and conserving in ruminant production systems. Crit. Rev. Plant Sci. 2015; 34:281–326. doi: 10.1080/07352689.2014.898455

**35.** Andrade CMS, Wadt PGS, Zaninetti RA, Valentim JF. Recomendação de calagem e adubação para pastagens no Acre. Circ. Tec. 46. Embrapa. 2014. Available from: https://ainfo.cnptia.embrapa.br/digital/bitstream/item/115015/1/25378.pdf. (In Portuguese)

**36.** Volenec JJ, Nelson CJ. Responses of Tall Fescue Leaf Meristems to N Fertilization and Harvest Frequency 1. Crop Sci. 1983; 23:720–724. doi: 10.2135/cropsci1983.0011183X002300040028x

**37.** Caminha FO, Silva SC, Paiva AJ, Pereira LET, Mesquita P, Guarda VDA. Stability of tiller population of continuously stocked marandu palisade grass fertilized with nitrogen. Pesq. Agropec. Bras. 2010; 45:213–220. doi: 10.1590/S0100-204X2010000200013 (Abstract in English)

**38.** Calvière I, Duru M. The effect of N and P fertilizer application and botanical composition on the leaf/stem ratio patterns in spring in Pyrenean meadows. Grass Forage Sci. 1999; 54:255–266. doi: 10.1046/j.1365-2494.1999.00178.x

**39.** Fonseca L, Mezzalira JC, Bremm C, Gonda HL, Carvalho PDF. Management targets for maximising the short-term herbage intake rate of cattle grazing in Sorghum bicolor. Livest. Sci. 2012; 145:205–211. doi: 10.1016/j.livsci.2012.02.003

**40.** Burns JC, Sollenberger LE. Grazing behavior of ruminants and daily performance from warm-season grasses. Crop Sci. 2002; 42:873–881. doi: 10.2135/cropsci2002.8730

**41.** Euclides VP, Montagner DB, Macedo MCM, Araújo AR, Difante GS, Barbosa RA. Grazing intensity affects forage accumulation and persistence of Marandu palisadegrass in the Brazilian savannah. Grass Forage Sci. 2019; 74:450–462. doi: 10.1111/gfs.12422

**42.** Chen L, Auh C, Chen F, Cheng X, Aljoe H, Dixon RA, et al. Lignin deposition and associated changes in anatomy, enzyme activity, gene expression, and ruminal

degradability in stems of tall fescue at different developmental stages. J. Agric. Food Chem. 2002; 50:5558–5565. doi: 10.1021/jf020516x

**43.** Newman YC, Sollenberger LE, Kunkle WE, Chambliss CG. Canopy height and nitrogen supplementation effects on performance of heifers grazing limpograss. Agron J. 2002; 94:1375–1380. doi: 10.2134/agronj2002.1375

**44.** Duru M, Ducrocq H. Growth and senescence of the successive leaves on a Cocksfoot tiller. Effect of nitrogen and cutting regime. Ann. Bot. 2000; 85:645-653. doi: 10.1006/anbo.1999.1117

**45.** Gomes FK, Oliveira MDBL, Homem BGC, Boddey RM, Bernardes TF, Gionbelli MP, et al. Effects of grazing management in brachiaria grass-forage peanut pastures on canopy structure and forage intake. J. Anim. Sci. 2018; 96:3837–3849. doi: 10.1093/jas/sky236

**46.** Pereira JM, Rezende CDP, Borges AMF, Homem BGC, Casagrande DR, Macedo TM, et al. Production of beef cattle grazing on *Brachiaria brizantha* (Marandu grass)-*Arachis pintoi* (forage peanut cv. Belomonte) mixtures exceeded that on grass monocultures fertilized with 120 kg N/ha. Grass Forage Sci. 2020; 75:28–36. doi: 10.1111/gfs.12463

**47.** Andrade CMSD, Garcia R, Valentim JF, Pereira OG. Grazing management strategies for massaigrass-forage peanut pastures: 1. Dynamics of sward condition and botanical composition. R. Bras. Zootec. 2006; 35:334–342. doi: 10.1590/S1516-35982006000200002

**48.** Ribeiro OL, Cecato U, Roma CFC, Faveri JC, Gomes JAN, Barbero ML. Forage production and performance animal in coastcross intercropping or not with *Arachis pintoi*, with or without nitrogen. Acta Sci. Anim. Sci. 2008; 30:371–378. doi: 10.4025/actascianimsci.v30i4.6466 (Abstract in English)

**49.** Argel PJ, Pizarro EA. Germplasm case study: *Arachis pintoi*. In.: Pastures for the Tropical Lowlands: CIAT's (Centro International de Agricultura Tropical) Contribution. Cali, Colombia; 1992.

**50.** Lima JA, Pinto JC, Evangelista AR, Santana RAV. Amendoim Forrageiro (*Arachis pintoi* Krapov. & Gregory); 2003. Available from: http://livraria.editora.ufla.br/upload/boletim/extensao-tmp/boletim-extensao-001.pdf. (In Portuguese).

**51.** Ruggieri AC, Galzerano L, Silva WL. Interação entre plantas em ambientes de pastagens. In.: Reis RA, Bernardes TF, Siqueira GR (Eds). Forragicultura: Ciência, Tecnologia e Gestão dos Recursos Forrageiro. Jaboticabal: Gráfica e editora multipress; 2013. (In portuguese)

**52.** Homem BG, Rosa AD, Ferreira IM, Cruvinel IA, Lara MA, Bernardes TF, et al. Increasing the population of forage peanut in a mixed pasture by controlling the canopy height. Grass Forage Sci. 2019; 74:571–575. doi: 10.1111/gfs.12436

Fig 1. Cumulative rainfall and mean air temperatures (maximum, mean, and minimum) for five growth cycles (GC) in Jaboticabal, Brazil.

Click here to access/download;Figure;Fig1.tif.tif

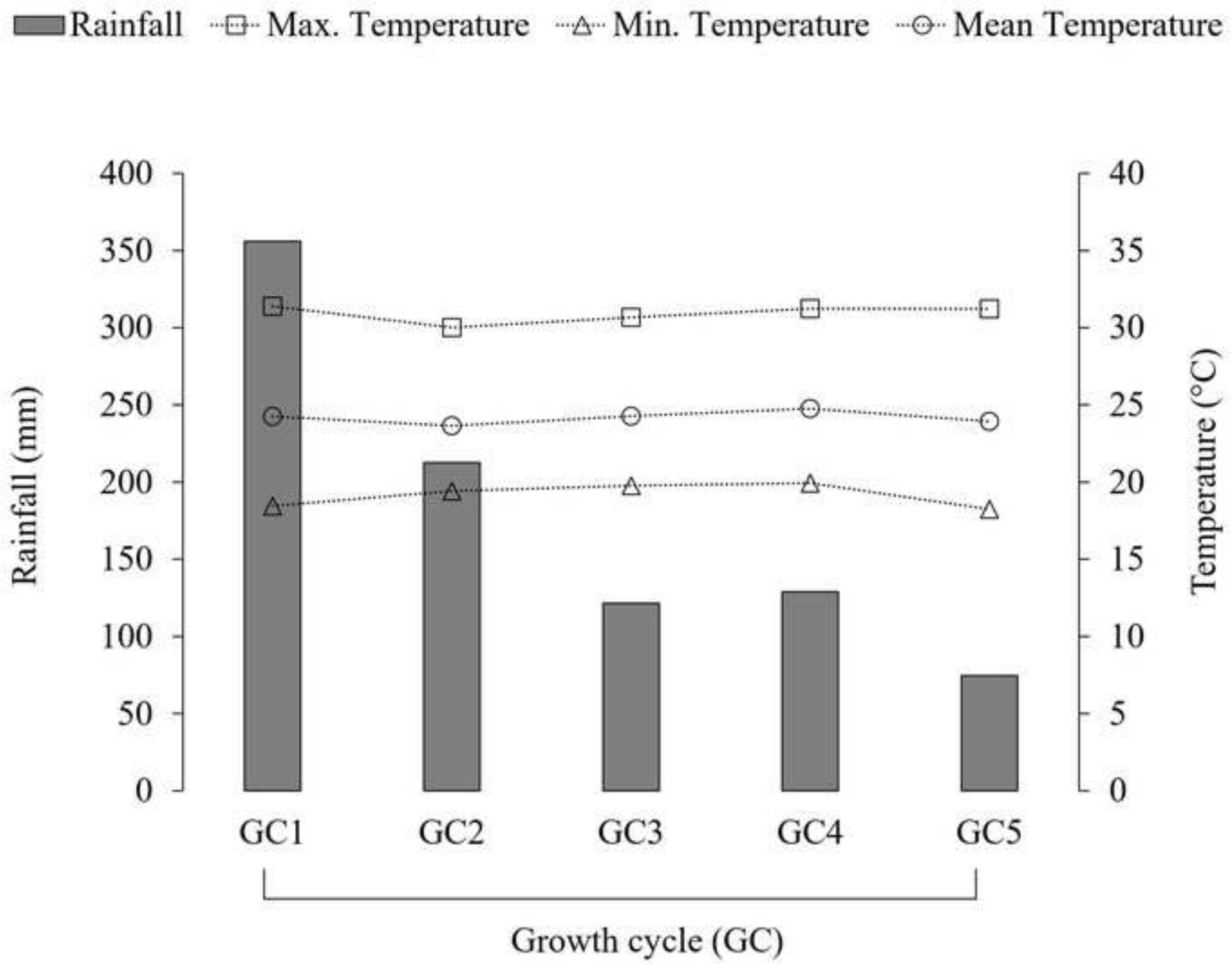

Fig 2. ~~Fig 2.~~ Schematic representation of the experimental design. Three treatments with four replications, arranged in a completely randomized design. Experimental unit

Click here to access/download;Figure;Fig2.tif.tif

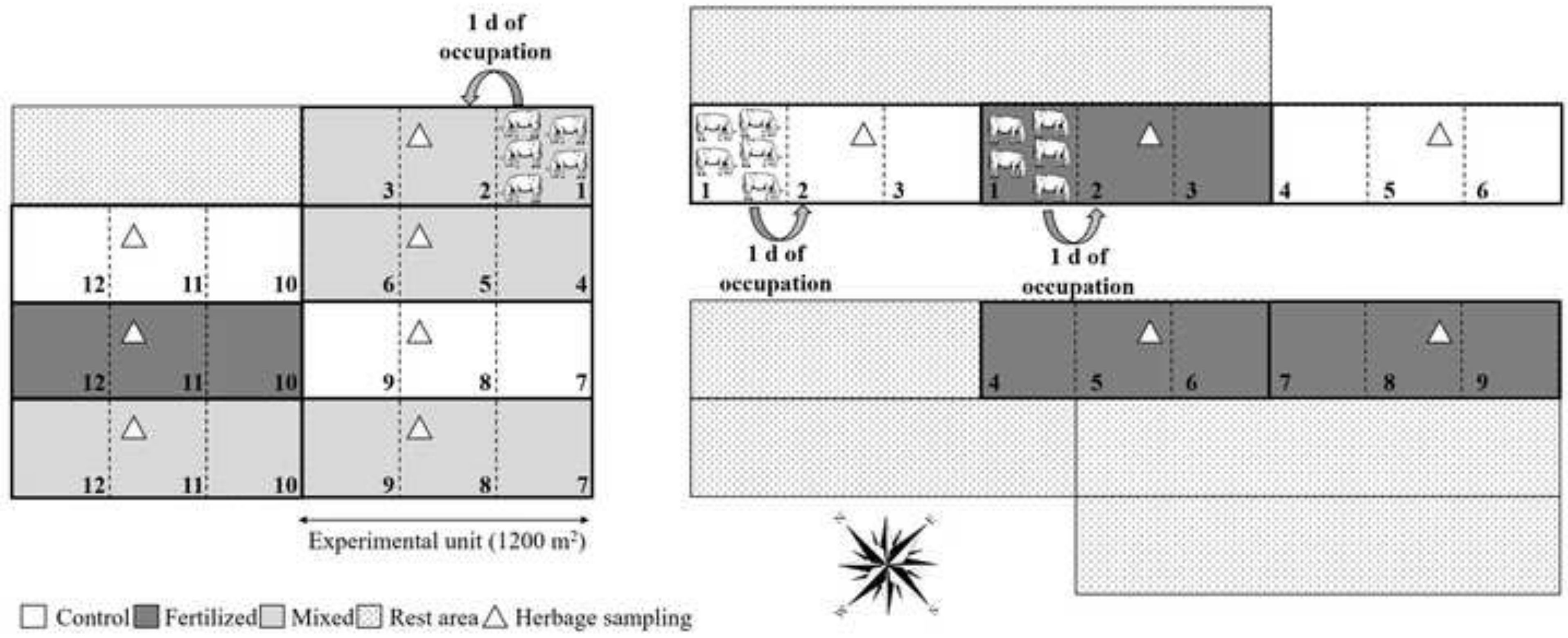

Fig 3 Herbage biomass (A) and herbage accumulation rate (B) of palisadegrass under three nitrogen management strategies (NM)

Click here to access/download;Figure;Fig3.tif.tif

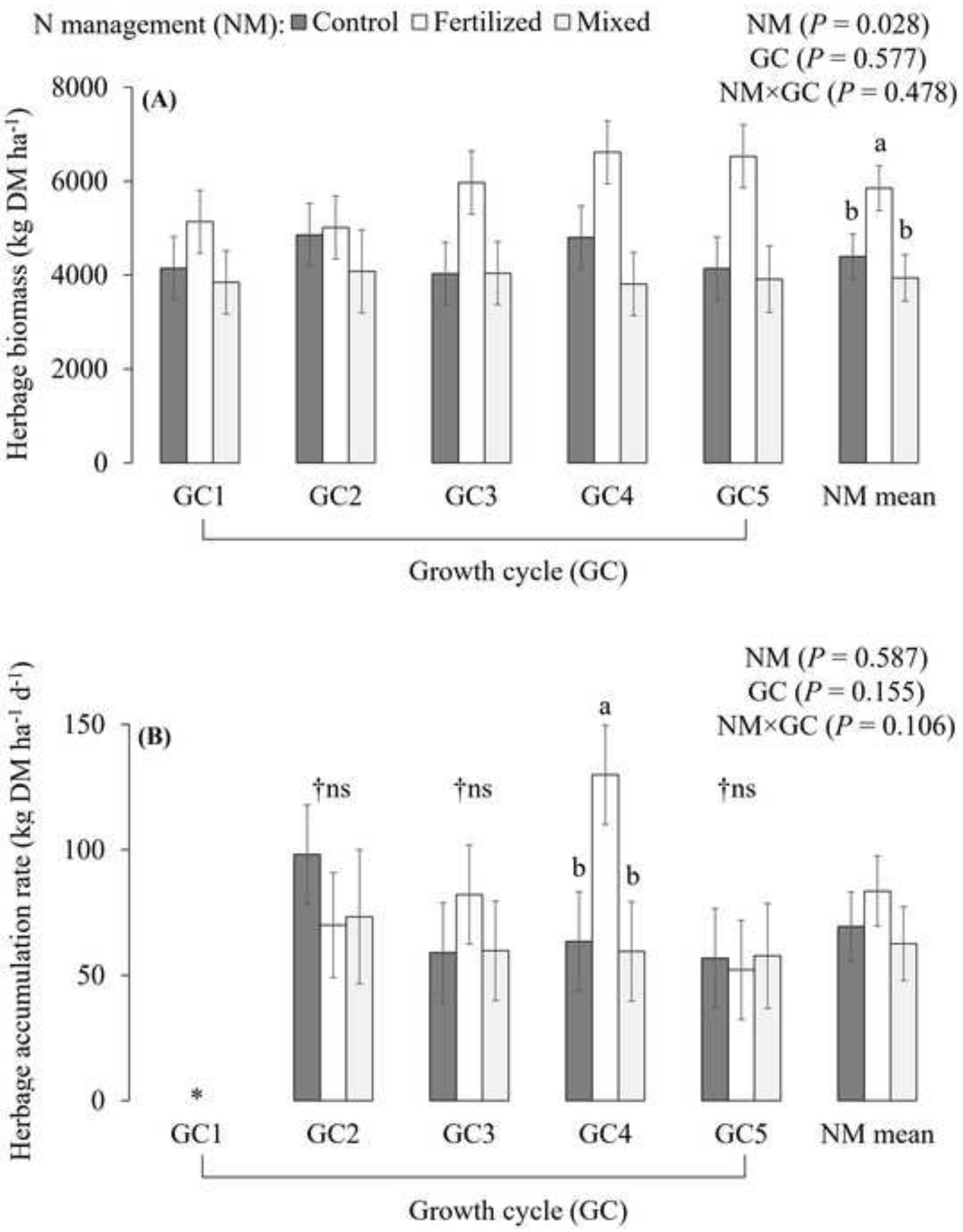

Fig. 4. Nitrogen management strategies (NM) × growth cycle (GC) interaction effect on senescent biomass of palisadegrass (mean ± SE bars).

Click here to access/download;Figure;Fig4.tif.tif

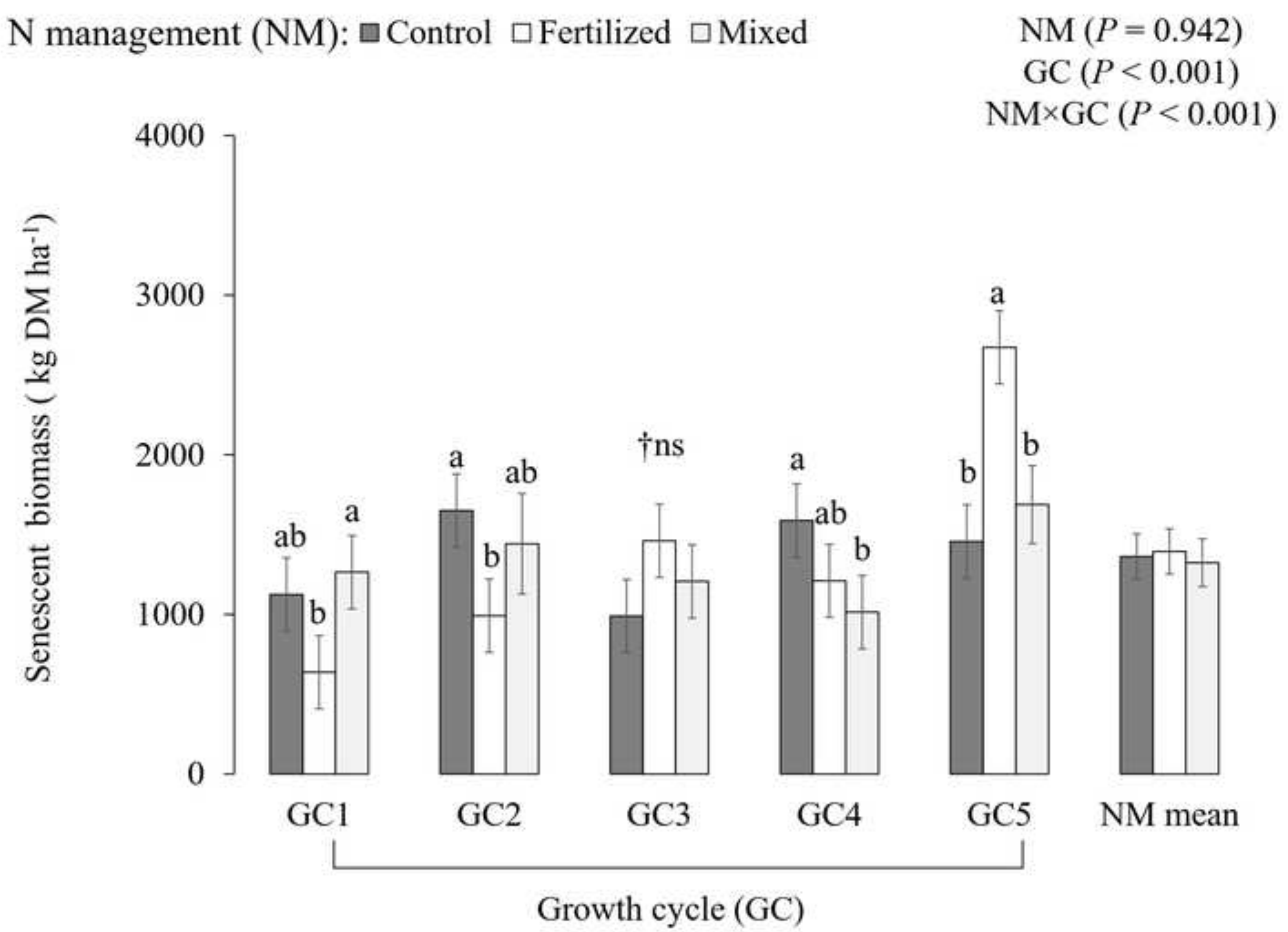

# Could forage peanut in low proportion replace N fertilizer in livestock systems?

Impact of N fertilization or the presence of forage peanut on the herbage structure of palisadegrass under grazing

Vanessa Z. Longhini[1*], Abmael S. Cardoso[1], Andressa S. Berça[1], Robert M. Boddey[2], Ricardo A. Reis[1], José C.B. Dubeux[3], Jr., Ana C. Ruggieri[1]

[1] Department of Animal Science, São Paulo State University, via de acesso Professor Paulo Donato Castellane, Jaboticabal, SP, 14884-900, Brazil

[2] Embrapa Agrobiologia, Antiga Rodovia Rio-São Paulo, km 47. Seropédica, RJ, 23891-000, Brazil

[3] University of Florida, North Florida Research and Education Center, Marianna, FL, 32446, United States

* Corresponding author:

E-mail: vanessa.longhini@unesp.br (VZL)

# Abstract

Palisadegrass [*Urochloa brizantha* (Hochst. ex A. Rich.) R. D. Webster cv. ‘Marandu’] is widely used in Brazil and typically managed with little or no N fertilizer, leading to pasture decline. The current relationship between beef price and fertilizer cost in Brazil does not favor fertilizer use in pastures. Legume inclusion is an alternative to add N, but oftentimes legumes do not reach significant proportion (> 30%) in pasture botanical composition. This study evaluated herbage responses to N inputs and vegetation typespasture species composition

during the establishment, under rotational intermittent stocking. Treatments included palisadegrass-forage peanut (*Arachis pintoi* Krapov. & W.C. Greg. cv Amarillo) mixture (mixed), unfertilized palisadegrass (control), and palisadegrass fertilized with 150 kg N ha$^{-1}$ yr$^{-1}$ (fertilized). Treatments were applied for two grazing rainy trial seasons with five grazing growth cycles (GC) evaluations. Response variables included herbage biomass, herbage accumulation, morphological components, total aboveground N of forage peanut (TAGN$_{FP}$), and contribution of biological $N_2$ fixation (BNF). Pre-grazingH herbage biomass at pre-grazing was greater for fertilized palisadegrass [58530 kg dry matter (DM) ha$^{-1}$] than for the palisadegrass-forage peanut mixture (3940880 kg DM ha$^{-1}$), while the unfertilized palisadegrass (4400 kg DM ha$^{-1}$) did not differ from the mixed pasture. Nitrogen fertilizer increased leaf mass of palisadegrass (24970 kg DM ha$^{-1}$) compared with the control and mixed treatments (1700 and 1310 kg DM ha$^{-1}$, respectively). The percentage of N derived from the atmosphere (%Ndfa) for forage peanut ranged from 79 to 85% and BNF ranged from 0.75 to 5.5 kg N ha$^{-1}$ cycle$^{-1}$. Overall, benefits from forage peanut were minimal because legume percentage was less than< 10%. Beginning a grazing cycle before 950% canopy light interception or imposing a shorter post-grazing stubble height may aid in reducing shading to the decumbent-growing forage peanut when planted with palisadegrass.

# Introduction

In Brazil, grasses of the genus *Urochloa* cover an estimated 80 million ha. ‘Marandu’ palisadegrass [*Urochloa brizantha* (Hochst. ex A. Rich.) R. D. Webster cv. syn. *Brachiaria brizantha* (Hochst. ex A. Rich.) Stapf.], the most used palisadegrass cultivar, is estimated to cover 56% of these this area [1]. Low-input systems prevail in Brazilian livestock operations with minimal applications of so limestone, gypsum, and chemical N fertilizersapplications are

rare. The combination of low soil fertility, lack of chemical fertilizationN fertilizer application, and overgrazing results in pasture degradation or pasture decline [2].

Nitrogen is a key element to improve pasture productivity [3] and many warm-season $C_4$ grasses respond positively to N fertilization by increasing increased herbage accumulation and improving improved crude proteinnutritive value [4]. However, this response can variesvary with the environment, species, and management practices. Herbage accumulation of palisadegrass increased with increasing N fertilizer applications from 90 to 270 kg $ha^{-1}$, but there was a diminished increase in herbage accumulation per unit of N applied, as the amount of N fertilizer increased [4]. In this case, tripling the amount of N applied increased herbage accumulation by only 18%1.18 times and increased potential for negative environmental impacts. Nitrogen fertilizer is manufactured using fossil fuel, which gives itresults in a significant carbon footprint [5]. Furthermore, N fertilizer may be lost as nitric oxide (NO also called nitrogen oxide), nitrogen dioxide ($NO_2$), nitrous oxide ($N_2O$), di-nitrogen ($N_2$), ammonia ($NH_3$), or via nitrate ($NO_3^-$) leaching [6, 7]. These processes maySeveral of these processes contribute to climate change (e.g., $N_2O$ emission) or increase $NO_3^-$ nitrate concentrations in surface water bodies and groundwater.

Integrating legumes into grazing systems is an alternative to mineral N fertilizer applications that may reduce the negative environmental impacts caused by N fertilization [8]. Legumes add N to the system via symbiotic biological dinitrogen $N_2$ fixation (BNF) between legume and rhizobium bacteria in root nodules [9]. In addition to reduced negative environmental impacts, l Legumes can also reduce production costs compared with N fertilizer [8, 10].

The use of grass-legume mixed pastures in tropical regions is less commonplace than in temperate regions. In North America, mixing grasses and legumes is a more common

practice [11], ~~but it is still currently being investigated~~that requires further study [12, 13]. In Brazil, few studies have evaluated grass-legume mixtures under grazing. Challenges to legume use have been attributed to poor establishment and persistence compared with grasses [10, 14, 15]. Forage peanut (*Arachis pintoi* Krapov. & W.C. Greg. cv. Amarillo) is a legume that has ~~a~~ good potential ~~to grow in~~for mixtures in Brazil because it persists well when mixed with fast-growing grasses such as *Urochloa* [16, 17]. A stoloniferous and prostrate growth habit makes the forage peanut ideal for grazing because its meristems are protected from grazing animals [18]. ~~In~~ Under tropical conditions, Tamele [15] reported that palisadegrass managed at a 20-cm canopy height improved forage peanut establishment in mixed systems. However, further research is necessary to determine if N from forage peanut ~~growing~~ grown in mixture with a grass is able to ~~replace~~ substitute for synthetic N fertilizer~~, especially during the establishment period~~. The proportion of the legume in the mixture is important to determine the benefits of the association. According to Thomas [19], proportions between 20 - 45% in the herbage biomass was considered sufficient to supply N to the agroecosystem. However, not much has been done in terms of determining at which level the legume becomes important, or even if, at low participation, the legume is still able to benefit the overall system.

We hypothesized that ~~during the establishment of~~the forage peanut mixed with non-N fertilized grass ~~the herbage structures such as herbage accumulation, green herbage mass, leaf mass, and leaf:stem ratio~~ would ~~be improved through~~benefit the herbage responses through BNF, compared with unfertilized grass in a monoculture. ~~-~~However, the proportion of forage peanut in the sward in the present study was low. Thus, we decided to test whether there is a meaningful and measurable contribution of the N from forage peanut even at minimal participation and whether this contribution could be close to those systems using synthetic N fertilizer~~We also hypothesized that mixed swards would have similar herbage accumulation~~

~~compared to N fertilized swards~~. Therefore, this study evaluated the performance of palisadegrass under three ~~sward~~ nitrogen management strategies (unfertilized, N fertilized ~~with 150 kg N ha$^{-1}$ yr$^{-1}$~~, or mixed with the legume forage peanut), aiming to identify how different N inputs may affect ~~pasture~~ grass responses ~~during the establishment period~~. ~~A low presence of forage peanut in the present study occurred during the establishment period. However, we decided to continue the experiment to test if a low amount of forage peanut mixture with palisadegrass would result in any contribution to the mixed system.~~

# Materials and methods

## Ethical approval

The procedures involving animals were approved by the Institutional Animal Care and Use Committee at São Paulo State University, SP, Brazil (No. 10356/14-CEUA).

## Experimental site

This study was conducted at Sao Paulo State University, Jaboticabal, Sao Paulo, Brazil (21°14’~~05”~~ S and~~,~~ 48°17’~~09”~~ W; ~~61~~586 m a~~bove sea~~ l~~evel~~) during two ~~grazing~~ rainy ~~trials~~ seasons (November 2016 to March 2017 and December 2017 to April 2018). The soil at the experimental site is classified as an Oxisol [~~19~~20]. Soil samples (0- to 20-cm depth) collected in 2014, 2017, and 2018 indicated the following means: pH ($CaCl_2$) 5.3; organic matter 32.4 g kg$^{-1}$; cation exchange capacity 74.8 mmolc dm$^{-3}$; P (ion-exchange resin extraction method) 10.9 mg dm$^{-3}$; Mehlich$^{-1}$ extractable Ca 28.3 mmolc dm$^{-3}$; Mehlich$^{-1}$ extractable Mg 9.7 mmolc dm$^{-3}$; Mehlich$^{-1}$ extractable K 4.2 mmolc dm$^{-3}$; base saturation 561 g kg$^{-1}$, respectively [2~~0~~1]. Soil texture was 340 g kg$^{-1}$ sand, 140 g kg$^{-1}$ silt and 520 g kg$^{-1}$ clay [22~~2~~]. The annual rainfall

**Formatted:** Font: 16 pt

**Formatted:** Indent: First line: 0.5"

in this area is 1314 mm and mean annual air temperature is 22.7 °C (2010-2018). Rainfall and mean temperature data from the experimental site were extracted from a dataset belonging to the Agrometeorology section of the Department of Exact Sciences (Fig 1).

**Fig 1. ~~Monthly~~ Cumulative rainfall and ~~monthly~~ mean air temperatures (maximum, mean, and minimum) for five growth cycles (GC) ~~from November 2016 to May 2018~~ in Jaboticabal, Brazil.** ~~Arrows indicate the nitrogen application (150 kg N ha-1 yr-1, split in three equal applications) in the fertilized treatment.~~

## Treatments and experimental design

There were three treatments (~~sward~~ nitrogen management strategies, N~~S~~M) with four replications, arranged in a completely randomized design with plot size of 20 × 60 m (Fig 2). Treatments were C~~1) c~~ontrol~~–~~: ~~'~~Marandu~~'~~ palisadegrass in a monoculture, unfertilized (without N fertilizer or legume); F~~2) f~~ertilized: ~~–'~~Marandu~~'~~ palisadegrass fertilized with 150 kg N $ha^{-1}$ $year^{-1}$ (urea), split into three equal applications; and ~~3) m~~Mixed: ~~–~~ Marandu palisadegrass mixed with forage peanut cv. ~~'~~Amarillo~~'~~ and no N fertilizer~~, during the establishment period~~. These treatments were evaluated for two ~~grazing~~ rainy ~~trials~~ seasons, with five ~~grazing~~ growth cycles (GC) ~~evaluations~~within each season (Table 1)~~: 19-30 Nov. 2016 and 4-15 Dec. 2017 (GC1), 10-21 Dec. 2016 and 3-14 Jan. 2018 (GC2), 9-20 Jan. 2017 and 5-16 Feb. 2018 (GC3), 6-17 Feb. 2017 and 12-23 Mar. 2018 (GC4), and 13-24 Mar. 2017 and 23 Apr.-4 May 2018 (GC5)~~. Nitrogen was applied to the fertilized treatment in November, January, and March for each ~~grazing~~ rainy ~~trial~~ season (50 kg N $ha^{-1}$ per application).

**Fig 2. Schematic representation of the experimental design. Three treatments with four replications, arranged in a completely randomized design. Experimental unit of 20 × 60 m (1200 m$^2$).** Control: Marandu palisadegrass in a monoculture (without N fertilizer or legume); Fertilized: Marandu palisadegrass fertilized with 150 kg N ha$^{-1}$ year$^{-1}$ (urea split into three equal applications); and Mixed: Marandu palisadegrass mixed with forage peanut cv. Amarillo and no N fertilizer. Intermittent stocking method with grazing periods lasting 3 d, 1 d for each paddock. Palisadegrass pasture was used as a reserve area to keep the animals during the rest periods.

**Table 1. Date of experimental period during five growth cycles and for two growing seasons.**

| Growth cycle (GC) | Initial date | Final date | Rest period [a] days |
|---|---|---|---|
| | **Growing season (2016/2017)** | | |
| Staging grazing | 07 Sept 2016 | 18 Sept 2016 | - |
| GC1 | 19 Nov 2016 | 30 Nov 2016 | 62 |
| GC2 | 10 Dec 2016 | 21 Dec 2016 | 21 |
| GC3 | 09 Jan 2017 | 20 Jan 2017 | 30 |
| GC4 | 06 Feb 2017 | 17 Feb 2017 | 28 |
| GC5 | 13 Mar 2017 | 24 Mar 2017 | 35 |
| | **Growing season (2017/2018)** | | |
| Staging grazing | 07 Sept 2017 | 18 Sept 2017 | - |
| GC1 | 04 Dec 2017 | 15 Dec 2017 | 77 |
| GC2 | 03 Jan 2018 | 14 Jan 2018 | 30 |
| GC3 | 05 Feb 2018 | 16 Feb 2018 | 33 |
| GC4 | 12 Mar 2018 | 23 Mar 2018 | 35 |
| GC5 | 23 Apr 2018 | 04 May 2018 | 42 |

[a] The rest period was interrupted, and grazing for all treatments initiated when one of the treatments reached 95% light interception.

~~Forage peanut presents a slow establishment when mixed with aggressive-growing grasses, however, over time this legume is able to compete and enhance its contribution in mixed system [21]. The low presence of forage peanut during the establishment period occurred in the present study. Nevertheless, we decided to continue the experiment to test if a small amount of forage peanut mixture with grass resulted in any contribution to the mixed system.~~

## Plot establishment ~~and management~~

In March 2014, existent 'Tifton 85' bermudagrass (*Cynodon* spp.) was sprayed with 2880 g a.i. $ha^{-1}$ of glyphosate [isopropylamine salt of N- (phosphonomethyl) glycine] in order to eradicate the existing vegetation. In October 2014, glyphosate plus 402 g a.i. $ha^{-1}$ of 2,4-D amine (2,4-dichlorophenoxyacetic acid) were applied, and subsequently, a crusher and chopper (Tritton, model 1800) was used to remove dead residue. Grass monoculture was seeded subsequently with a row spacing of 0.45 m and a seeding rate of 10 kg $ha^{-1}$ of pure live seed using a no-till seed drill (Semeato, model SHM 11-13). In the mixed treatment, forage peanut was simultaneously sown between rows of palisadegrass at a seeding rate of 8 kg $ha^{-1}$ of pure live seeds using a seed drill (Jumil, model 2640 PD Exacta). After the seeding, all paddocks received 60 kg P, 20 kg S, 50 g Mo, 1 kg Cu and 1 kg B $ha^{-1}$, following recommendations of Werner [233]. At the beginning of the grazing trial in both years, single superphosphate (40 kg P $ha^{-1}$) and potassium chloride (50 kg K $ha^{-1}$) were applied. The grass-legume mixed pastures were not fertilized with N.

Before seeding in 2014, the three treatments were randomized and allocated into 12 experimental units (1200 $m^2$ each). Each experimental unit was split into three paddocks of 400 $m^2$.

## Grazing management

In September of each year, heifers were used to stage standardize the plots., and the paddocks were defoliated using mob stocking [24]. The grazing method was intermittent stocking with grazing periods lasting 3 d with 1 d for each paddock (Fig 2). The mob stocking technique was used for pasture defoliation [24] with a minimum of five crossbred dairy heifers (311 ± 6 and 234 ± 4 kg, 2016/2017 and 2017/2018, respectively) for grazing. Additional heifers were 'put-and-take' as needed to achieve the target stubble height of 15 cm within one

day of occupation of a given paddock. The same animal group was used for the three paddocks within an experimental unit.

Grazing for all treatments initiated when one of the treatments reached 95% light interception (LI) [2~~5~~5] determined using the canopy analyzer LAI-2200 (LI-COR®, USA). The average ~~of~~ light interception was 95, 96, and 90% for control, fertilized, and mixed treatments, respectively. ~~Usually, the first grazing cycle occurred approximately 40 d after start of the rainy season (November).~~ Five ~~grazing~~ growth cycles occurred in each ~~grazing~~ rainy ~~trial~~ season with a rest period (RP) between them varying from 21 to 42 d (Table 1), ~~because growth rates varied seasonally resulting in different amounts of time required to reach~~due to seasonal and annual growth variation for time to reach 95% LI. ~~Grazing periods lasted 3 d, being 1 d for each paddock. Grazing ended on a given paddock when a stubble height of 15 cm was reached. A minimum of five crossbred heifers (311 ± 27 and 234 ± 19 kg, 2016/2017 and 2017/2018, respectively) was used for grazing. Additional heifers were added as needed to achieve the target stubble height in 1 d of grazing on a given paddock. The same animal group was used for the three paddocks within an experimental unit.~~ A reserve palisadegrass pasture was used to keep the animals during the rest periods.

## Response variables

### Herbage and canopy characteristics ~~structures~~

All samples were collected ~~on~~ from the second paddock on the second grazing day (Fig 2). ~~Herbage accumulation rate [kg of dry mass (DM) $ha^{-1}$ $d^{-1}$] was estimated as the difference between the pre-grazing herbage mass of the current grazing cycle and the post-grazing herbage mass of the previous grazing cycle (leaf + stem + dead mass was considered in this calculation), divided by the number of rest days between grazing cycles.~~ Before putting the grazing animals

on paddocks, the canopy height was estimated using a sward stick at 16 random points. The average canopy height was calculated and two samples representing the average herbage mass were taken using 0.25-$m^2$ quadrats. The herbage biomass at pre-~~herbage~~ grazing ~~mass~~ was harvested at 2 cm from the ground level. A subsample taken from the ~~pre~~ herbage biomass (fertilized and control treatment) was separated into leaf blade (leaf biomass), stem plus sheath (stem biomass), and ~~dead~~ senescent ~~material~~biomass (brown leaf and stem attached to the plant). When present, the inflorescence was included in the stem fraction. For the ~~palisadegrass–forage peanut~~mixed treatment, the entire sample was hand separated to measure the proportion of legume and grass (botanical composition)~~, and their respective morphologic components (leaf, stem, and dead mass)~~. For calculation of ~~pre-grazing~~ herbage biomass at pre-grazing [~~(~~kg of dry mass (DM) $ha^{-1}$~~)~~], all aboveground biomass (leaf + stem + ~~dead~~ senescent biomass) was considered; for the mixed treatment, total grass plus legume was considered. ~~The green herbage mass (kg DM $ha^{-1}$) included the green leaf and stem without the dead mass.~~ These fractions and the remaining non-separated herbage were oven-dried at 55°C for 72 h to calculate leaf:stem ratio (L:S). Herbage accumulation rate (~~[~~kg ~~of dry mass (DM)~~ $ha^{-1}$ $d^{-1}$~~]~~) was estimated as the difference between the ~~pre-grazing~~ herbage biomass at pre-grazing of the current ~~grazing~~growth cycle and the ~~post-grazing~~ herbage biomass at post-grazing of the previous ~~grazing~~growth cycle (leaf + stem + ~~dead~~senescent biomass was considered in this calculation), divided by the number of rest days between ~~grazing~~growth cycles (Table 1). ~~Canopy bulk density was calculated by dividing pre-grazing herbage mass by canopy height (kg DM $ha^{-1}$ $cm^{-1}$).~~

## ~~Total aboveground nitrogen, nitrogen derived from the atmosphere, and biological $N_2$ fixation by forage peanut~~Nitrogen uptake, fixation, and allocation

At the beginning of each ~~grazing~~ growth cycle, samples of total aboveground biomass of forage peanut and non-$N_2$-fixing reference plants (weeds) including billygoat-weed (*Ageratum conyzoides* L.), beggar-ticks (*Bidens pilosa* L.), coat-buttons (*Tridax procumbens* L.), gomphrena-weed (*Gomphrena celosioides* Mart.), cockroach berry (*Solanum capsicoides* All.), and sanguinarea [*Alternanthera ficoidea* (L.) P. Beauv.] were collected from the same area. Different reference plants were collected due to the low number of weeds in the paddock. Samples were ground using a Wiley mill (Marconi, Ma680) to pass through a 1-mm stainless steel screen. After samples were roller milled for 16 h [26~~6~~], total N concentration, abundance of $^{15}N$ ~~and $^{13}C$~~ (‰) were analyzed using a mass spectrometer (model Delta V Advantage, Thermo Scientific) coupled to an elemental analyzer (model Flash 2000, Thermo Scientific) in the "John Day Stable Isotope Laboratory" at Embrapa Agrobiologia. The total aboveground N of forage peanut ($TAGN_{FP}$, kg N $ha^{-1}$) was calculated multiplying the N concentration by the herbage accumulation of forage peanut ~~($TAGN_{FP}$, kg N $ha^{-1}$) and palisadegrass ($TAGN_{PG}$, kg N $ha^{-1}$)~~. The percentage of N derived from the atmosphere (~~%~~Ndfa) for forage peanut was calculated using the equation described by Shearer and Kohl [27~~7~~]:

$$\sout{\%}Ndfa\ (\%) = \left(\frac{\delta^{15}N_{\mathrm{refence}} - \delta^{15}N_{legume}}{\delta^{15}N_{\mathrm{refence}} - \mathrm{B}}\right) \times 100$$

where $\delta^{15}N_{reference}$ is the $\delta^{15}N$ value detected in a reference plant (non-N fixing) growing in the same soil at the same ~~grazing~~ growth cycle in the palisadegrass-forage peanut paddock, $\delta^{15}N_{legume}$ is the $\delta^{15}N$ abundance of the forage peanut, and *B* is the $\delta^{15}N$ value of the N in the legume obtained from ~~dinitrogen~~ $N_2$ fixation. We used the *B* value of −1.35‰ reported by

Unkovich [288] for *Arachis hypogea* L. in Brazil. The $\delta^{15}N$ from reference plants ranged from 4.4 to 5.9‰ ± 0.6‰, with values changing with sampling date. The total contribution of N from biological $N_2$ fixation (-BNF) was calculated by multiplying $TAGN_{FP}$ by the %Ndfa [288].

## Statistical analysis

~~All ~~D~~d~~ata were analyzed using the MIXED procedure of the SAS statistical package (SAS Inst. Inc., Cary, NC, USA). Herbage biomass at pre-grazing, herbage accumulation rate, and morphological components of palisadegrass were analyzed with ~~For the analysis of variance of palisadegrass, SM~~N management, growth cycle~~GC~~ and their interaction ~~were ~~as fixed effects, whereas~~. The~~ paddock within N management and ~~(SM Y~~year~~)~~ were used as random effects. Morphological components of forage peanut, $TAGN_{FP}$, Ndfa, and BNF were analyzed with growth cycle as fixed effect and paddock within year as random effect. The ~~The GC were used as repeated measures. The Akaike information criterion (AIC) was used to choose the best covariance structure. ~~Satterthwaite approximation was used to determine the denominator degrees of freedom for the test of fixed effects. The LSMEANS were compared using the PDIFF procedure by Fisher's protected least significant difference (LSD). Differences were considered significant at $P \leq 0.1$~~ test at 5% probability~~. Pearson correlation were carried out to explored the relationship between growth cycle (GC) and rainfall using the CORR procedure of the SAS.~~For the analysis of variance of forage peanut, the GC was the fixed effect. The paddock (GC Year) were the random effects. The Satterthwaite approximation was used to determine the denominator degrees of freedom for the test of fixed effects. The LSMEANS were compared using the PDIFF procedure by Fisher's protected least significant difference (LSD) test at 5% probability.~~

# Results

Nitrogen fertilizer increased the herbage biomass compared with unfertilized palisadegrass and palisadegrass-forage peanut mixture, which did not differ from each other

($P$ = 0.028; Figure 3A). There was an approached difference in N management × growth cycle on the herbage accumulation rate ($P$ = 0.106; Figure 3B). The urea fertilization increased the herbage accumulation rate in the GC4 compared to control and mixed treatment. Regarding the GC effect within N management, no difference was found in the herbage accumulation rate among GC for the mixed system. The greatest herbage accumulation rate occurred in the GC2 for the control treatment and the GC4 for the fertilized treatment. There was a positive correlation between rainfall and herbage accumulation rate (r = 0.18, $P$ = 0.085). However, the correlation was weak.

**Fig 3. Herbage biomass (A) and herbage accumulation rate (B) of palisadegrass under three nitrogen management strategies (NM) for five growth cycles (mean ± SE bars).** Control: palisadegrass without N fertilizer or legume; Fertilized: palisadegrass fertilized with 150 kg N $ha^{-1}$ $year^{-1}$; and Mixed: palisadegrass mixed with forage peanut and no N fertilizer. [*]Growth cycle 1 (GC1) was not evaluated, because the stubble mass was not performed after the staging grazing cycle in September. [†]Non-significant. Data are means across 2 years, and 4 replicates (n = 8). Different lowercase letter is different according to the PDIFF procedure by LSD test ($P \leq 0.1$).

The mixed treatment was separated into palisadegrass and forage peanut to compare the morphological components of palisadegrass among treatments (Table 2). Canopy height, leaf biomass, and stem biomass of palisadegrass at the pre-grazing event were affected by NM and GC, while the L:S ratio was only affected by GC ($P \leq 0.1$). Nitrogen fertilization increased canopy height, leaf biomass, and stem biomass of palisadegrass compared with the control and mixed treatments. Canopy height, stem biomass, and L:S ratio of palisadegrass was similar

among GC, except in GC5, which had the lowest canopy height. In contrast, the lowest stem biomass, and the greatest L:S ratio occurred in GC1. The greatest leaf biomass of palisadegrass was observed in GC1, while the lowest mean occurred in GC5. There was a positive correlation between rainfall and canopy height ($r = 0.23$, $P = 0.013$), leaf biomass ($r = 0.31$, $P < 0.001$), and L:S ratio ($r = 0.17$, $P = 0.064$).

**Table 1. Morphological components of palisadegrass under three nitrogen management strategies, and forage peanut biomass and nitrogen fixation of forage peanut in the mixed system, for two rainy seasons conducted during five growth cycles.**

| | Nitrogen management strategies (NM) | | | SE | Growth cycle (GC) | | | | | SE | *P*-value | | |
|---|---|---|---|---|---|---|---|---|---|---|---|---|---|
| | Control | Fertilized | Mixed[a] | | 1 | 2 | 3 | 4 | 5 | | NM | GC | NM×GC |
| *Grass* | | | | | | | | | | | | | |
| CH | 26b | 38a | 27b | 3 | 33a | 31a | 32a | 31a | 25b | 2 | 0.023 | 0.002 | 0.228 |
| Leaf | 1700b | 2490a | 1310c | 130 | 2230a | 1670bc | 1910b | 1970ab | 1390c | 130 | <0.001 | <0.001 | 0.567 |
| Stem | 1330ab | 1970a | 1070b | 280 | 1050b | 1510a | 1450a | 1750a | 1520a | 210 | 0.083 | 0.032 | 0.669 |
| Senescent | 1360 | 1390 | 1220 | 140 | 1010c | 1360b | 1220bc | 1270bc | 1940a | 140 | 0.942 | <0.001 | <0.001 |
| L:S ratio | 1.7 | 1.5 | 1.8 | 0.2 | 2.3a | 1.5b | 1.6b | 1.5b | 1.6b | 0.2 | 0.679 | <0.001 | 0.672 |
| *Legume* | | | | | | | | | | | | | |
| CH | - | - | 7.4 | 1.3 | 8.7a | 7.8ab | 8.3a | 8.0a | 4.2b | 1.3 | - | 0.104 | - |
| FP | - | - | 239 | 126 | 263a | 321a | 304a | 249a | 60b | 126 | - | 0.143 | - |
| L:S ratio | - | - | 1.1 | 0.2 | 1.4a | 1.2abc | 1.3ab | 0.9bc | 0.8c | 0.2 | - | 0.092 | - |
| $TAGN_{FP}$ | - | - | 3.1 | 2.5 | 6.8a | 1.9b | 4.1ab | 2.1b | 0.6b | 2.5 | - | 0.099 | - |
| Ndfa | - | - | 82 | 2 | 79c | 81bc | 82ab | 82b | 85a | 2 | - | 0.029 | - |
| BNF | - | - | 2.5 | 2.0 | 5.5a | 1.5ab | 3.2a | 1.7ab | 0.5b | 2.0 | - | 0.010 | - |

[a] Mixed treatment was split in palisadegrass and forage peanut data.

BNF, biological $N_2$ fixation (kg N $ha^{-1}$ $cycle^{-1}$); CH, canopy height (cm); FP, forage peanut biomass (kg DM $ha^{-1}$); leaf biomass (kg DM $ha^{-1}$); L:S, leaf: stem ratio; Ndfa, nitrogen derived from the atmosphere by forage peanut (%); SE, standard error; stem biomass (kg DM $ha^{-1}$); senescent biomass (kg DM $ha^{-1}$); $TAGN_{FP}$, total aboveground N of forage peanut (kg N $ha^{-1}$ $cycle^{-1}$).

Senescent biomass was affected by NM × GC interaction ($P$ < 0.001; Fig 4). The senescent biomass varied with N management strategies during the growth cycles. The N fertilization resulted in less senescent biomass in GC1 and GC2 compared with control and mixed treatment, while an increase was observed in GC5 (Fig 4A). Regarding the GC effect within N management, the lowest senescent biomass was observed in the GC1, GC3, and GC4 for the fertilized, control, and mixed treatment, respectively (Fig 4B). The greatest senescent biomass was observed in GC2 and GC4 for the control treatment and in GC5 for fertilized and mixed treatments. There was a negative correlation between rainfall and senescent biomass (-0.28, $P$ = 0.003), i.e., a rainfall reduction increased the senescent biomass of the palisadegrass.

**Fig 4. –Nitrogen management strategies (NM) × growth cycle (GC) interaction effect on senescent biomass of palisadegrass (mean ± SE bars).** Control: palisadegrass without N fertilizer or legume; Fertilized: palisadegrass fertilized with 150 kg N $ha^{-1}$ $year^{-1}$; and Mixed: palisadegrass mixed with forage peanut and no N fertilizer. †Non-significant. Data are means across 2 years, and 4 replicates (n = 8). Different lowercase letter within NM are different according to the PDIFF procedure by LSD test ($P \leq 0.1$).

Forage peanut was separated from the palisadegrass in the mixed treatment to evaluate their pattern across the growth cycles (Table 2). There was an approached difference of forage peanut biomass across growth cycle ($P$ = 0.143). Forage peanut canopy height, L:S ratio, total aboveground N of forage peanut ($TAGN_{FP}$), and biological $N_2$ fixation (BNF) showed a reduction mainly in the GC5 ($P \leq 0.1$), while nitrogen derived from the atmosphere (Ndfa) increased in the same cycle. There was a positive correlation between rainfall and canopy height (r = 0.41, $P$ = 0.017) and L:S ratio (r = 0.54, $P$ = 0.001). The botanical composition of

forage peanut and palisadegrass in the mixed treatment differed among GC ($P$ < 0.001), however, the proportions did not change, except in GC5, which had the lowest forage peanut proportion (2% of the botanical composition). The proportion of forage peanut among GC1 to GC4, ranged between 7 to 9% of the botanical composition.

# ~~Results and d~~Discussion

## ~~Herbage structures~~

The goal of our study was to test whether there was a meaningful and measurable contribution of the N from forage peanut even at minimal participation in the mixed system. ~~Nitrogen fertilizer enhanced pre-grazing herbage mass by 25 and 33% (P = 0.03) and green herbage mass by 32 and 41% (P = 0.01), compared with unfertilized palisadegrass and palisadegrass-forage peanut mixture, respectively (Table 1).~~ Nitrogen is generally the most limiting nutrient for plant growth, enhances tillering [2~~9~~9], leaf number and/or leaf elongation [30], and is a major component of the chlorophyll molecule [3~~0~~1]. Results of studies that accounted for the capacity of forage peanut to fix the nitrogen derived from atmospheric (Ndfa) were consistent with our findings. Assessments developed in the Southeastern United States [12, 32] and Northeast and Southeastern Brazil [14, 33] have shown that the %Ndfa for rhizome peanut and forage peanut can vary between 60 to 96% regardless of being in monocultures or mixtures. However, the $N_2$ input by the forage peanut in our study was insufficient to promote palisadegrass growth beyond what was found under unfertilized palisadegrass management or close to synthetic N fertilized systems.

In the present study, the total amount of $N_2$ fixation was 15.5 kg N $ha^{-1}$ for five growth cycles. The weather and soil conditions are considered key drivers that regulate the biological

**Formatted:** Font: Not Italic

**Formatted:** Font: Not Italic

nitrogen fixation (BNF), mainly by affecting the bacterial activities, which are responsible for fixing the $N_2$ [34]. However, no correlation between rainfall and BNF was found in the present study (r = 0.26, *P* = 0.128). Another assumption of these relatively low BNF was due to the reduced participation of forage peanut in the botanical composition (< 10%) in the mixed system. The legume proportion between 20 - 45% in the herbage biomass was considered sufficient to supply N to the agroecosystem [19], fixing between 50 - 100 kg N $ha^{-1}$ $yr^{-1}$ [35].

On the other hand, the N supply through the synthetic fertilizer enhanced herbage biomass by 33 and 49% and leaf biomass by 46 and 90% of palisadegrass compared with unfertilized palisadegrass and palisadegrass-forage peanut mixture, respectively (Fig 3; Table 2). The N fertilization increases the cells production, which increases leaf elongation [36] and regeneration of the tiller population, by increasing tiller appearance rate and tiller death; these factors can vary with season due to the weather conditions, such as temperature and rainfall [37]. In addition, the N fertilized treatment always reached the 95% light interception threshold before the other treatments. Therefore, the low N treatments (grass or mixture) did not grow as much over the same amount of time as the N fertilized grass, which was reflected in herbage biomass and related measurements.

The extra N may have contributed to competition for light among tillers, and the plant elongates the stem under these conditions in search of the light for the younger leaves [30]. As part of a canopy sward, there is no benefit for a plant to create relatively more leaf biomass when it cannot intercept anymore light than it already has [38], which may explain the greatest stem proportion and canopy height in the N fertilized treatment without effect on the L:S ratio among N management strategies. In the present study, the rainfall was positively correlated with canopy height (r = 0.23, *P* = 0.013), leaf biomass (r = 0.31, *P* < 0.001), and L:S ratio (r = 0.17, *P* = 0.064) of palisadegrass. Stem elongation is not desirable for grazing animal nutrition,

basically for three reasons. First, increasing the stem can reduce the leaf proportion [39]; second, the stem elongation increases the canopy height, which will affect the ingestive animal behavior, such as reducing the number of tongue sweeps, bite volume, and bite weight [39, 40]; and third, the stem acts as a support structure of leaves, which has secondary cell walls, such as lignin, which are undigestible components [41, 42]. These factors can limit the selection of leaf and, consequently, reduces animal gain [43].

Nitrogen fertilization combined with favorable weather conditions promoted greater green biomass (leaf and stem) and reduced the senescent biomass, while the N fertilization and drought conditions of the transition period between rainy to dry season may have increased the senescent biomass. A negative correlation occurred between senescent biomass and rainfall (-0.28, $P$ = 0.003). The nitrogen fertilization associated with rainfall reduction may have affected maturity stage in the last GC and may have hastened flowering stem elongation and leaf senescent compared to other treatments. The N fertilization reduce the leaf lifespan, which increase the senescent biomass compared with unfertilized grass [44].

Under regular weather conditions (rainfall, temperature, and photoperiod), warm-season $C_4$ grasses are typically more competitive than legumes, shifting the balance in mixed swards towards the grass [14]. This competition can reduce the persistence of forage peanut in mixed systems. Grass establishment, growth, and time to recover after grazing tend to be faster than for legumes [10]. Our study evidenced a reduction in the presence of forage peanut due to drought conditions during the transition period of the rainy to dry season. During the dry season, leaf loss typically occurs for forage peanut [45]. In Northwest Brazil, [17] reported that the drought stress reduced the forage peanut biomass in the mixed system during the dry season. On the other hand, in Northeast Brazil, [46] did not find a negative impact of the dry season on the forage peanut growth. The authors suggested positive legume performance due to the

favorable climatic weather in the study region, even during the dry season. Thus, differences among Brazilian regions, weather conditions, and latitude play an important role in the forage peanut growth, mainly in the dry season. With the onset of the rainy season, forage peanut initiates growth. Remarkably, the transition period between dry to rainy season plays an important role in the forage peanut recovery due to the low grass canopy height and biomass and bare ground space for emission of stolons and germination of soil seed bank after the first rains [17, 47], however, this recovery occurs slowly [48].

Forage peanut presents a slow establishment when mixed with aggressive-growing grasses such as palisadegrass, however, over time this legume was able to compete and enhance its contribution in mixed system [49]. Besides, *Arachis pintoi* has great tolerance to grazing, mainly because the growing points are protected from grazing animals, unlike most other tropical legume species. Thus, it is possible to maintain residual leaf area and initial growth even when the plant is subjected to continuous stocking and intense defoliation [50]. Pereira [46] reported that 20.5 to 23.8% of forage peanut mixed with palisadegrass resulted in a liveweight gain per ha 29% greater than in N fertilized system (dose of 120 kg N $ha^{-1}$). Therefore, if our study had addressed nutritive value and animal performance, perhaps we would have found the mixed system benefits even at minimal legume participation.

Keeping the light interception between 90 and 95% with canopy height ranging from 26 to 32 cm for palisadegrass-forage peanut mixtures was considered the best grazing management to provide greater herbage accumulation rate, especially leaves [25]. However, in our study with a minimal proportion of forage peanut, it is necessary to keep the LI less than 90%. Canopy height control in mixed systems is important because two different plant species compete for light, water, and nutrient in the same area [51]. Studies have been shown that a canopy height of 20 cm as the optimum to guarantee the best forage peanut proportion, mainly

in the first years after seeding without compromising the grass growth [15, 52]. The increase in grass canopy height can stimulate vertical growth of forage peanut, reducing the number of stolons and decreasing legume proportion in the mixed system. These changes in growth habit of forage peanut may limit its ability to compete with palisadegrass in mixed stands. In the current study, high canopy height might have delayed the full establishment of forage peanut in the mixed system, with their growth being limited by shading from palisadegrass [15].

Nutrient competition between grass and legume, the lack of N fertilization in the first years after seeding of the mixed system, and the slow establishment of forage peanut may have resulted in low herbage biomass of both legume and grass in the mixed system. During the first years after seeding of grass-legume mixed pastures, N fertilization could be used, considering that legumes have limited capability to fix atmospheric $N_2$ during initial developmental stages [35]. Further studies are required to evaluate whether the N fertilization could hasten the legume proportion and provide nutrients to sustain grass growth, at least in the mixed system initial years. The ultimate goal is to develop a sustainable mixed system that provides quality feed to the grazing animal, reflecting on the cost reduction to the farmer and the environmental impacts of synthetic N fertilizers. Therefore, further studies are required to test whether there is a meaningful and measurable contribution of the forage peanut on nutritional value and animal performance, even at minimal legume participation.

~~. In similar conditions to those of this study, Delevatti [4] reported an increasing linear effect of N fertilization (0, 90, 180, and 270 kg N ha$^{-1}$ year$^{-1}$) on palisadegrass herbage mass. In the Brazilian Cerrado, N fertilization (100 kg N ha$^{-1}$) increased palisadegrass herbage accumulation by 22% compared with unfertilized palisadegrass [31]. There was no difference for pre-grazing herbage mass and green herbage mass between the unfertilized palisadegrass~~

~~and palisadegrass-forage peanut mixture, indicating that inclusion of the legume in the system did not negatively affect palisadegrass growth. We hypothesized that forage peanut could fix atmospheric N and supply N to palisadegrass. However, the N input by the forage peanut in our study was not sufficient to promote growth of palisadegrass in the mixed system similar to the N-fertilized system.~~

~~**Table 1. Pre-grazing herbage mass (PHM), green herbage mass (GHM), and herbage accumulation rate (HAR) of palisadegrass in monoculture, palisadegrass fertilized with 150 kg N ha$^{-1}$ (split in three equal applications), or during the establishment of palisadegrass-forage peanut mixtures, for two grazing trials conducted during five grazing cycles.**~~

| | Sward management strategies (SM) | | | | Grazing cycle (GC) | | | | | | P-value | | |
|---|---|---|---|---|---|---|---|---|---|---|---|---|---|
| | ontr ol | ertili zed | ixed ‡ | E | | | | | | E | M | C | M× GC |
| P HM, kg DM ha$^{-1}$ | 400a b† | 830a | 880 b | 90 | 380 | 460 | 650 | 060 | 960 | 00 | .03 | .66 | .52 |
| G HM, kg DM ha$^{-1}$ | 030 b | 440a | 600 b | 00 | 370 | 190 | 450 | 770 | 020 | 30 | .01 | .19 | .83 |
| H AR, kg | 7 | 3 | 1 | 4 | A | 1a | 6ab | 8a | 2b | 3 | .32 | .04 | .10 |

**Formatted:** Justified, Indent: First line: 0.5", Line spacing: Double

**Formatted:** Justified, Indent: First line: 0.5", Line spacing: Double

**Formatted:** Justified, Indent: First line: 0.5", Line spacing: Double

**Formatted:** Justified, Indent: First line: 0.5", Line spacing: Double

**Formatted:** Justified, Indent: First line: 0.5", Line spacing: Double

| | | | | | | | | | | | | | |
|---|---|---|---|---|---|---|---|---|---|---|---|---|---|
| DM ha$^{-1}$ d$^{-1}$ | | | | | | | | | | | | | |
| Leaf:stem ratio | .7 | .5 | .7 | .2 | .2a | .4b | .5b | .5b | .5b | .1 | .78 | .01 | .15 |

Formatted: Justified, Indent: First line: 0.5", Line spacing: Double

† Means not followed by the same letter are different according to the PDIFF procedure by LSD test ($P \leq 0.05$).

Formatted: Font: Not Italic

‡ Values of palisadegrass with forage peanut.

NA, not applicable (The GC1 was not evaluated, because the stubble mass after the standardization grazing in September was not performed).

SE, Standard error.

There was no difference between SM for herbage accumulation rate, however, GC influenced the response (Table 1). Ribeiro [32] reported a greater herbage accumulation rate when a bermudagrass-forage peanut mixture was fertilized with N compared with an unfertilized mixture; however, the herbage accumulation rates were similar when 100 or 200 kg N ha$^{-1}$ was applied in the mixed systems. The authors suggested the dry season affected the legume recovery in the rainy season. In our study, the forage peanut proportion decreased in the last grazing cycle, by the drought conditions at the beginning of the transition of rainy season to dry season. Although we did not evaluate responses during the dry season, we noticed that the low rainfall during the dry season affected the presence of forage peanut. When the rainy season began, the forage peanut initiated growth, however, its recovery was slow. This may partially explain the lesser response of mixed systems in our study. In the same region, Delevatti [4] found a linear effect of N fertilizer rate on herbage accumulation rate of

palisadegrass. However, in our study, the herbage accumulation rate of the N-fertilized treatment did not differ from mixed or unfertilized systems (Table 1). Sanchez [33] did not find difference in the herbage accumulation rate using bermudagrass in a monoculture or mixed with forage peanut (28 vs. 19 kg $ha^{-1}$ $d^{-1}$), however, the authors observed difference between months due to limited rainfall and temperature. The weather conditions in different GC affected herbage accumulation. The values decreased from 81 to 52 kg DM $ha^{-1}$ $d^{-1}$ from GC2 to GC5, respectively. Average rainfall during the rest period between GC was 95, 60, 116, and 42 mm (for GC1 to GC2, GC2 to GC3, GC3 to GC4, and GC4 to GC5, respectively). Probably this pattern contributed to increasing the herbage accumulation rate in the GC2 and GC4, whereas no diff erences were observed in GC3 and GC5 due to less favorable temperature and rainfall (Fig 1).

There was no difference between SM for L:S ratio of green herbage mass, however, GC influenced the response ($P < 0.01$; Table 1). Greater L:S ratio was observed only in the GC1. The experimental period started two months after the grazing standardization, when the treatments reached 95% light interception. Probably the two months of rest period resulted in greater leaf mass. When the grazing cycle started, the rainfall increased, and the rest period reduced (~32 days), resulting in low leaf mass. There was a reduction of the L:S ratio after the GC1. It was expected that N-fertilized grasses would show greater L:S ratio by the increase in the leaf number and/or leaf elongation [34]. However, the N fertilization increased the leaf proportion as well as the stem proportion. Alencar [14] did not find a difference for L:S ratio for the palisadegrass in a mixture or a monoculture. Similar results were found in this study.

## Morphological components of palisadegrass

Canopy height, leaf mass, and stem mass of palisadegrass at pre-grazing event were affected by SM and GC (*P* ≤ 0.05; Table 2). Canopy height was greater in the N fertilized treatment (38 cm) than in the control (26 cm) and in the mixed sward (27 cm). Canopy height at 95% light interception was not constant for the SM studied. Therefore, canopy height can be a better strategy to compare monoculture and mixed pastures in future studies, at lower cost. Pereira [25] reported light interception between 90 and 95% with canopy height ranging from 26 to 32 cm for palisadegrass-forage peanut mixtures as the best grazing management to provide greater herbage accumulation rate, especially leaves. Brougham [35] indicated that 90 to 95% light interception leads to greatest herbage accumulation rates, especially in monoculture or mixed pastures. Canopy height control in mixed systems is important because there are two different plant species competing for light in the same area. Tamele [15] in a harvesting study (weekly harvests) reported proportions of forage peanut ranging from 11 to 17% when the canopy height of palisadegrass was 30 cm; the optimum canopy height to increase the proportion of forage peanut was 20 cm. In the current study, the increase of canopy height might have decreased the proportion of legume, leaf mass, and subsequently $TAGN_{FP}$ and BNF. In pastures sampled seven years after planting, Gomes [36] found canopy height of palisadegrass in mixed system ranging between 25 (for 90% LI) and 30 cm (for 95% LI) without inhibiting the growth of the forage peanut in rotational stocking. Canopy height decreased in the GC5. This response may have occurred by the drought conditions at the beginning of the transition season (rainy to dry season), resulting in low green herbage mass (leaf and stem) and great dead mass in the GC5. This variation in canopy height suggests that there was no correlation between canopy height and light interception with the change from the rainy to the dry season. Canopy height is affected by management strategy, forage species, and season; therefore, the same LI may result in different canopy heights, mainly due to

~~changes in plant architecture resulted from change in those factors. In our study, we used 95% LI target to start the grazing period, and the canopy height changed among GC (ranged from 26 to 33 cm). Pereira [25] reported similar results among evaluation period of palisadegrass-forage peanut (26 to 33 cm).~~

~~**Table 2. Canopy height (CH), leaf herbage mass (LHM), stem herbage mass (SHM), and canopy bulk density (CD) of palisadegrass in monoculture, palisadegrass fertilized with 150 kg N ha$^{-1}$ (split in three equal applications), or during the establishment of palisadegrass-forage peanut mixtures, for two grazing trials conducted during five grazing cycles.**~~

| | ~~Sward management strategies (SM)~~ | | | ~~E~~ | ~~Grazing cycles (GC)~~ | | | | | ~~E~~ | ~~*P* value~~ | | |
|---|---|---|---|---|---|---|---|---|---|---|---|---|---|
| | ~~ontr ol~~ | ~~ertili zed~~ | ~~ixe d‡~~ | | | | | | | | ~~M~~ | ~~C~~ | ~~M× GC~~ |
| ~~C H, cm~~ | ~~6b‡~~ | ~~8a~~ | ~~7b~~ | | ~~3a~~ | ~~0a~~ | ~~2a~~ | ~~1a~~ | ~~6b~~ | | ~~.02~~ | ~~.01~~ | ~~.08~~ |
| ~~L HM, kg DM ha$^{-1}$~~ | ~~700 b~~ | ~~470a~~ | ~~310 b~~ | ~~30~~ | ~~230 a~~ | ~~670 bc~~ | ~~910 ab~~ | ~~950 ab~~ | ~~390 c~~ | ~~30~~ | ~~.01~~ | ~~.01~~ | ~~.62~~ |
| ~~S HM, kg DM ha$^{-1}$~~ | ~~350 ab~~ | ~~960a~~ | ~~020 b~~ | ~~70~~ | ~~090 b~~ | ~~320 ab~~ | ~~510 ab~~ | ~~770 a~~ | ~~540 ab~~ | ~~10~~ | ~~.05~~ | ~~.03~~ | ~~.79~~ |
| ~~C D, kg DM ha$^{-1}$ cm$^{-1}$~~ | ~~70~~ | ~~60~~ | ~~38~~ | ~~0~~ | ~~36c~~ | ~~54a bc~~ | ~~43b c~~ | ~~67a b~~ | ~~80a~~ | ~~1~~ | ~~.07~~ | ~~.02~~ | ~~.16~~ |

~~† Means not followed by the same letter are different according to the PDIFF procedure adjusted by LSD test ($P \leq 0.05$).~~

~~‡ Values for only palisadegrass (without forage peanut).~~

~~SE, Standard error.~~

**Formatted:** Justified, Indent: First line: 0.5"

**Formatted Table**

**Formatted:** Justified, Indent: First line: 0.5"

**Formatted:** Justified, Indent: First line: 0.5"

**Formatted:** Justified, Indent: First line: 0.5"

**Formatted:** Justified, Indent: First line: 0.5"

**Formatted:** Justified, Indent: First line: 0.5"

Leaf mass of palisadegrass for the N-fertilized treatment was greater than the control and mixed (2470 vs. 1700 and 1310 kg DM ha$^{-1}$, respectively). The N is linked with plant growth and tissue turnover. An explanation is N fertilization may increase the leaf number and/or leaf elongation [34]. Pereira [37] reported increases of 25% in leaf elongation rate in palisadegrass fertilized with 150 kg N ha$^{-1}$ when compared to unfertilized pastures during the summer. Fagundes [38] found a linear increase in leaf elongation rate for palisadegrass as N rates increased (75, 150, 225 and 300 kg N ha$^{-1}$ yr$^{-1}$). Silva [1] reported that N fertilization from 50 to 250 kg N ha$^{-1}$ yr$^{-1}$ resulted in an increase in leaf proportion of Mulato II brachiariagrass hybrid (*U. ruziziensis* × *U. decumbens* × *U. brizantha*) from 22 to 25%. Leaf mass was greatest in GC1 and lowest in GC5 (2230 vs. 1390 kg DM ha$^{-1}$, respectively). The experimental period started two months after the grazing standardization. Probably this rest period resulted in greater leaf mass. When the grazing cycles started, the rainfall increased, and the rest period reduced (~32 days), resulting in low leaf mass (Table 2). The shortest rest period was between GC1 and GC2 (21 and 30 days), probably it was responsible for the low leaf mass in the GC2, whereas the GC5 increased the rest period (35 and 42 days) due to limited rainfall, resulting in great dead mass and low leaf mass.

Palisadegrass stem mass for the N fertilized treatment was greater than the mixed treatment (1960 vs. 1020 kg DM ha$^{-1}$, respectively), which did not differ from the control treatment (1350 kg DM ha$^{-1}$). Nitrogen fertilization might have enhanced regeneration of the tiller population by increasing tiller appearance rate and tiller death; those factors can vary with season due to the weather conditions, such as temperature and rainfall [39]. In addition, the N fertilized treatment always reached first the 95% light interception than the other treatments, and this may have resulted in a competition for light between tillers, the plant will elongate the stem, especially in search of luminosity for the younger leaves [34]. The lowest stem mass was in GC1 and the greatest in GC4 (1090 vs. 1770 kg DM ha$^{-1}$). Average rainfall during the rest

**Formatted:** Justified, Indent: First line: 0.5"

period between GC3 and GC4 was 116 mm. During the rainy season, the tiller may have increased the population or elongation or become heavier to sustain more weight [34]. The stem is an important fraction for the tropical grasses because it is responsible for plant growth and leaf support to enhance light interception [38].

Canopy bulk density varied with GC (*P* = 0.02; Table 2) for palisadegrass, means ranged from 136 to 180 kg DM ha$^{-1}$ cm$^{-1}$. The lowest means were observed in GC1, and the greatest was in GC5. These differences are reflections of the herbage mass and the canopy height promoted by stem elongation of the palisadegrass during the GC, which are influenced by the weather conditions (Fig 1). As rainfall decreased, the stem proportion increased and consequently canopy bulk density increased. Many elements may influence the canopy bulk density including plant species ($C_4$ and $C_3$), pasture management (defoliation intensity), and weather conditions [39].

There was a SM × GC interaction on the dead herbage mass of palisadegrass ($P < 0.01$; Fig 2). Within of GC, there was difference between SM in the CG2 and GC5 (Fig 2A). The N fertilization reduced the dead herbage mass in the GC2, however, in the GC5 the N fertilized increased the herbage dead mass. The mixed and control treatments were not different from each other in both GC. Within of SM, there was difference between GC for the control, fertilized, and mixed treatments (Fig 2B). Lower dead herbage mass was observed in the GC1, GC3, and GC4 for the fertilized, control, and mixed treatment, respectively. Whereas, greater dead herbage mass was observed in the GC2 and GC4 for the control treatment, and in the GC5 for the fertilized and mixed treatments. Dead herbage mass of palisadegrass ranged from 990 to 1590 kg DM ha$^{-1}$, 640 to 2670 kg DM ha$^{-1}$, and 1010 to 1700 kg DM ha$^{-1}$ for control, fertilized, and mixed treatment, respectively. These responses may have occurred because the N fertilizer was applied in November, January, and March. The N fertilization combined with favorable weather conditions (temperature and rainfall) likely promoted greater green mass and

reduced the dead herbage mass in the GC2, while the N fertilization in March and drought conditions at the beginning of the transition season may have increased the dead herbage mass in the GC5. Nitrogen fertilization likely affected maturity stage in the last GC period, and may have hastened flowering stem elongation and leaf death compared to other treatments. Nitrogen fertilization might have enhanced regeneration of the tiller population by increasing tiller appearance rate and tiller death; those factors can vary with season due to the weather conditions, such as temperature and rainfall [39]. Pereira [37] reported increases of 22% in leaf appearance rate in palisadegrass fertilized with 150 kg N $ha^{-1}$ when compared with unfertilized pastures during the summer. This acceleration in leaf appearance rate may be responsible for increasing the dead material later in the season in our study. In a tropical environment, Gomes [36] reported that dead herbage mass for palisadegrass-forage peanut mixtures did not change between grazing frequencies (initiated at 100, 95, and 90% LI or grazed every 42 d) and seasons, with an average of 2332 kg $ha^{-1}$. Our study showed lower dead mass for the mixed system, and there was a difference in the variable among CG, and it was affected mainly by the weather conditions and soil nutrients. Similar pattern occurred to control treatment.

**Fig 2. Sward management strategies (SM) × grazing cycle (GC) interaction effect on dead herbage mass (mean ± SE bars) of palisadegrass during the establishment period.** ‡Non-significant. Data are means across 2 yr, and 4 replicates (n = 8). Control (palisadegrass without nitrogen fertilizer or legume), Fertilized (palisadegrass fertilized with 150 kg N $ha^{-1}$, split in three equal applications), or Mixed (palisadegrass-forage peanut mixtures no N fertilizer). Different lowercase letter within GC (A) and SM (B) are different according to the PDIFF procedure by LSD test ($P \leq 0.05$). Sward management strategies: $P = 0.95$, Grazing cycle: $P < 0.01$, Sward management strategies × Grazing cycle: $P < 0.01$.

## Morphological components of forage peanut

**Formatted:** Font: Italic

**Formatted:** Indent: First line: 0.5"

~~There were differences between GC for leaf and stem mass, but no differences were found for the dead mass of forage peanut. The dead herbage mass was consistently low, ranging from 4 to 22 kg DM ha$^{-1}$ (*P* = 0.26; Table 3). In the dry season, leaf loss typically occurs for forage peanut [36], whereas during the rainy season there are greater growth rates due to greater leaf appearance rate and elongation [25], with less death occurring. In this study, the leaf mass of forage peanut ranged from 21 to 197 kg DM ha$^{-1}$ (*P* = 0.03; Table 3), with the lowest value found in GC5.~~

~~**Table 3. Canopy height (CH), leaf herbage mass (LHM), stem herbage mass (SHM), dead herbage mass (DHM), and canopy bulk density (CD) during the establishment of forage peanut in a mixed system. The response variables are means across four replicates, and 2 yr (n = 8).**~~

| | Grazing Cycle (GC) | | | | | [illegible] | *P*- |
|---|---|---|---|---|---|---|---|
| | 1 | 2 | 3 | 4 | 5 | E | value |
| CH, cm | 9 | 8 | 9 | 8 | 6 | [illegible] | 0. |
| | | | | | | 48 | |
| LHM, kg | 1 | 1 | 1 | 1 | 2 | [illegible] | 0. |
| DM ha$^{-1}$ | 52a‡ | 97a | 53a | 11a | 1b | 0 | 03 |
| SHM, kg | 1 | 1 | 1 | 1 | 2 | [illegible] | 0. |
| DM ha$^{-1}$ | 07a | 81a | 29a | 29a | 0b | 5 | 05 |
| DHM, kg | 4 | 1 | 2 | 9 | 1 | [illegible] | 0. |
| DM ha$^{-1}$ | 2 | 2 | | 0 | | 26 | |
| CD, kg | 2 | 4 | 2 | 2 | 1 | [illegible] | 0. |
| DM ha$^{-1}$ cm$^{-1}$ | 7 | 4 | 9 | 7 | 1 | 0 | 39 |

**Formatted:** Justified, Indent: First line: 0.5", Line spacing: Double

**Formatted:** Justified, Indent: First line: 0.5", Line spacing: Double

**Formatted:** Justified, Indent: First line: 0.5", Line spacing: Double

**Formatted:** Justified, Indent: First line: 0.5", Line spacing: Double

**Formatted:** Justified, Indent: First line: 0.5", Line spacing: Double

**Formatted:** Justified, Indent: First line: 0.5", Line spacing: Double

‡ Means not followed by the same letter are different according to the PDIFF procedure by LSD test (P ≤ 0.05).

SE, Standard error.

Stem mass of forage peanut ranged from 20 to 181 kg DM ha$^{-1}$ (*P* ≤ 0.05; Table 3). Gomes [36] reported that stem mass for forage peanut did not change with grazing frequency treatments or seasons. Our study showed a reduction in stem mass in GC5, probably due to rainfall reduction and the beginning of the dry season. In these GC in southeastern Brazil the transition period between the rainy and dry season begins.

Our values are lower (113 vs. 766 kg ha$^{-1}$, respectively) than those reported by Gomes [36]. However, their pasture was established 7 yr before the experimental period. In this study, the forage peanut was not fully established yet because the experimental period began 2 yr after seeding. Pre-grazing herbage mass of forage peanut ranged from 249 to 390 kg DM ha$^{-1}$, decreasing in the GC5 to 51 kg DM ha$^{-1}$. Pereira [41] found 493 kg DM ha$^{-1}$ of forage peanut mixed with palisadegrass in the rainy season, and the mixed treatment resulted in a liveweight gain per ha of 28.7% greater than in the palisadegrass N fertilized with 120 kg N ha$^{-1}$.

There was no difference between GC for canopy height, and canopy bulk density of forage peanut (*P* > 0.05; Table 3). Canopy height ranged from 6 to 9 cm, and the canopy bulk density ranged from 17 to 44 kg DM ha$^{-1}$ cm$^{-1}$. Canopy bulk density of *Arachis glabrata* Benth. cultivars were influenced by cultivar, season, and grazing management [42]. High canopy bulk density may reduce the number of tongue sweeps, bite volume, and bite weight [43]. It can limit the selection of leaf and, consequently, reduces animal performance [44].

## Botanical composition

**Formatted:** Indent: First line: 0.5"

The botanical composition differed between GC ($P < 0.01$; Fig 3). Weed presence was minimal, and the data are not reported. The greatest proportion of palisadegrass (98%) was found in GC5, and the lowest proportion (91%) was found in GC2. The greatest proportion of forage peanut (9%) was found in GC2, and the lowest proportion (2%) was found in GC5. The botanical composition in GC1, GC3, and GC4 averaged 93 and 7% of palisadegrass and forage peanut, respectively. The presence of forage peanut in the mixed system declined as the rainy season ended. Warm-season $C_4$ grasses are typically more competitive than legumes, shifting the balance in mixed swards towards the grass [14]. This competition can reduce the persistence of forage peanut in mixed systems. Grass establishment, growth, and time to recover after grazing tend to be faster than for legumes [10]. In the Brazilian tropics, Tamele [15] reported a reduction in the proportion of forage peanut with increased canopy height (10, 20, 30, and 40 cm) of palisadegrass in a harvesting study (weekly harvests). The authors concluded that the canopy height of 20 cm provided the best proportion of legume (19 – 38%) in a mixed sward, without compromising the grass growth. The increase in grass canopy height can stimulate vertical growth of forage peanut, reducing the number of stolons and decreasing legume proportion in the mixed system. These changes in growth habit of forage peanut may limit its ability to compete with palisadegrass in mixed stands. In addition, the lower proportion of the legume in the botanical composition may be caused by the slow establishment [14]. Forage peanut growth may have been limited by shading from palisadegrass [15]. Santos [12] reported 57% rhizoma peanut in mixtures with bahiagrass 4 yr after seeding. It is possible that forage peanut available for use in Brazil is less vigorous during the establishment phase than rhizoma peanut. Studies in Florida USA show good results in the use of rhizoma peanut cultivars in mixed systems [12, 13]. *Arachis pintoi* has great tolerance to grazing, mainly because the growing points are protected from grazing animals, unlike most of the tropical legume species. Thus, it is possible to maintain residual leaf area even when the plant is subjected to continuous

stocking and intense defoliation [45]. Although the proportion of forage peanut in the botanical composition was lower than the expected to mixed systems, in our study, the legume proportion in the dung ranged from 11.2 to 15.3% (data not shown). Considering the forage peanut digestibility ranged from 68.0 to 68.7 [36], probably the legume proportion in the diet of heifers may have been greater than in the botanical composition.

**Fig 3. Effect of grazing cycle (GC) on botanical composition during the establishment of palisadegrass-forage peanut mixtures (*P* < 0.01).** Data are means across 2 yr, and 4 replicates (n = 8). Different lowercase letters are different between GC, according to the PDIFF procedure by LSD test (*P* ≤ 0.05).

## Total aboveground nitrogen, nitrogen derived from the atmosphere, and biological $N_2$ fixation by forage peanut

The total aboveground N of forage peanut ($TAGN_{FP}$) was affected by GC ($P \leq 0.05$; Table 4), ranging from 1 to 7 kg N ha$^{-1}$ cycle$^{-1}$. Greater N in herbage mass was present in GC1 and did not differ from GC2 and GC3. The low herbage accumulation of forage peanut resulted in low $TAGN_{FP}$. Leaf mass (Table 3) and presence of forage peanut in the botanical composition (Fig 3) were greater in GC1 to GC4. Total herbage accumulation of forage peanut for five GC was 613 kg DM ha$^{-1}$, resulting in a $TAGN_{FP}$ of 17 kg N ha$^{-1}$ (GC1 to GC5). The low herbage accumulation of forage peanut resulted in low $TAGN_{FP}$. The $TAGN_{FP}$ decreased when the forage peanut in the herbage accumulation decreased (GC4 and GC5). Jaramillo [13] reported values of 0.7 kg N ha$^{-1}$ harvest$^{-1}$ for forage peanut mixed with bahiagrass. Santos [12] reported TAGN for rhizoma peanut-bahiagrass mixtures of 50 kg N ha$^{-1}$. In our study, the forage peanut demonstrated low potential to compete with the palisadegrass, due to slow legume establishment. The nutrient competition between grass and legume, the lack of N

fertilization in the first years of establishment of the mixed system, and the slow establishment of forage peanut may have resulted in low herbage mass of forage peanut. During the establishment period of grass-legume mixed pastures, N fertilization can be used considering that legumes have limited capability to fix atmospheric $N_2$ during initial developmental stages [46]. Two weeks after germination, Alencar [14] fertilized a palisadegrass-forage peanut mixture with 30 kg N ha$^{-1}$, but the amount of N fixation was small (~ 4.0 kg ha$^{-1}$) due to the slow growth of the forage peanut.

**Table 4. Total aboveground N of palisadegrass (TAGN$_{PG}$) and forage peanut (TAGN$_{FP}$), nitrogen derived from the atmosphere (%Ndfa), and biological $N_2$ fixation (BNF) during the establishment of forage peanut in a mixed system. The response variables are means across four replicates, and 2 yr (n = 8).**

| | Grazing cycle | | | | | | |
|---|---|---|---|---|---|---|---|
| | **1** | **2** | **3** | **4** | **5** | **SE‡** | ***P*-value** |
| *Palisadegrass* | | | | | | | |
| TAGN$_{PG}$, kg N ha$^{-1}$ cycle$^{-1}$ | 31a† | 19b | 18b | 13b | 19ab | 4 | 0.05 |
| *Forage peanut* | | | | | | | |
| TAGN$_{FP}$, kg N ha$^{-1}$ cycle$^{-1}$ | 7a | 3ab | 4ab | 2b | 1b | 2 | 0.05 |
| %Ndfa | 79 | 81 | 82 | 82 | 85 | 2 | 0.14 |
| BNF, kg N ha$^{-1}$ cycle$^{-1}$ | 5.5a | 2.4ab | 3.2ab | 1.7b | 0.7b | 1.8 | 0.05 |

† Means not followed by the same letter are different according to the PDIFF procedure by LSD test ($P \leq 0.05$).

SE, Standard error.

The total aboveground N of palisadegrass (TAGN$_{PG}$) was affected by GC ($P$ = 0.04; Table 4). The TAGN$_{PG}$ ranged from 13 to 31 kg N ha$^{-1}$ cycle$^{-1}$. Greatest N aboveground was found in GC1 and the lowest in GC4. The difference in the rainfall and temperature (Fig 1) can

**Formatted:** Justified, Indent: First line: 0.5"

**Formatted:** Justified, Indent: First line: 0.5"
**Formatted:** Justified, Indent: First line: 0.5"
**Formatted:** Justified, Indent: First line: 0.5"
**Formatted:** Justified, Indent: First line: 0.5"
**Formatted:** Justified, Indent: First line: 0.5"
**Formatted:** Justified, Indent: First line: 0.5"
**Formatted:** Justified, Indent: First line: 0.5"

explain the differences found between GC. Weather conditions influence herbage accumulation and N concentration in the grass and legume. Total herbage accumulation of palisadegrass for five GC was 9785 kg DM ha$^{-1}$ (GC1 to GC5), resulting in a $TAGN_{PG}$ of 100 kg N ha$^{-1}$. The low proportion of legume in the mixed system may have resulted in low TAGN. The fibrous root systems of the $C_4$ plants compete for superficial moisture and soil nutrients when compared with taproot systems of $C_3$ plants [10]. Santos [12] found values of TAGN for bahiagrass mixed with rhizoma peanut of 6 kg N ha$^{-1}$ harvest$^{-1}$.

There was a SM × GC effect on TAGN (*P* < 0.01; Fig 4). Within of GC, there was difference between SM in the CG1 and GC4 (Fig 4A). The N fertilization increased the TAGN in both GC. The mixed treatment was the lowest TAGN in the GC1, while in the GC4 did not differ from control treatment. Within of SM, there was difference between GC for the control and fertilized treatments, while the mixed treatment did not differ between GC (Fig 4B). The TAGN ranged from 22 to 76 kg N ha$^{-1}$ cycle$^{-1}$, 34 to 113 kg N ha$^{-1}$ cycle$^{-1}$, and 15 to 38 kg N ha$^{-1}$ cycle$^{-1}$ for control, fertilized and mixed treatment, respectively. Greater N yields were found in GC1, being greater than other GC for both the control and fertilized treatments. Considering that TAGN is a product of forage N concentration and herbage accumulation, the N fertilizer directly affected results (Fig 1). There was no difference for TAGN between GC when forage peanut and palisadegrass were analyzed together. Santos [12] reported TAGN ranging from 6 to 40 kg N ha$^{-1}$ harvest$^{-1}$ for rhizoma peanut-bahiagrass mixture and bahiagrass fertilized with 90 kg N ha$^{-1}$ harvest$^{-1}$, respectively.

**Fig 4. Sward management strategies (SM) × grazing cycle (GC) interaction effect on Total aboveground N (TAGN) (mean ± SE bars) during the establishment period.** [‡]Non-significant. Data are means across 2 yr, and four replicates (n = 8), three sward management strategies (SM): Control (palisadegrass without nitrogen fertilizer or legume),

Fertilized (palisadegrass fertilized with 150 kg N ha$^{-1}$, split in three equal applications), or Mixed (palisadegrass-forage peanut mixtures no N fertilizer), and five grazing cycle (GC): GC1 to GC5. Different lowercase letter within GC (A) and SM (B) are different according to the PDIFF procedure by LSD test ($P \leq 0.05$). Sward management strategies: $P = 0.01$, Grazing cycle: $P < 0.01$, Sward management strategies × Grazing cycle: $P < 0.01$.

There was no difference between GC for %Ndfa for forage peanut ($P = 0.14$; Table 4). Values ranged from 79 to 85% in GC1 to GC5. Our study found values similar to the literature. Alencar [14] reported a Ndfa of ~80% for forage peanut. Dubeux [47] reported Ndfa ranging from 67 to 96% for seven rhizoma peanut cultivars. Santos [12] found %Ndfa ranging from 60 to 82% for rhizoma peanut in a monoculture or mixed with bahiagrass. The authors observed that legumes in the mixture presented similar %Ndfa compared with legumes in monocultures.

The BNF varied with GC ($P \leq 0.05$; Table 4), and this was likely linked to the reduction of the leaves. The aboveground biomass is associated with the reduction of BNF [48]. Greater BNF was found in GC1, probably because of better weather conditions for plant growth (Fig 1). The total amount of $N_2$ fixation was 13.5 kg N ha$^{-1}$ (GC1 to GC5). These results are relatively small due to the reduced growth of forage peanut (< 10% of the legume in the botanical composition) in the mixed system and mainly due to slow establishment. The proportion of 20 to 30% legume in the botanical composition of pastures may fix 50 to 100 kg N ha$^{-1}$ yr$^{-1}$ [46]. Jaramillo [13] found BNF ranging from 0 to 2.7 kg N ha$^{-1}$ harvest$^{-1}$ for forage peanut-bahiagrass mixtures. Alencar [14] reported BNF of 3.4 kg N ha$^{-1}$ for forage peanut. The authors concluded that during the legume establishment period, there was a reduced BNF due to the slow growth of the legume. A similar pattern was observed in our study. Other authors reported greater BNF with the legume inclusion in mixed systems [12]; however, some factors, such as difference between peanut cultivars, establishment time, and persistence may have

~~influenced the proportion of forage peanut in the pasture, and this was reflected in the BNF. Additionally, in our study the pasture was seeded in 2014 and the trial performed in November 2016 to March 2017 and December 2017 to April 2018, 2 yr after the seeding. In the mixed management, we did not fertilize with N, and the competition for nutrients between legume and grass may have resulted in low results for both forages. Forage peanut mixed with palisadegrass is considered promising; however, significant competition that negatively affects forage peanut establishment occurs in early stages following planting [14]. Further studies need to evaluate seeding *Arachis* with machinery and applying N fertilization during the early stages after planting to determine if legume to sustaininitialestablishment rate can improve. developee~~

## Conclusions

The small amount of forage peanut in the mixed system presented similar results to the unfertilized palisadegrass in most ~~of the~~ response variables ~~during the establishment period~~. Botanical composition fluctuated according to the ~~grazing~~ growth cycle, with forage peanut disappearance at the beginning of the transition from rainy to dry season. Biological $N_2$ fixation and $TAGN_{FP}$ decreased with the reduction of ~~leaf~~ forage peanut biomass, which wereas affected by periodic dry ~~the different~~ weather conditions during the season. In this study, the forage peanut inclusion was not enough to guarantee better results of the mixed system when compared to the N fertilized palisadegrass. The N fertilization enhanced the ~~pre-grazing~~ herbage biomass ~~and green herbage mass of palisadegrass~~, especially the proportion of leaves.

The evaluation during the ~~grazing~~ growth cycles demonstrated the growth pattern of the grass in a tropical environment as well as forage peanut. However, the legume was less tolerant to lack of water with the beginning of the transition to the dry season. Different management strategies such as the beginning of the grazing ~~cycle before~~ less than 905% of

light interception or lower stubble heights should be employed, considering that legumes ($C_3$) and grasses ($C_4$) require different management~~ mainly during the establishment period~~.


## Acknowledgements

The authors thank the members of UnespFOR (Brazilian forage Team) for the contributions during the field trial setup.


## References


1. Silva VJ, Pedreira CGS, Sollenberger LE, Silva LS, Yasuoka JI, Almeida ICL. Canopy height and nitrogen affect herbage accumulation, nutritive value, and grazing efficiency of 'Mulato II' Brachiariagrass. Crop Sci. 2016; 56:2054–2061. doi: ~~http://dx.doi.org/~~10.2135/cropsci2015.12.0764
2. Boddey RM, Macedo R, Tarré RM, Ferreira E, Oliveira OC, Rezende CP, et al. Nitrogen cycling in *Brachiaria* pastures: The key to understanding the process of pasture decline. Agric. Ecosyst. Environ. 2004; 103:389–403. doi: ~~http://dx.doi.org/~~10.1016/j.agee.2003.12.010
3. Sollenberger LE, Newman YC, Vendramini JMB. General guidelines for managing pastures for dairy cows. Coop. Ext. Serv. Publ. AG162. Inst. Food Agric. Sci., Univ. Florida, Gainesville. 2009.
4. Delevatti LM, Cardoso AS, Barbero RP, Leite RG, Romanzini EP, Ruggieri AC, et al. Effect of nitrogen application rate on yield, forage quality, and animal performance in a tropical pasture. Sci. rep. 2019; 9.7596. doi: ~~https://doi.org/~~10.1038/s41598-019-44138-x


Field Code Changed

Field Code Changed

5. Gerber PJ, Steinfeld H, Henderson B, Mottet A, Opio C, Dijkman J, Falcucci A, Tempio G. Tackling climate change through livestock – A global assessment of emissions and mitigation opportunities. Food and Agriculture Organization of the United Nations (FAO), Rome. 2013.

6. Zaman M, Saggar S, Blennerhassett JD, Singh J. Effect of urease and nitrification inhibitors on N transformation, gaseous emissions of ammonia and nitrous oxide, pasture yield and N uptake in grazed pasture system. Soil Biol. Biochem. 2009; 41:1270–1280. doi:~~.;~~ ~~https://doi.org/~~10.1016/j.soilbio.2009.03.011

7. Zaman M, Nguyen ML, Šimek M, Nawaz S, Khan MJ, Babar MN, et al. Emissions of nitrous oxide ($N_2O$) and di-nitrogen ($N_2$) from the agricultural landscapes, sources, sinks, and factors affecting $N_2O$ and $N_2$ ratios. Greenhouse gases-emission, measurement and management (Ed. Guoxiang Liu), 1-32. 2012.

8. Muir JP, Pitman WD, Dubeux JC, Foster JL. The future of warm-season, tropical and subtropical forage legumes in sustainable pastures and rangelands. Afr. J. Range For. Sci. 2014; 31:187–198. doi: ~~http://dx.doi.org/~~10.2989/10220119.2014.884165

9. Sprent JI. Evolving ideas of legume evolution and diversity: a taxonomic perspective on the occurrence of nodulation. New Phytol. 2007; 174:11–25. doi: ~~http://dx.doi.org/~~10.1111/j.1469-8137.2007.02015.x

10. Muir JP, Pitman WD, Foster JL. Sustainable, low-input, warm-season, grass-legume grassland mixtures: Mission (nearly) impossible? Grass Forage Sci. 2011; 66:301–315. doi: ~~http://dx.doi.org/~~10.1111/j.1365-2494.2011.00806.x

11. Williams MJ, Hammond AC, Kunkle WE, Spreen TH. Stocker Performance on Continuously Grazed Mixed Grass-Rhizoma Peanut and Bahiagrass Pastures. J. Prod. Agric. 1991; 4:19–24. doi: ~~http://dx.doi.org/~~10.2134/jpa1991.0019


Field Code Changed

Field Code Changed

Field Code Changed

Field Code Changed

Field Code Changed

**12.** Santos ER, Dubeux JCB, Sollenberger LE, Blount ARS, Mackowiak C, DiLorenzo N, et al. Herbage responses and biological $N_2$ fixation of bahiagrass and rhizoma peanut monocultures compared with their binary mixtures. Crop Sci. 2018; 58:1–15. doi: ~~http://dx.doi.org/~~10.2135/cropsci2018.02.0128

Field Code Changed

**13.** Jaramillo DM, Dubeux JCB, Mackowiak C, Sollenberger LE, DiLorenzo N, Rowland DL, et al. Annual and perennial peanut mixed with 'Pensacola' bahiagrass in North Florida." Crop Sci. 2018; 58:982–992. doi: ~~http://dx.doi.org/~~10.2135/cropsci2017.09.0542

Field Code Changed

**14.** Alencar NM, Vendramini JMB, Santos AC, Silveira ML, Dubeux JCB, Sousa LF, et al. Herbage characteristics of pintoi peanut and palisadegrass established as monoculture or mixed swards. Crop Sci. 2018; 58:1–7. doi: ~~http://dx.doi.org/~~10.2135/cropsci2017.09.0538

Field Code Changed

**15.** Tamele OH, Sá OAAL, Bernardes TF, Lara MAS, Casagrande DR. Optimal defoliation management of brachiaria grass–forage peanut for balanced pasture establishment. Grass Forage Sci. 2018; 73:522–531. doi: ~~http://dx.doi.org/~~10.1111/gfs.12332

Field Code Changed

**16.** Hernandez M, Argel PJ, Ibrahim MA, Mannetje LT. Pasture production, diet selection and liveweight gains of cattle grazing *Brachiaria brizantha* with or without *Arachis pintoi* at two stocking rates in the Atlantic zone of Costa Rica. Trop. Grassl. 1995; 29:134–141.

**17.** Andrade CMS, Garcia R, Valentim JF, Pereira OG. Productivity, utilization efficiency and sward targets for mixed pastures of marandugrass, forage peanut and tropical kudzu. R. Bras. Zootec. 2012; 41:512–520. doi: ~~http://dx.doi.org/~~10.1590/S1516-35982012000300006

Field Code Changed

**18.** Andrade CMS, Garcia R, Valentim JF, Pereira OG. Response of *Arachis pintoi* to grazing intensity when associated with different grasses. In: International Grassland

Congress, Dublin. Proceedings. Wageningen: Wageningen Academic Publishers, 2005. p. 347.

**19.** Thomas RJ. The role of the legume in the nitrogen cycle of productive and sustainable pastures. Grass Forage Sci. 1992; 47:133–142. doi: 10.1111/j.1365-2494.1992.tb02256.x

~~**18.**~~

~~19.~~**20.** USDA. Soil Survey Staff. 2014. Keys to Soil Taxonomy, 12th ed. USDA-Natural Resources Conservation Service, Washington, DC. 2014.

~~20.~~**21.** van Raij B, Andrade JC, Cantarella H, Quaggio JA. Análise química para avaliação da fertilidade de solos tropicais. IAC, Campinas; 2001. (In Portuguese).

~~**21.** Argel PJ, Pizarro EA. Germplasm case study: *Arachis pintoi*. In.: Pastures for the Tropical Lowlands: CIAT's (Centro International de Agricultura Tropical) Contribution. Cali, Colombia; 1992.~~

**22.** Claessen MEC. Manual de métodos de análise de solo/Centro Nacional de Pesquisa de Solos. 2 ed. rev. Atual. Rio de Janeiro, EMBRAPA-CNPS. Documentos; 1997. (In Portuguese).

**23.** Werner JC, Paulino VT, Cantarella H, Andrade NO, Quaggio JA. Forrageiras. In: van Raij B, Cantarella H, Quaggio JA, Furlani AMC. Recomendações de adubação e calagem para o Estado de São Paulo. Campinas: Instituto Agronômico, 1996p. 263-273. (In Portuguese).

**24.** Allen VG, Batello C, Berretta EJ, Hodgson J, Kothmann M, Li X, et al. An international terminology for grazing lands and grazing animals. Grass Forage Sci. 2011; 66:2–28. doi: ~~http://dx.doi.org/~~10.1111/j.1365-2494.2010.00780.x

**25.** Pereira JC, Gomes FK, Oliveira MD, Lara MA, Bernardes TF, Casagrande DR. Defoliation management affects morphogenetic and structural characteristics of mixed

**Field Code Changed**

pastures of brachiaria grass and forage peanut. Afr. J. Range For. Sci. 2017; 34:13–19. doi: ~~https://doi.org/~~10.2989/10220119.2017.1315960

**26.** Arnold SL, Schepers JS. A simple roller-mill grinding procedure for plant and soil samples. Commun. Soil Sci. Plan. 2004; 35:537–545. doi: ~~http://dx.doi.org/~~10.1081/CSS-120029730

**27.** Shearer G, Kohl D. $N_2$-fixation in field settings: Estimations based on natural $^{15}N$ abundance. Aust. J. Plant Physiol. 1986; 13:699–756. doi: ~~http://dx.doi.org/~~10.1071/PP9860699

**28.** Unkovich M, Herridge D, Peoples M, Cadisch G, Boddey R, Giller K, et al. Measuring plant-associated nitrogen fixation in agricultural systems. In: Unkovich M, Herridge D, Peoples M, Cadisch G, Boddey R, Giller K, et al. Australian Centre for International Agricultural Research (ACIAR), 2008.

**29.** Silva DRG, Costa KAP, Faquin V, Oliveira IP, Bernardes TF. Rates and sources of nitrogen in the recovery of the structural and productive characteristics of marandu grass. Rev. Cienc. Agron. 2013; 44, 184–191. doi: ~~http://dx.doi.org/~~10.1590/S1806-66902013000100023 (Abstract in English)

**30.** Santana SS, Brito LF, Azenha MV, Oliveira AA, Malheiros EB, Ruggieri AC, et al. Canopy characteristics and tillering dynamics of Marandu palisade grass pastures in the rainy–dry transition season. Grass Forage Sci. 2016; 72:261–270. doi: 10.1111/gfs.12234

~~**29.**~~

~~**30.**~~**31.** Chapman SC, Barreto HJ. Using a chlorophyll meter to estimate specific leaf nitrogen of tropical maize during vegetative growth. Agron. J. 1997; 89:557–562. doi: ~~http://dx.doi.org/~~10.2134/agronj1997.00021962008900040004x

**32.** Dubeux JCB, Blount ARS, Mackowiak C, Santos ERS, Pereira Neto JD, Riveros U, et al. Biological $N_2$ fixation, belowground responses, and forage potential of rhizoma peanut cultivars. Crop Sci. 2017; 57:1027–1038. doi: 10.2135/cropsci2016.09.0810

**33.** Carvalho LR, Pereira LET, Hungria M, Camargo PBD, Silva SC. Nodulation and biological nitrogen fixation (BNF) in forage peanut (*Arachis pintoi*) cv. Belmonte subjected to grazing regimes. Agric. Ecosyst. Envir. 2019; 278:96–106. doi: 10.1016/j.agee.2019.02.016

**34.** Phelan P, Moloney AP, McGeough EJ, Humphreys J, Bertilsson J, O'Riordan EG, et al. Forage legumes for grazing and conserving in ruminant production systems. Crit. Rev. Plant Sci. 2015; 34:281–326. doi: 10.1080/07352689.2014.898455

**35.** Andrade CMS, Wadt PGS, Zaninetti RA, Valentim JF. Recomendação de calagem e adubação para pastagens no Acre. Circ. Tec. 46. Embrapa. 2014. Available from: https://ainfo.cnptia.embrapa.br/digital/bitstream/item/115015/1/25378.pdf. (In Portuguese)

**36.** Volenec JJ, Nelson CJ. Responses of Tall Fescue Leaf Meristems to N Fertilization and Harvest Frequency 1. Crop Sci. 1983; 23:720–724. doi: 10.2135/cropsci1983.0011183X002300040028x

~~**31.** Dupas E, Buzetti S, Rabêlo FHS, Sarto AL, Cheng NC, Galindo FS, et al. Nitrogen recovery, use efficiency, dry matter yield, and chemical composition of palisade grass fertilized with nitrogen sources in the Cerrado biome. Aust. J. Crop Sci. 2016; 10:1330–1338. doi: http://dx.doi.org/10.21475/ajcs.2016.10.09.p7854~~

~~**32.** Ribeiro OL, Cecato U, Roma CFC, Faveri JC, Gomes JAN, Barbero ML. Forage production and performance animal in coastcross intercropping or not with *Arachis pintoi*, with or without nitrogen. Acta Sci. Anim. Sci. 2008; 30:371-378. doi: http://dx.doi.org/10.4025/actascianimsci.v30i4.6466 (Abstract in English)~~

**Field Code Changed**

**Field Code Changed**

33. Sanchez J, Vendramini J, Sollenberger LE, Silveira ML, Dubeux JC, Moriel P, et al. Forage characteristics of bermudagrass pastures overseeded with Pintoi peanut and grazed at different stubble heights. Crop Sci. 2018; 58:1808-1816. doi: http://dx.doi.org/10.2135/cropsci2018.01.0007

Field Code Changed

34. Santana SS, Brito LF, Azenha MV, Oliveira AA, Malheiros EB, Ruggieri AC, Reis RA. Canopy characteristics and tillering dynamics of Marandu palisade grass pastures in the rainy-dry transition season. Grass Forage Sci. 2016; 72:261-270. doi: https://doi.org/10.1111/gfs.12234

Field Code Changed

35. Brougham RW. Interception of light by the foliage of pure and mixed stands of pasture plants. Aust. J. Agric. Res. 1958; 9:39–52. doi: https://doi.org/10.1071/AR9580039

Field Code Changed

36. Gomes FK, Oliveira MDBL, Homem BGC, Boddey RM, Bernardes TF, Gionbelli MP, et al. Effects of grazing management in brachiaria grass-forage peanut pastures on canopy structure and forage intake. J. Anim. Sci. 2018; 96:3837–3849. doi: http://dx.doi.org/10.1093/jas/sky236

Field Code Changed

37. Pereira LET, Paiva AJ, Guarda VDÁ, Pereira PM, Caminha FO, Silva SC. Herbage utilisation efficiency of continuously stocked marandu palisade grass subjected to nitrogen fertilisation. Sci. Agríc. 2015; 72:114-123. doi: http://dx.doi.org/10.1590/0103-9016-2014-0013

Field Code Changed

38. Fagundes JL, Fonseca DM, Mistura C, Morais RV, Vitor CMT, Gomide JA, et al. Morphogenetical and structural characteristics of the signalgrass in a nitrogen fertilized pasture evaluated over the seasons of the year. R. Bras. Zootec. 2006; 35:21-29. http://dx.doi.org/10.1590/S1516-35982006000100003. (Abstract in English)

Field Code Changed

39.37. Caminha FO, Silva SC, Paiva AJ, Pereira LET, Mesquita P, Guarda VDA. Stability of tiller population of continuously stocked marandu palisade grass fertilized

with nitrogen. Pesq. Agropec. Bras. 2010; 45:213–220. doi: http://dx.doi.org/10.1590/S0100-204X2010000200013 (Abstract in English)

Field Code Changed

**38.** Calvière I, Duru M. The effect of N and P fertilizer application and botanical composition on the leaf/stem ratio patterns in spring in Pyrenean meadows. Grass Forage Sci. 1999; 54:255–266. doi: 10.1046/j.1365-2494.1999.00178.x

**39.** Fonseca L, Mezzalira JC, Bremm C, Gonda HL, Carvalho PDF. Management targets for maximising the short-term herbage intake rate of cattle grazing in Sorghum bicolor. Livest. Sci. 2012; 145:205–211. doi: 10.1016/j.livsci.2012.02.003

**40.** Sollenberger LE, Burns JC. Canopy characteristics, ingestive behaviour and herbage intake in cultivated tropical grasslands. In: Gomide JA, Mattos WRS, Silva SC, Proceedings of the 19th International Grassland Congress, São Pedro, Brazil. 10–21 Feb. 2001. Brazilian Soc. Anim. Husbandry, Piracicaba, Brazil. 2001, pp. 321–327.

**41.** Pereira JM, Rezende CDP, Borges AMF, Homem BGC, Casagrande DR, Macedo TM, et al. Production of beef cattle grazing on *Brachiaria brizantha* (Marandu grass)-*Arachis pintoi* (forage peanut cv. Belomonte) mixtures exceeded that on grass monocultures fertilized with 120 kg N/ha. Grass Forage Sci. 2020; 75:28–36. doi: https://doi.org/10.1111/gfs.12463

Field Code Changed

**42.** Mullenix MK, Sollenberger LE, Wallau MO, Rowland DL, Blount AR, Vendramini JMB, et al. Sward structure, light interception, and rhizome-root responses of rhizoma peanut cultivars and germplasm to grazing management. Crop Sci. 2016; 56:899–906. doi: http://dx.doi.org/10.2135/cropsci2015.08.0508

Field Code Changed

**43.40.** Burns JC, Sollenberger LE. Grazing behavior of ruminants and daily performance from warm-season grasses. Crop Sci. 2002; 42:873–881. doi: https://doi.org/10.2135/cropsci2002.8730

Field Code Changed

**41.** Euclides VP, Montagner DB, Macedo MCM, Araújo AR, Difante GS, Barbosa RA. Grazing intensity affects forage accumulation and persistence of Marandu palisadegrass in the Brazilian savannah. Grass Forage Sci. 2019; 74:450–462. doi: 10.1111/gfs.12422

**42.** Chen L, Auh C, Chen F, Cheng X, Aljoe H, Dixon RA, et al. Lignin deposition and associated changes in anatomy, enzyme activity, gene expression, and ruminal degradability in stems of tall fescue at different developmental stages. J. Agric. Food Chem. 2002; 50:5558–5565. doi: 10.1021/jf020516x

**~~44.~~43.** Newman YC, Sollenberger LE, Kunkle WE, Chambliss CG. Canopy height and nitrogen supplementation effects on performance of heifers grazing limpograss. Agron J. 2002; 94:1375–1380. doi: ~~https://doi.org/~~10.2134/agronj2002.1375

**44.** Duru M, Ducrocq H. Growth and senescence of the successive leaves on a Cocksfoot tiller. Effect of nitrogen and cutting regime. Ann. Bot. 2000; 85:645-653. doi: 10.1006/anbo.1999.1117

**45.** Gomes FK, Oliveira MDBL, Homem BGC, Boddey RM, Bernardes TF, Gionbelli MP, et al. Effects of grazing management in brachiaria grass-forage peanut pastures on canopy structure and forage intake. J. Anim. Sci. 2018; 96:3837–3849. doi: 10.1093/jas/sky236

**46.** Pereira JM, Rezende CDP, Borges AMF, Homem BGC, Casagrande DR, Macedo TM, et al. Production of beef cattle grazing on *Brachiaria brizantha* (Marandu grass)-*Arachis pintoi* (forage peanut cv. Belomonte) mixtures exceeded that on grass monocultures fertilized with 120 kg N/ha. Grass Forage Sci. 2020; 75:28–36. doi: 10.1111/gfs.12463

**47.** Andrade CMSD, Garcia R, Valentim JF, Pereira OG. Grazing management strategies for massaigrass-forage peanut pastures: 1. Dynamics of sward condition and botanical

**Field Code Changed**

composition. R. Bras. Zootec. 2006; 35:334–342. doi: 10.1590/S1516-35982006000200002

**48.** Ribeiro OL, Cecato U, Roma CFC, Faveri JC, Gomes JAN, Barbero ML. Forage production and performance animal in coastcross intercropping or not with *Arachis pintoi*, with or without nitrogen. Acta Sci. Anim. Sci. 2008; 30:371–378. doi: 10.4025/actascianimsci.v30i4.6466 (Abstract in English)

**49.** Argel PJ, Pizarro EA. Germplasm case study: *Arachis pintoi*. In.: Pastures for the Tropical Lowlands: CIAT's (Centro International de Agricultura Tropical) Contribution. Cali, Colombia; 1992.

~~45.~~**50.** Lima JA, Pinto JC, Evangelista AR, Santana RAV. Amendoim Forrageiro (*Arachis pintoi* Krapov. & Gregory); 2003. Available from: http://livraria.editora.ufla.br/upload/boletim/extensao-tmp/boletim-extensao-001.pdf. (In Portuguese).

**51.** Ruggieri AC, Galzerano L, Silva WL. Interação entre plantas em ambientes de pastagens. In.: Reis RA, Bernardes TF, Siqueira GR (Eds). Forragicultura: Ciência, Tecnologia e Gestão dos Recursos Forrageiro. Jaboticabal: Gráfica e editora multipress; 2013. (In portuguese)

**52.** Homem BG, Rosa AD, Ferreira IM, Cruvinel IA, Lara MA, Bernardes TF, et al. Increasing the population of forage peanut in a mixed pasture by controlling the canopy height. Grass Forage Sci. 2019; 74:571–575. doi: 10.1111/gfs.12436

~~Andrade CMS, Wadt PGS, Zaninetti RA, Valentim JF. Recomendação de calagem e adubação para pastagens no Acre. Circ. Tec. 46. Embrapa. 2014. Available from: https://ainfo.cnptia.embrapa.br/digital/bitstream/item/115015/1/25378.pdf. (In Portuguese)~~

~~Dubeux JCB, Blount ARS, Mackowiak C, Santos ERS, Pereira Neto JD, Riveros U, et al. Biological $N_2$ fixation, belowground responses, and forage potential of rhizoma peanut cultivars. Crop Sci. 2017; 57:1027–1038. doi: http://dx.doi.org/10.2135/cropsci2016.09.0810~~

~~Carvalho LR, Pereira LET, Hungria M, Camargo PBD, Silva SC. Nodulation and biological nitrogen fixation (BNF) in forage peanut (*Arachis pintoi*) cv. Belmonte subjected to grazing regimes. Agric. Ecosyst. Envir. 2019; 278:96–106. doi: https://doi.org/10.1016/j.agee.2019.02.016~~

**Formatted:** Font:

**Formatted:** Font:

**Formatted:** Font:

**Formatted:** Font:

Jaboticabal, Sao Paulo State, Brazil, November 18th, 2020.

Dear Plos One Editor,

Subject: Submission of a revised manuscript PONE-D-20-17268 entitled “Could forage peanut in low proportion replace N fertilizer in livestock systems?”.

Thank you for considering our manuscript “Could forage peanut in low proportion replace N fertilizer in livestock systems?” for publication. We carefully addressed each of the reviewers’ comments, and we hope that the changes will meet your expectations. Our answers are listed below (in red) and a version of the revised manuscript with all the modifications (i.e., corrections and additional information inserted) highlighted in “track-change mode”. We would like to thank you and the reviewers for the excellent review, which certainly improved the manuscript quality.

Thank you for the suggestions and possible appreciation of our work for publication in this renowned journal. The authors are available for other modifications that may improve the quality of the manuscript, as well as additional clarifications.

Sincerely,

Vanessa Zirondi Longhini, Ph.D.
Department of Animal Science
São Paulo State University (UNESP-FCAV)
Jaboticabal (SP), Zip 14884-900, Brazil

## Comments to Author

**Additional Editor Comments:**

**1.** Thank you for submitting your manuscript to PLOS ONE. After careful consideration, we feel that it has merit but does not fully meet PLOS ONE's publication criteria as it currently stands. Therefore, we invite you to submit a revised version of the manuscript that addresses the points raised during the review process. A major problem with the paper – as highlighted by both reviewers – is that the authors goal is to compare the legume-grass mixture with the grass monoculture, but they failed to achieve an adequate amount of legume in the mixture to provide a legitimate test of the mixture's potential. Thus, although they gathered a lot of interesting data, they did not adequately test their hypotheses because their legume-grass mixture had much too little legume to provide a valid test. It may be possible to develop an interesting study even though the pintoi did not perform as expected.

**Authors Response:** Many thanks for considering a new version of the manuscript. After careful revision, we changed the focus of the manuscript. Please, see the hypothesis in the introduction section: "We hypothesized that the forage peanut mixed with non-N fertilized grass would benefit the herbage responses through BNF, compared with unfertilized grass in a monoculture. However, the proportion of forage peanut in the sward in the present study was low. Thus, we decided to test whether there is a meaningful and measurable contribution of the N from forage peanut even at minimal participation and whether this contribution could be close to those systems using synthetic N fertilizer. Therefore, this study evaluated the performance of palisadegrass under three nitrogen management strategies (unfertilized, N fertilized, or mixed with the legume forage peanut), aiming to identify how different N inputs may affect grass responses". We rewrote the results and discussion in separate sections.

**2.** As reviewers suggest, the use the weather and growth cycle data to find correlations (if they exist) and thus an explanation of results.

**Authors Response:** Thank you for this suggestion. We checked the correlations, and they were included in the manuscript.

**3.** There is also a substantial lack of organization, excess detail and insufficient attention to testing of hypotheses that they can achieve, given what the study actually tested. I agree with reviewer two that the results and discussion should be separated, and the need to reduce by about 50% including all redundant measurements, and figures that are redundant with tables. So this is a really major revision needed, in terms of reanalysis and rewriting - just so this is a

clear recommendation and I hope that this is undertaken as the work is important, just challenging given that the original hypotheses were not possible to test due to establishment issues.

**Authors Response:** We fully agree with this critical and useful comments. Please, note that we split the results and discussion section, removed redundant measurements and figures, which was possible to reduce the manuscript.

**Reviewers' comments to author**

**Reviewer 1**

The paper is generally well written. In the attached pdf, there are comment boxes suggesting revisions or asking questions. The biggest issue the authors are dealing with is that the establishment of *pintoi* was not satisfactory. This impacts any conclusions about the three treatments, because you are comparing the two strong grass monocultures against a mixture that does not reflect the true potential of the mixture. The authors try to get around this by saying in one case that they are describing what happens during the establishment period, but in another case they say they are addressing whether there is meaningful and measurable contribution from peanut even at a very low participation. I encourage them to focus the paper on one of these two approaches, not both of them. I think the latter is most plausible. The problem with saying they are looking at what happens during establishment is that the planting occurred in 2014 and the experiment did not start until 2016. Yes, I very well know that Arachis establishes slowly, but to suggest that Years 3 and 4 after planting are the establishment period is a rather large stretch. I suggest that instead they focus on whether there is value even of a small proportion of peanut in the association and do that intentionally and consistently throughout the paper so that the theme is well developed and serves as a point of focus.

**Authors Response:** Thank you very much for your effort in revising our manuscript. All suggestion made in the pdf file was included in the new version. We agree with you on all points raised during the revision process. We are now focusing on the small contribution of *Arachis pintoi* to the system instead of the legume establishment process. Please, note that some sentences and references were removed during the review process to reduce redundancy and be more textually efficient, as suggested by the editor and reviewer 2.

1. This is not true everywhere all the time, so I suggest "The current relationship between beef price and fertilizer in Brazil does not".

**Authors Response:** We appreciate this comment. The sentence was corrected according to the suggestion (line 21).

2. vegetation types implies species or growth habit, suggest using "sward management strategies" as you do later, or perhaps "pasture species composition".

**Authors Response:** Thank you for the suggestion. We changed to "pasture species composition" as suggested (line 24).

3. I prefer "season" because trial implies a different experiment, and you simply repeated the same experiment in two year, at least that is what I understand.

**Authors Response:** We agree with the reviewer. We changed to "season" as suggested (line 196).

4. In-stead of "Herbage structures", I suggest "Herbage and canopy characteristics".

**Authors Response:** Thank you for the suggestion. We have made changes as suggested (line 207).

5. I am not sure what you mean by this sentence. If height is not consistent with LI, then how can you use the same height for comparisons? Expand what you are trying to say here so that it is more clear. What do you mean by lower cost? Easier and cheaper to measure height than LI???

**Authors Response:** We agree with your comment. The sentence was confusing. After careful revision, we removed the sentence from the new version.

6. suggest "herbage accumulation rates in monoculture or mixed pastures". The word especially does not seem to fit when you are discussing more than one item.

**Authors Response:** The sentence was removed in the new version.

7. occurred due to drought conditions.

**Authors Response:** Thank you for the suggestion. We rephrased the sentence in the new version: "Nitrogen fertilization combined with favorable weather conditions promoted greater green biomass (leaf and stem) and reduced the senescent biomass, while the N fertilization and

drought conditions of the transition period between rainy to dry season may have increased the senescent biomass".

8. resulting in lesser green herbage mass (leaf and stem) and greater dead mass in GC5.

**Authors Response:** Thank you for the suggestion. We rephrase the sentence in the new version (Please, see the last answer).

9. interception threshold before the other treatments. This may have resulted in competition for light among tillers, and the plant elongates stem under these conditions in search of light for the younger leaves.

**Authors Response:** Many thanks for your suggestion. We have made changes as suggested (lines 379 and 383-384).

10. season, tillers may have increased in number or weight [34].

**Authors Response:** Removed from the new version.

11. It isn't totally clear to me how you planted in 2014 and started the study in 2016, yet you describe the period of study as the establishment period. It seems that you simply had an inferior stand of peanut, and it was never going to get better.

**Authors Response:** You are correct. We are now focusing on the forage peanut contribution to the system, even at minimal participation. This sentence was removed from the new version.

12. Keep in mind that the mixed treatment had only 90% LI on average, right? So, you would need to reduce this number to considerably less than 90, would you not?

**Authors Response:** We are grateful for this comment. We agree with you. In the mixed system the grazing started at 90% LI, therefore, we changed to "less than 90% of light interception" as suggested (line 805).

**Reviewer 2**

The authors report on the response of palisade pasture with no N inputs, mineral N, or Pintoi peanut (after 2-year establishment phase). The legume did not benefit sward productivity, which was assumed to be due to inadequate establishment length and periodic dry periods across several grazing cycles. Negative results in research are useful if well-delivered and if it addresses important topics. Since mineral N replacement is of great interest in food production,

this topic has value. However, there are some major challenges the authors need to address. Many were included in the attached Word document (see attachment).

**Authors Response:** Thank you very much for all the valuable advice and points raised during the revision process. We hope that all changes will meet your expectations. We included the suggestions made in both files.

Other notable points:

- Experimental design as a Completely Randomized Design. Is that the case? Typically, these experiments are Randomized, Complete Block Design. It might be beneficial to include a figure with the experimental design illustrated.

**Authors Response:** Yes, the experimental design was in a completely randomized design. We included a figure with the experimental design (Figure 2).

- The manuscript was exceptionally long and many of the measurements were variations of key measurements, which made several parts of the Results and Discussion section seem redundant. I think the Conclusions section exemplified the simplicity of the results and the authors should use that as a guide on how to address their results in the main body of the document. Biomass was measured, leaf biomass, stem biomass, green herbage, dead herbage (not sure how that is defined), and daily growth rates. The results were clear but the authors greatly complicate the results with long discussion on the each of these (and several other) measurements that all lead to the same conclusion (the pintoi did not contribute to productivity as much as N fertilizer). Not much of a mystery there! I suggest dropping much of the redundant measurements, figures that are redundant with tables and focus on the key points. The pintoi did not perform as expected and to use the weather and growth cycle data to find correlations (if they exist) and explanation of results. This manuscript can easily be reduce by 50% without impacting the message. Just because you measured lots of things does not mean they all need to be reported on! Pick the key points and focus on them. Keep the reader interested!

**Authors Response:** We agree with you. We removed some redundant data and separated the results from the discussion section to reduce the manuscript. We are now focusing on the small contribution of *Arachis pintoi* to the system instead of the legume establishment process. Correlations were tested and were included as an explanation of the results. The term “dead herbage” was changed to “senescent biomass” and was defined in the Materials and methods section as “senescent biomass (brown leaf and stem attached to the plant)”. We hope that all changes will meet your expectations.

- To help reduce redundancy and be more textually efficient, it may help to structure this manuscript with separate Results and Discussion sections rather than combining them as Results and Discussion. Then the Discussion section can flow freely without stepping through each measurement one by one and repeating the same cause-effect for every dependent variable.

**Authors Response:** Many thanks for your suggestion. Please, note that we separated the Results and Discussion section, in this way, the manuscript was reduced without changing the message.

- Paragraphs are not consistently indented (run-on paragraphs).

**Authors Response:** Thank you for catching it. We revised according to the pdf in the submission guidelines.

- Was not sure how the 3 N fertilizer split applications factored into grass response (growth cycles) in terms of biomass, as some cycles had greater biomass than others. Seems that would impact biomass differently over the season.

**Authors Response:** We split the application to reduce possible N losses through volatilization, leaching, or runoff. However, some losses may have occurred in the first growth cycles due to high rainfall after urea application. However, the biomass in the fertilizer treatment was greater than in the mixed and control treatment. To improve the results visualization, we change the table for the figure (Fig 3). Please note that the fertilized treatment had the same pattern across all growth cycles.

- Make sure any abbreviations used in tables and figures are written out (in title, footnote, caption, as appropriate).

**Authors Response:** Checked.

1. Do not understand the notations, in terms of the different years for each grazing evaluation. Might be easier to follow if presented as a table, with a column for each grazing season.

**Authors Response:** Many thanks for your suggestion. We included the date information in Table 1.

2. This paragraph is probably better suited for the Discussion section.

**Authors Response:** We appreciate this suggestion. We skip the sentence to the Discussion section (lines 422-424).

3. Include the year.

**Authors Response:** We included the year in the revised manuscript.

4. Were the GC equally separated in time? If not, then repeated measures cannot be used. Difficult to determine by the way the sampling dates are presented. The last sampling date seems the most suspicious.

**Authors Response:** The growth cycle was not equally separated because we used the intermittent stocking method, i.e., GC with irregular intervals (Allen et al., 2011). The rest period was interrupted only when the first treatment was achieved at 95% of light interception. We included Table 1 with the date of the growth cycle. Therefore, with your affirmation, we reanalyzed the statistics removing the repeated measures.

5. Why is forage peanut addressed separately? It seems that treatment (regardless of which one) is addressed as a single factor. Not sure I understand why you separate the different treatments (grass ANOVA vs peanut ANOVA).

**Authors Response:** We separated the forage peanut results from the mixed treatment to evaluate the palisadegrass performance among nitrogen management strategies and the forage peanut patterns among the growth cycle. Thus, forage peanut was statistically compared among GC (fixed effect) without the other treatments.

6. You should not imply that there were significant differences when there was not by talking in terms of percent differences. The PHM was not different between control and fertilized grass.

**Authors Response:** Corrected according to the new statistical analyses ($P \leq 0.1$). Please, see lines 373-376.

7. If you had legume growth in the mixture but the sward yield was unchanged, then it must have reduced the grass growth! What you might say instead is that inclusion of the legume did not impact sward productivity compared to grass monoculture.

**Authors Response:** Removed from the new version.

8. Abbreviations need to be defined.

**Authors Response:** Revised.

9. If true then that would be a SM x GC interaction, which you did not have. All you can say is that there was a GC effect and explain what it was. GC response was independent of SM.

**Authors Response:** Revised according to the new statistical analyses ($P \leq 0.1$). Please, see lines 285-290.

10. Unclear how this statement explains the reported response. Either explain more thoroughly or omit the sentence.

**Authors Response:** The sentence was removed.

11. I am certain there are water response literature available to support your supposition about *Arachis pintoi* response to drought or dry soils. We know this to be the case in the US.

**Authors Response:** Thank you for this suggestion. We included two references to support this supposition: "In Northwest Brazil, [17] reported that the drought stress reduced the forage peanut biomass in the mixed system during the dry season. On the other hand, in Northeast Brazil, [46] did not find a negative impact of the dry season on the forage peanut growth. The authors suggested positive legume performance due to the favorable climatic weather in the study region, even during the dry season. Thus, differences among Brazilian regions, weather conditions, and latitude play an important role in the forage peanut growth, mainly in the dry season" (lines 411-417).

12. I think this is more a factor of large variability in your data set. Since the yields were functions of the growth rate and they were significantly different, then it would reason that the growth rate would also differ. You have SM x GC interaction (p=0.1) for HAR. For field studies, sometimes using 0.1 as your limit is acceptable.

**Authors Response:** Many thanks for your suggestion. We are now considering the $P \leq 0.1$ for all variables, including the correlations. Please, see lines 285-292.

13. Not easy to see how Fig 1 (given in dates) relates to the growth response (given as cycle numbers). You should consider how to compare them to better support your argument.

**Authors Response:** Thank you. We changed the figure grouping the date by growth cycle to support the data. We also performed a correlation between rainfall and growth cycle to explain the results.

14. What time of year. It's a real challenge to keep scrolling back to determine when each cycle was compared to the season/date.

**Authors Response:** The greater L:S ratio occurred in the first growth cycle (November 2016 and December 2017) at the onset of the rainy season in Brazil. The growth cycle dates were detailed in Table 1.

15. Basically, the extra N contributed to greater stem growth. That seems reasonable. It seems that extra N would either have no effect or decrease L:S. As part of a sward, there is no benefit for a plant to creates relatively more leaf mass, when it cannot intercept anymore light than what it already has. A quick check, led to this example. I'm sure you can find more relevant references, as well: Grass and Forage Science 54(3):255 – 266 DOI: 10.1046/j.1365-2494.1999.00178.x

**Authors Response:** Thank you for this suggestion. More references was included about the extra N: "The extra N may have contributed to competition for light among tillers, and the plant elongates the stem under these conditions in search of the light for the younger leaves [30]. As part of a canopy sward, there is no benefit for a plant to create relatively more leaf biomass when it cannot intercept anymore light than it already has [38], which may explain the greatest stem proportion and canopy height in the N fertilized treatment without effect on the L:S ratio among N management strategies. In the present study, the rainfall was positively correlated with canopy height (r = 0.23, $P = 0.013$), leaf biomass (r = 0.31, $P < 0.001$), and L:S ratio (r = 0.17, $P = 0.064$) of palisadegrass. Stem elongation is not desirable for grazing animal nutrition, basically for three reasons. First, increasing the stem can reduce the leaf proportion [39]; second, the stem elongation increases the canopy height, which will affect the ingestive animal behavior, such as reducing the number of tongue sweeps, bite volume, and bite weight [39, 40]; and third, the stem acts as a support structure of leaves, which has secondary cell walls, such as lignin, which are undigestible components [41, 42]. These factors can limit the selection of leaf and, consequently, reduces animal gain [43]" (lines 383-396).

16. Why is leaf:stem discussed under “herbage structures” instead of morphological components?

**Authors Response:** Thank you for catching it. We included the leaf:stem on the morphological components.

17. Leaf:stem ratio is derived from these measurements. Therefore, leaf:stem ratio should be placed in this section.

**Authors Response:** You are correct. We included the L:S ratio in the morphological components.

18. I do not understand this argument. I suspect that initiating grazing at 95% light interception was not well maintained rather than canopy heights differing at exactly 95% light interception. I do not think you could have managed the systems that accurately, based upon the information provided. An alternative interpretation is does this mean that canopy height was measured at less than 95% light interception for the unfertilized grass treatment? My interpretation of the data is that the low N treatments (grass or mixture) did not grow as much over the same amount of time as the N fertilized grass. Shows up in biomass and related measurements. No surprise…

**Authors Response:** Thank you for the critical and useful comments. After careful revision, we removed the sentence and included the suggestion (line 380-382).

19. Abbreviations need to be written out the first time they are used in the Materials and Methods or Results and Discussion section, regardless if they were used in the Introduction.

**Authors Response:** We appreciate this comment. We checked the abbreviations in the whole manuscript.

20. You did not have grazing frequency comparisons, but rather repeated grazing (cycles). There is nothing presented that suggests that you were testing different frequencies.

**Authors Response:** We appreciate this comment. We removed the sentence to fix it.

21. This is a key point and brings to question why this study was attempted if the peanut was not established. The message seems to be that in terms of yield, the peanut provides no benefit. IF you had addressed forage quality, animal gain, etc., then perhaps you would have

found benefit of the mixed sward. How many years does it take to declare the peanut fully established in a grass sward?

**Authors Response:** Many thanks for this useful comment. We included a sentence to bring this key point: "Pereira [46] reported that 20.5 to 23.8% of forage peanut mixed with palisadegrass resulted in a liveweight gain per ha 29% greater than in N fertilized system (dose of 120 kg N $ha^{-1}$). Therefore, if our study had addressed nutritive value and animal performance, perhaps we would have found the mixed system benefits even at minimal legume participation" (lines 427-431). Based on Tamele et al. (2018), in similar environmental conditions of this study, after three years of establishment with the canopy height of palisadegrass at 20 cm, it was possible to guarantee a successful forage peanut establishment with 19 to 38% of the legume in the botanical composition. However, we are now focusing on the small contribution of *Arachis pintoi* to the system instead of the legume establishment process.

Tamele OH, Sá OAAL, Bernardes TF, Lara MAS, Casagrande DR. Optimal defoliation management of brachiaria grass–forage peanut for balanced pasture establishment. Grass Forage Sci. 2018; 73:522–531. doi: http://dx.doi.org/10.1111/gfs.12332

22. This is much greater than your reported values, so what can the reader conclude about your system?

**Authors Response:** You are right. However, we rewrote the sentence to support the last answer (Please, see lines 427-429).

23. Maybe true for grasses, particularly those that lose quality, with time between grazing events, but do you expect to the same extent (or at all) with the peanut species? I doubt it. You can use this argument for the grass discussion section.

**Authors Response:** We appreciate this comment, you are correct. We moved the sentence to the grass discussion section (lines 392-393 and 395-396).

24. Across all conditions?

**Authors Response:** Many thanks for your comment. Please, note that we included the sentence: "Under regular weather conditions (rainfall, temperature, and photoperiod), warm-season $C_4$ grasses are typically more competitive than legumes, shifting the balance in mixed swards towards the grass [14]" (lines 405-407).

25. Rhizoma peanut is not "seeded", thus the name "rhizoma" peanut (*Arachis glabrata*). It is sprigged.

**Authors Response:** Thank you for catching it. However, the sentence was removed in the revision.

26. I do not understand what you are trying to say (in the dung?). You have the biomass values. You can simply compare proportions based on biomass.

**Authors Response:** We were trying to say that although the legume proportion in the botanical composition was low, the animals selected the forage peanut to compose their diet, and this affirmation we could perform by the isotopic $^{13}C$ in the dung, which was numerically greater than in the botanical composition. However, we removed the sentence to avoid confusion.

27. Are the last two sentences addressing Reference 45 or your study? This is unclear and the discussion seems to go off-track.

**Authors Response:** The sentence was about our study. This was about the forage peanut selection by grazing animals. As we said in the last question, we were trying to say that although the legume proportion in the botanical composition was low, the animals selected the forage peanut to compose their diet. We could perform this affirmation by the isotopic $^{13}C$ in the dung, which was numerically greater than in the botanical composition (but we did not perform the statistical analyses). Taking into account the digestibility of forage peanut, probably the legume in heifers' diet was greater than in the botanical composition, which occurred by the animal selection. However, we removed the sentence to avoid confusion.

28. Since the proportions don't change (except C5), you can say it in the text and no need for Fig. 3.

**Authors Response:** We are grateful for this comment. We removed the Fig. 3 and wrote the information in the results section: "The botanical composition of forage peanut and palisadegrass in the mixed treatment differed among GC ($P < 0.001$), however, the proportions did not change, except in GC5, which had the lowest forage peanut proportion (2% of the botanical composition). The proportion of forage peanut among GC1 to GC4, ranged between 7 to 9% of the botanical composition" (lines 343-347).

29. This sentence does not sound correct. What do you mean?

**Authors Response:** Thank you for this comment. It is about the amount of herbage mass aboveground, which will affect the biological $N_2$ fixation. We removed the sentence in the new manuscript version.

30. How many stages are there and what is the duration of these stages?

**Authors Response:** Thank you for this comment. In Alencar et al. (2018), the evaluations occurred only at the end of each experimental period, i.e., it was seven and eight months after seeding. However, the sentence was removed from the new manuscript version.

Alencar NM, Vendramini JMB, Santos AC, Silveira ML, Dubeux JCB, Sousa LF, et al. Herbage characteristics of pintoi peanut and palisadegrass established as monoculture or mixed swards. Crop Sci. 2018; 58:1–7. doi: http://dx.doi.org/10.2135/cropsci2017.09.0538
