# Supplementary material for: Could forage peanut in low proportion replace N fertilizer in livestock systems?
Source: PLoS One. 2021 Mar 3;16(3):e0247931. doi: 10.1371/journal.pone.0247931 (PMC7928512; doi:10.1371/journal.pone.0247931)
Supplement: Attachment — Submitted filename: Response to Reviewers.docx [file pone.0247931.s004.docx]

Jaboticabal, Sao Paulo State, Brazil, November 18^th^, 2020.

Dear Plos One Editor,

Subject: Submission of a revised manuscript PONE-D-20-17268 entitled “Could forage peanut in low proportion replace N fertilizer in livestock systems?”.

Thank you for considering our manuscript “Could forage peanut in low proportion replace N fertilizer in livestock systems?” for publication. We carefully addressed each of the reviewers’ comments, and we hope that the changes will meet your expectations. Our answers are listed below (in red) and a version of the revised manuscript with all the modifications (i.e., corrections and additional information inserted) highlighted in “track-change mode”. We would like to thank you and the reviewers for the excellent review, which certainly improved the manuscript quality.

Thank you for the suggestions and possible appreciation of our work for publication in this renowned journal. The authors are available for other modifications that may improve the quality of the manuscript, as well as additional clarifications.

Sincerely,

Vanessa Zirondi Longhini, Ph.D.

Department of Animal Science

São Paulo State University (UNESP-FCAV)

Jaboticabal (SP), Zip 14884-900, Brazil

**Comments to Author**

**Additional Editor Comments:**

**1.** Thank you for submitting your manuscript to PLOS ONE. After careful consideration, we feel that it has merit but does not fully meet PLOS ONE’s publication criteria as it currently stands. Therefore, we invite you to submit a revised version of the manuscript that addresses the points raised during the review process. A major problem with the paper – as highlighted by both reviewers – is that the authors goal is to compare the legume-grass mixture with the grass monoculture, but they failed to achieve an adequate amount of legume in the mixture to provide a legitimate test of the mixture's potential. Thus, although they gathered a lot of interesting data, they did not adequately test their hypotheses because their legume-grass mixture had much too little legume to provide a valid test. It may be possible to develop an interesting study even though the pintoi did not perform as expected.

**Authors Response:** Many thanks for considering a new version of the manuscript. After careful revision, we changed the focus of the manuscript. Please, see the hypothesis in the introduction section: “We hypothesized that the forage peanut mixed with non-N fertilized grass would benefit the herbage responses through BNF, compared with unfertilized grass in a monoculture. However, the proportion of forage peanut in the sward in the present study was low. Thus, we decided to test whether there is a meaningful and measurable contribution of the N from forage peanut even at minimal participation and whether this contribution could be close to those systems using synthetic N fertilizer. Therefore, this study evaluated the performance of palisadegrass under three nitrogen management strategies (unfertilized, N fertilized, or mixed with the legume forage peanut), aiming to identify how different N inputs may affect grass responses”. We rewrote the results and discussion in separate sections.

**2.** As reviewers suggest, the use the weather and growth cycle data to find correlations (if they exist) and thus an explanation of results.

**Authors Response:** Thank you for this suggestion. We checked the correlations, and they were included in the manuscript.

**3.** There is also a substantial lack of organization, excess detail and insufficient attention to testing of hypotheses that they can achieve, given what the study actually tested. I agree with reviewer two that the results and discussion should be separated, and the need to reduce by about 50% including all redundant measurements, and figures that are redundant with tables. So this is a really major revision needed, in terms of reanalysis and rewriting - just so this is a clear recommendation and I hope that this is undertaken as the work is important, just challenging given that the original hypotheses were not possible to test due to establishment issues.

**Authors Response:** We fully agree with this critical and useful comments. Please, note that we split the results and discussion section, removed redundant measurements and figures, which was possible to reduce the manuscript.

**Reviewers' comments to author**

**Reviewer 1**

The paper is generally well written. In the attached pdf, there are comment boxes suggesting revisions or asking questions. The biggest issue the authors are dealing with is that the establishment of *pintoi* was not satisfactory. This impacts any conclusions about the three treatments, because you are comparing the two strong grass monocultures against a mixture that does not reflect the true potential of the mixture. The authors try to get around this by saying in one case that they are describing what happens during the establishment period, but in another case they say they are addressing whether there is meaningful and measurable contribution from peanut even at a very low participation. I encourage them to focus the paper on one of these two approaches, not both of them. I think the latter is most plausible. The problem with saying they are looking at what happens during establishment is that the planting occurred in 2014 and the experiment did not start until 2016. Yes, I very well know that Arachis establishes slowly, but to suggest that Years 3 and 4 after planting are the establishment period is a rather large stretch. I suggest that instead they focus on whether there is value even of a small proportion of peanut in the association and do that intentionally and consistently throughout the paper so that the theme is well developed and serves as a point of focus.

**Authors Response:** Thank you very much for your effort in revising our manuscript. All suggestion made in the pdf file was included in the new version. We agree with you on all points raised during the revision process. We are now focusing on the small contribution of *Arachis pintoi* to the system instead of the legume establishment process. Please, note that some sentences and references were removed during the review process to reduce redundancy and be more textually efficient, as suggested by the editor and reviewer 2.

1. This is not true everywhere all the time, so I suggest "The current relationship between beef price and fertilizer in Brazil does not".

**Authors Response:** We appreciate this comment. The sentence was corrected according to the suggestion (line 21).

2. vegetation types implies species or growth habit, suggest using "sward management strategies" as you do later, or perhaps "pasture species composition".

**Authors Response:** Thank you for the suggestion. We changed to “pasture species composition” as suggested (line 24).

3. I prefer "season" because trial implies a different experiment, and you simply repeated the same experiment in two year, at least that is what I understand.

**Authors Response:** We agree with the reviewer. We changed to “season” as suggested (line 196).

4. In-stead of "Herbage structures", I suggest "Herbage and canopy characteristics".

**Authors Response:** Thank you for the suggestion. We have made changes as suggested (line 207).

5. I am not sure what you mean by this sentence. If height is not consistent with LI, then how can you use the same height for comparisons? Expand what you are trying to say here so that it is more clear. What do you mean by lower cost? Easier and cheaper to measure height than LI???

**Authors Response:** We agree with your comment. The sentence was confusing. After careful revision, we removed the sentence from the new version.

6. suggest "herbage accumulation rates in monoculture or mixed pastures". The word especially does not seem to fit when you are discussing more than one item.

**Authors Response:** The sentence was removed in the new version.

7. occurred due to drought conditions.

**Authors Response:** Thank you for the suggestion. We rephrased the sentence in the new version: “Nitrogen fertilization combined with favorable weather conditions promoted greater green biomass (leaf and stem) and reduced the senescent biomass, while the N fertilization and drought conditions of the transition period between rainy to dry season may have increased the senescent biomass”.

8. resulting in lesser green herbage mass (leaf and stem) and greater dead mass in GC5.

**Authors Response:** Thank you for the suggestion. We rephrase the sentence in the new version (Please, see the last answer).

9. interception threshold before the other treatments. This may have resulted in competition for light among tillers, and the plant elongates stem under these conditions in search of light for the younger leaves.

**Authors Response:** Many thanks for your suggestion. We have made changes as suggested (lines 379 and 383-384).

10. season, tillers may have increased in number or weight [34].

**Authors Response:** Removed from the new version.

11. It isn't totally clear to me how you planted in 2014 and started the study in 2016, yet you describe the period of study as the establishment period. It seems that you simply had an inferior stand of peanut, and it was never going to get better.

**Authors Response:** You are correct. We are now focusing on the forage peanut contribution to the system, even at minimal participation. This sentence was removed from the new version.

12. Keep in mind that the mixed treatment had only 90% LI on average, right? So, you would need to reduce this number to considerably less than 90, would you not?

**Authors Response:** We are grateful for this comment. We agree with you. In the mixed system the grazing started at 90% LI, therefore, we changed to “less than 90% of light interception” as suggested (line 805).

**Reviewer 2**

The authors report on the response of palisade pasture with no N inputs, mineral N, or Pintoi peanut (after 2-year establishment phase). The legume did not benefit sward productivity, which was assumed to be due to inadequate establishment length and periodic dry periods across several grazing cycles. Negative results in research are useful if well-delivered and if it addresses important topics. Since mineral N replacement is of great interest in food production, this topic has value. However, there are some major challenges the authors need to address. Many were included in the attached Word document (see attachment).

**Authors Response:** Thank you very much for all the valuable advice and points raised during the revision process. We hope that all changes will meet your expectations. We included the suggestions made in both files.

Other notable points:

- Experimental design as a Completely Randomized Design. Is that the case? Typically, these experiments are Randomized, Complete Block Design. It might be beneficial to include a figure with the experimental design illustrated.

**Authors Response:** Yes, the experimental design was in a completely randomized design. We included a figure with the experimental design (Figure 2).

- The manuscript was exceptionally long and many of the measurements were variations of key measurements, which made several parts of the Results and Discussion section seem redundant. I think the Conclusions section exemplified the simplicity of the results and the authors should use that as a guide on how to address their results in the main body of the document. Biomass was measured, leaf biomass, stem biomass, green herbage, dead herbage (not sure how that is defined), and daily growth rates. The results were clear but the authors greatly complicate the results with long discussion on the each of these (and several other) measurements that all lead to the same conclusion (the pintoi did not contribute to productivity as much as N fertilizer). Not much of a mystery there! I suggest dropping much of the redundant measurements, figures that are redundant with tables and focus on the key points. The pintoi did not perform as expected and to use the weather and growth cycle data to find correlations (if they exist) and explanation of results. This manuscript can easily be reduce by 50% without impacting the message. Just because you measured lots of things does not mean they all need to be reported on! Pick the key points and focus on them. Keep the reader interested!

**Authors Response:** We agree with you. We removed some redundant data and separated the results from the discussion section to reduce the manuscript. We are now focusing on the small contribution of *Arachis pintoi* to the system instead of the legume establishment process. Correlations were tested and were included as an explanation of the results. The term “dead herbage” was changed to “senescent biomass” and was defined in the Materials and methods section as “senescent biomass (brown leaf and stem attached to the plant)”. We hope that all changes will meet your expectations.

- To help reduce redundancy and be more textually efficient, it may help to structure this manuscript with separate Results and Discussion sections rather than combining them as Results and Discussion. Then the Discussion section can flow freely without stepping through each measurement one by one and repeating the same cause-effect for every dependent variable.

**Authors Response:** Many thanks for your suggestion. Please, note that we separated the Results and Discussion section, in this way, the manuscript was reduced without changing the message.

- Paragraphs are not consistently indented (run-on paragraphs).

**Authors Response:** Thank you for catching it. We revised according to the pdf in the submission guidelines.

- Was not sure how the 3 N fertilizer split applications factored into grass response (growth cycles) in terms of biomass, as some cycles had greater biomass than others. Seems that would impact biomass differently over the season.

**Authors Response:** We split the application to reduce possible N losses through volatilization, leaching, or runoff. However, some losses may have occurred in the first growth cycles due to high rainfall after urea application. However, the biomass in the fertilizer treatment was greater than in the mixed and control treatment. To improve the results visualization, we change the table for the figure (Fig 3). Please note that the fertilized treatment had the same pattern across all growth cycles.

- Make sure any abbreviations used in tables and figures are written out (in title, footnote, caption, as appropriate).

**Authors Response:** Checked.

1. Do not understand the notations, in terms of the different years for each grazing evaluation. Might be easier to follow if presented as a table, with a column for each grazing season.

**Authors Response:** Many thanks for your suggestion. We included the date information in Table 1.

2. This paragraph is probably better suited for the Discussion section.

**Authors Response:** We appreciate this suggestion. We skip the sentence to the Discussion section (lines 422-424).

3. Include the year.

**Authors Response:** We included the year in the revised manuscript.

4. Were the GC equally separated in time? If not, then repeated measures cannot be used. Difficult to determine by the way the sampling dates are presented. The last sampling date seems the most suspicious.

**Authors Response:** The growth cycle was not equally separated because we used the intermittent stocking method, i.e., GC with irregular intervals (Allen et al., 2011). The rest period was interrupted only when the first treatment was achieved at 95% of light interception. We included Table 1 with the date of the growth cycle. Therefore, with your affirmation, we reanalyzed the statistics removing the repeated measures.

5. Why is forage peanut addressed separately? It seems that treatment (regardless of which one) is addressed as a single factor. Not sure I understand why you separate the different treatments (grass ANOVA vs peanut ANOVA).

**Authors Response:** We separated the forage peanut results from the mixed treatment to evaluate the palisadegrass performance among nitrogen management strategies and the forage peanut patterns among the growth cycle. Thus, forage peanut was statistically compared among GC (fixed effect) without the other treatments.

6. You should not imply that there were significant differences when there was not by talking in terms of percent differences. The PHM was not different between control and fertilized grass.

**Authors Response:** Corrected according to the new statistical analyses (*P* ≤ 0.1). Please, see lines 373-376.

7. If you had legume growth in the mixture but the sward yield was unchanged, then it must have reduced the grass growth! What you might say instead is that inclusion of the legume did not impact sward productivity compared to grass monoculture.

**Authors Response:** Removed from the new version.

8. Abbreviations need to be defined.

**Authors Response:** Revised.

9. If true then that would be a SM x GC interaction, which you did not have. All you can say is that there was a GC effect and explain what it was. GC response was independent of SM.

**Authors Response:** Revised according to the new statistical analyses (*P* ≤ 0.1). Please, see lines 285-290.

10. Unclear how this statement explains the reported response. Either explain more thoroughly or omit the sentence.

**Authors Response:** The sentence was removed.

11. I am certain there are water response literature available to support your supposition about *Arachis pintoi* response to drought or dry soils. We know this to be the case in the US.

**Authors Response:** Thank you for this suggestion. We included two references to support this supposition: “In Northwest Brazil, [17] reported that the drought stress reduced the forage peanut biomass in the mixed system during the dry season. On the other hand, in Northeast Brazil, [46] did not find a negative impact of the dry season on the forage peanut growth. The authors suggested positive legume performance due to the favorable climatic weather in the study region, even during the dry season. Thus, differences among Brazilian regions, weather conditions, and latitude play an important role in the forage peanut growth, mainly in the dry season” (lines 411-417).

12. I think this is more a factor of large variability in your data set. Since the yields were functions of the growth rate and they were significantly different, then it would reason that the growth rate would also differ. You have SM x GC interaction (p=0.1) for HAR. For field studies, sometimes using 0.1 as your limit is acceptable.

**Authors Response:** Many thanks for your suggestion. We are now considering the *P* ≤ 0.1 for all variables, including the correlations. Please, see lines 285-292.

13. Not easy to see how Fig 1 (given in dates) relates to the growth response (given as cycle numbers). You should consider how to compare them to better support your argument.

**Authors Response:** Thank you. We changed the figure grouping the date by growth cycle to support the data. We also performed a correlation between rainfall and growth cycle to explain the results.

14. What time of year. It’s a real challenge to keep scrolling back to determine when each cycle was compared to the season/date.

**Authors Response:** The greater L:S ratio occurred in the first growth cycle (November 2016 and December 2017) at the onset of the rainy season in Brazil. The growth cycle dates were detailed in Table 1.

15. Basically, the extra N contributed to greater stem growth. That seems reasonable. It seems that extra N would either have no effect or decrease L:S. As part of a sward, there is no benefit for a plant to creates relatively more leaf mass, when it cannot intercept anymore light than what it already has. A quick check, led to this example. I’m sure you can find more relevant references, as well: [Grass and Forage Science](https://www.researchgate.net/journal/1365-2494_Grass_and_Forage_Science) 54(3):255 – 266 DOI: [10.1046/j.1365-2494.1999.00178.x](https://www.researchgate.net/deref/http%3A%2F%2Fdx.doi.org%2F10.1046%2Fj.1365-2494.1999.00178.x)

**Authors Response:** Thank you for this suggestion. More references was included about the extra N: “The extra N may have contributed to competition for light among tillers, and the plant elongates the stem under these conditions in search of the light for the younger leaves [30]. As part of a canopy sward, there is no benefit for a plant to create relatively more leaf biomass when it cannot intercept anymore light than it already has [38], which may explain the greatest stem proportion and canopy height in the N fertilized treatment without effect on the L:S ratio among N management strategies. In the present study, the rainfall was positively correlated with canopy height (r = 0.23, *P* = 0.013), leaf biomass (r = 0.31, *P* < 0.001), and L:S ratio (r = 0.17, *P* = 0.064) of palisadegrass. Stem elongation is not desirable for grazing animal nutrition, basically for three reasons. First, increasing the stem can reduce the leaf proportion [39]; second, the stem elongation increases the canopy height, which will affect the ingestive animal behavior, such as reducing the number of tongue sweeps, bite volume, and bite weight [39, 40]; and third, the stem acts as a support structure of leaves, which has secondary cell walls, such as lignin, which are undigestible components [41, 42]. These factors can limit the selection of leaf and, consequently, reduces animal gain [43]” (lines 383-396).

16. Why is leaf:stem discussed under “herbage structures” instead of morphological components?

**Authors Response:** Thank you for catching it. We included the leaf:stem on the morphological components.

17. Leaf:stem ratio is derived from these measurements. Therefore, leaf:stem ratio should be placed in this section.

**Authors Response:** You are correct. We included the L:S ratio in the morphological components.

18. I do not understand this argument. I suspect that initiating grazing at 95% light interception was not well maintained rather than canopy heights differing at exactly 95% light interception. I do not think you could have managed the systems that accurately, based upon the information provided. An alternative interpretation is does this mean that canopy height was measured at less than 95% light interception for the unfertilized grass treatment? My interpretation of the data is that the low N treatments (grass or mixture) did not grow as much over the same amount of time as the N fertilized grass. Shows up in biomass and related measurements. No surprise…

**Authors Response:** Thank you for the critical and useful comments. After careful revision, we removed the sentence and included the suggestion (line 380-382).

19. Abbreviations need to be written out the first time they are used in the Materials and Methods or Results and Discussion section, regardless if they were used in the Introduction.

**Authors Response:** We appreciate this comment. We checked the abbreviations in the whole manuscript.

20. You did not have grazing frequency comparisons, but rather repeated grazing (cycles). There is nothing presented that suggests that you were testing different frequencies.

**Authors Response:** We appreciate this comment. We removed the sentence to fix it.

21. This is a key point and brings to question why this study was attempted if the peanut was not established. The message seems to be that in terms of yield, the peanut provides no benefit. IF you had addressed forage quality, animal gain, etc., then perhaps you would have found benefit of the mixed sward. How many years does it take to declare the peanut fully established in a grass sward?

**Authors Response:** Many thanks for this useful comment. We included a sentence to bring this key point: “Pereira [46] reported that 20.5 to 23.8% of forage peanut mixed with palisadegrass resulted in a liveweight gain per ha 29% greater than in N fertilized system (dose of 120 kg N ha^-1^). Therefore, if our study had addressed nutritive value and animal performance, perhaps we would have found the mixed system benefits even at minimal legume participation” (lines 427-431). Based on Tamele et al. (2018), in similar environmental conditions of this study, after three years of establishment with the canopy height of palisadegrass at 20 cm, it was possible to guarantee a successful forage peanut establishment with 19 to 38% of the legume in the botanical composition. However, we are now focusing on the small contribution of *Arachis pintoi* to the system instead of the legume establishment process.

Tamele OH, Sá OAAL, Bernardes TF, Lara MAS, Casagrande DR. Optimal defoliation management of brachiaria grass–forage peanut for balanced pasture establishment. Grass Forage Sci. 2018; 73:522–531. doi: <http://dx.doi.org/10.1111/gfs.12332>

22. This is much greater than your reported values, so what can the reader conclude about your system?

**Authors Response:** You are right. However, we rewrote the sentence to support the last answer (Please, see lines 427-429).

23. Maybe true for grasses, particularly those that lose quality, with time between grazing events, but do you expect to the same extent (or at all) with the peanut species? I doubt it. You can use this argument for the grass discussion section.

**Authors Response:** We appreciate this comment, you are correct. We moved the sentence to the grass discussion section (lines 392-393 and 395-396).

24. Across all conditions?

**Authors Response:** Many thanks for your comment. Please, note that we included the sentence: “Under regular weather conditions (rainfall, temperature, and photoperiod), warm-season C_4_ grasses are typically more competitive than legumes, shifting the balance in mixed swards towards the grass [14]” (lines 405-407).

25. Rhizoma peanut is not “seeded”, thus the name “rhizoma” peanut (*Arachis glabrata*). It is sprigged.

**Authors Response:** Thank you for catching it. However, the sentence was removed in the revision.

26. I do not understand what you are trying to say (in the dung?). You have the biomass values. You can simply compare proportions based on biomass.

**Authors Response:** We were trying to say that although the legume proportion in the botanical composition was low, the animals selected the forage peanut to compose their diet, and this affirmation we could perform by the isotopic ^13^C in the dung, which was numerically greater than in the botanical composition. However, we removed the sentence to avoid confusion.

27. Are the last two sentences addressing Reference 45 or your study? This is unclear and the discussion seems to go off-track.

**Authors Response:** The sentence was about our study. This was about the forage peanut selection by grazing animals. As we said in the last question, we were trying to say that although the legume proportion in the botanical composition was low, the animals selected the forage peanut to compose their diet. We could perform this affirmation by the isotopic ^13^C in the dung, which was numerically greater than in the botanical composition (but we did not perform the statistical analyses). Taking into account the digestibility of forage peanut, probably the legume in heifers’ diet was greater than in the botanical composition, which occurred by the animal selection. However, we removed the sentence to avoid confusion.

28. Since the proportions don’t change (except C5), you can say it in the text and no need for Fig. 3.

**Authors Response:** We are grateful for this comment. We removed the Fig. 3 and wrote the information in the results section: “The botanical composition of forage peanut and palisadegrass in the mixed treatment differed among GC (*P* < 0.001), however, the proportions did not change, except in GC5, which had the lowest forage peanut proportion (2% of the botanical composition). The proportion of forage peanut among GC1 to GC4, ranged between 7 to 9% of the botanical composition” (lines 343-347).

30. This sentence does not sound correct. What do you mean?

**Authors Response:** Thank you for this comment. It is about the amount of herbage mass aboveground, which will affect the biological N_2_ fixation. We removed the sentence in the new manuscript version.

31. How many stages are there and what is the duration of these stages?

**Authors Response:** Thank you for this comment. In Alencar et al. (2018), the evaluations occurred only at the end of each experimental period, i.e., it was seven and eight months after seeding. However, the sentence was removed from the new manuscript version.

Alencar NM, Vendramini JMB, Santos AC, Silveira ML, Dubeux JCB, Sousa LF, et al. Herbage characteristics of pintoi peanut and palisadegrass established as monoculture or mixed swards. Crop Sci. 2018; 58:1–7. doi: <http://dx.doi.org/10.2135/cropsci2017.09.0538>
