# Supplementary material for: Could forage peanut in low proportion replace N fertilizer in livestock systems?
Source: PLoS One. 2021 Mar 3;16(3):e0247931. doi: 10.1371/journal.pone.0247931 (PMC7928512; doi:10.1371/journal.pone.0247931)
Supplement: Attachment — Submitted filename: Response to Reviewers.docx [file pone.0247931.s007.docx]

Jaboticabal, Sao Paulo State, Brazil, February 15^th^, 2021.

Dear PLOS One Editor,

Subject: Submission of a revised manuscript PONE-D-20-17268R1 entitled “Could forage peanut in low proportion replace N fertilizer in livestock systems?”.

Thank you for considering our manuscript “Could forage peanut in low proportion replace N fertilizer in livestock systems?” for publication. We carefully addressed each of the reviewers’ comments, and we hope that the changes will meet your expectations. Our answers are listed below (in red) and a version of the revised manuscript with all the modifications (i.e., corrections and additional information inserted) highlighted in “track-change mode”. We would like to thank you and the reviewers for the excellent review, which certainly improved the manuscript quality.

Thank you for the suggestions and possible appreciation of our work for publication in this renowned journal. The authors are available for other modifications that may improve the quality of the manuscript, as well as additional clarifications.

Sincerely,

Vanessa Zirondi Longhini, Ph.D.

Department of Animal Science

São Paulo State University (UNESP-FCAV)

Jaboticabal (SP), Zip 14884-900, Brazil

**Comments to Author**

**Additional Editor Comments:**

1. The manuscript is much improved and needs modest further inputs in order to be publishable, as indicated by both reviewers. The abstract and conclusion need to be revised to only report on the key findings and not beyond these, and similarly, mean separation should only be conducted if an anova is significant so this section needs to be updated to reflect this.

**Authors Response:** Many thanks for considering a new version of the manuscript. We revised all your concerns and hope that all changes will meet your expectations. We modified the abstract, conclusion, and statistical analyzes to fit in the suggestions.

**Reviewers' comments to author**

**Reviewer 1**

Suggestions for revision are made in the attached pdf file. Most are editorial in nature. Perhaps the one of greatest consequence relates to the last sentence of the Abstract and similar comments in the Conclusion section. These comments address treatments that were not tested in this study, so I don't think they really belong in these critical parts of your document.

**Authors Response:** Thank you very much for your effort in revising our manuscript. We agree with you on all points raised during the revision process. We removed the sentence from the abstract and conclusion section. All suggestion made in the pdf file was included in the new version.

1. Often, oftentimes is a colloquial expression.

**Authors Response:** Corrected.

2. I don't believe you studied either of these treatments, so I don't think you want this to be the conclusion to your experiment. What did you find out relative to your objective?

**Authors Response:** The sentence was removed. We rephrase the sentence: “Overall, benefits from forage peanut were minimal because legume percentage was less than 10%, while N input in the system by N-fertilizer increased palisadegrass herbage biomass”.

3. Where?? In this sentence you are talking about North America and one presumes a temperate environment, but the references are from warm North American climates. Please clarify where this further study is needed.

**Authors Response:** The studies occurred in Florida. We rephrased the sentence: “In the Southeastern United States, mixing grasses and legumes is a more common practice [11] that requires further study [12, 13]”.

4. This is not Mehlich exponent -1, it is Mehlich-1. Mehlich-1 is a type of extractant used in soil testing.

**Authors Response:** Corrected.

5. The first sentence is methods. In the second sentence, do we really want to be talking about a "difference" with a P value of 0.14. I really don't think so.

**Authors Response:** Thank you for catching it. We removed both sentences.

6. when you say cell production, do you mean cell division or elongation, or both?

**Authors Response:** I was referring to cell division. According to Volenec and Nelson (1983) leaf elongation rate was affected by N fertilizer, which may have been linked to cell division, while cell length remains constant.

Volenec JJ, Nelson CJ. Responses of Tall Fescue Leaf Meristems to N Fertilization and Harvest Frequency 1. Crop Sci. 1983; 23:720–724. doi: [10.2135/cropsci1983.0011183X002300040028x](https://doi.org/10.2135/cropsci1983.0011183X002300040028x)

7. clarify here that you are talking about secondary cell walls in stems, not leaves.

**Authors Response:** Thank you for this comment. We rephrased the sentence to clarify: “Stem acts as a leaf support structure with lignified secondary cell and other less digestible components [40, 41], providing stiffness to the stem. Combined, these traits may reduce animal performance [42]”.

8. Upon what do you base this conclusion since you did not evaluate different levels of LI?

**Authors Response:** We agree with your comment. We did not evaluate the light interception level. We rephrase the sentence to clarify: “However, in our study with a minimal proportion of forage peanut, it may be necessary to keep the LI less than 90% in order to reduce grass competition with the forage peanut [47]”. The information about 90% LI was removed from the abstract and conclusion section.

9. or perhaps reduce legume proportion due to greater competition from the grass??

**Authors Response:** The sentence was rephrased to clarify: “Further studies are required to evaluate whether N fertilization applied at key periods (when conditions are more favorable to forage peanut growth than grass growth) could hasten the legume proportion, at least during the initial years. These factors require further research in pursuit of developing sustainable mixed systems that provide quality feed to grazing animals, provide cost reduction to the farmer and lessen the environmental impacts of synthetic N fertilizers”.

**Reviewer 2**

The response to reviewer comments were thorough and the revised manuscript is a big improvement from the original submission. There remain multiple edits that need to be addressed by the authors prior to publication. Review the rules on when means separation tests can be used (i.e., only when the ANOVA test is significant).

**Authors Response:** Thank you very much for all the valuable advice and points raised during the revision process. We hope that all changes will meet your expectations. We included the suggestions made in both files.

1. In all cases, it should be Mehlich-1 (no superscripts).

**Authors Response:** Corrected.

2. To verify that it was P and not P_2_O_5_. To verify it was K and not K_2_O.

**Authors Response:** Thank you for catching it. We used the amount of phosphate (40 kg P ha^-1^) and chloride (50 kg K ha^-1^) for fertilizer calculation. Then we used 83.3 kg K_2_O ha^-1^ and 222.2 kg P_2_O_5_ ha^-1^. We rephrased the sentence to clarify: At the beginning of the grazing trial in both years, 40 kg P ha^-1^ (equivalent to 222.2 kg P_2_O_5_ ha^-1^) and 50 kg K ha^-1^ (equivalent to 83.3 kg K_2_O ha^-1^) were applied.

3. include a more complete address.

**Authors Response:** We included the complete address: “Embrapa Agrobiologia, Seropedica, RJ, Brazil”.

4. You cannot use means separation if the ANOVA is not significant. 0.104 and 0.143 are not significant, so do not run means separation (no letters for CH or FP).

**Authors Response:** Thank you for catching it. We removed this information from the revised manuscript.

5. Not sure what the journal rules are, but typically one will list the definitions in the same order as they are presented in the table.

**Authors Response:** We revised according to journal rules.

6. I do not understand this sentence. I think of botanical composition as proportions, but you follow that proportions only changed at GC 5.

**Authors Response:** The botanical composition included the proportion of the palisadegrass and forage peanut in the mixed treatment. The figure about botanical composition was removed as suggested in the last review process, including only the text in the results section. We rephrased the sentence to clarify: “The botanical composition of forage peanut and palisadegrass in the mixed treatment differed among GCs (*P* < 0.001). However, these proportions did not change among GC1 to GC4 (ranging between 7 to 9% of the legume in the botanical composition), except in GC5, which had the least forage peanut proportion (2% of the botanical composition)”.

7. This reasoning is difficult to follow and you do not explain why the reader should even be interested. So what? Don't fixate on explaining each response but rather what is its contribution to the overall performance of the system? Is taller canopy a benefit or simply a reflection of greater biomass, in general? If you have greater tiller mass, then you can assume the plant is increasing its ground cover, which is beneficial. Also, maybe there is a relationship between greater tiller mass to L:S ratios. Again, it needs to mean something. Just because you measured a difference does not necessarily mean it is a meaningful part of the story.

**Authors Response:** We agree with your comment. The sentence was removed from the new version.

8. You have more dead biomass with the N treatment because you have more living biomass with the N treatment. You need to compare the ratio of senescent to living. If you do that, you will find that actually, the N fertilizer treatment had a much lower ratio of dead to living. That makes total sense, as often, low N fertilization actually hastens plant life cycles and there is greater senescence.

**Authors Response:** We agree with your comment. The senescent proportion represented 24% of total biomass in the N fertilizer treatment, while mixed and control treatment represented 34 and 31%, respectively. We included the sentence: “There was more senescent biomass in the N fertilized treatment because there was more living biomass (leaf + stem). However, when senescent biomass was compared to the living biomass as a ratio, the N fertilizer treatment had a much lower proportion of senescent to living biomass, representing only 24% of the total herbage biomass. In contrast, in the unfertilized and mixed treatments, the senescent biomass represented 31 and 34% of the total herbage mass, respectively. The low N fertilization may have hastened the plant growth cycle which promoted greater senescence”.

9. This has little to do with your results. Your results are that the forage peanut did poorly, but it sounds as though you are daydreaming about what it would be like if the forage peanut performed well. Stick with your results!

**Authors Response:** We agree with your comment. We removed the sentence from the revised manuscript.

10. It is well known that if you apply N fertilizer, you will make the grass competition that much worse. Perhaps it would help only if applied at the beginning of the rainy season, when the forage peanut is already outgrowing (based on what you reported earlier) the grass. Applying N mid-season will likely only favor the grass (many reports on grass/legume competition).

**Authors Response:** We agree with your comment. We removed the sentence from the revised manuscript.

11. Requires a higher resolution image. This one is blurry. This figure is real helpful for understanding the grazing management.

**Authors Response:** We appreciate this comment. We made the changes as suggested. Please, download the image from the PDF document to see the improved resolution in the image.

12. Would be better if fertilized or mixed legend used a pattern instead of a shade, as it is difficult to distinguish between the 2 treatments. Fig. 3: In the figure caption, you need to explain what the data bars represent (mean), along with the error bars and letters. Fig3 b you need to explain the asterisk.

**Authors Response:** We appreciate this comment. We changed the fertilized legend to black and mixed legend to white. The complete figure caption was described in the manuscript. Please, see lines 129-136, 249-257, and 289-294 in the “Revised Manuscript with Track Changes file”.

13. I do not see any comparisons among GCs even though it was highly significant.

**Authors Response:** Thank you for your comment. We included the GC means in Figure 4.
